# Motor Rehabilitation Provides Modest Functional Benefits After Intracerebral Hemorrhage: a Systematic Review and Meta-Analysis of Translational Rehabilitation Studies

**DOI:** 10.1007/s12975-023-01205-w

**Published:** 2023-11-20

**Authors:** Britt A. Fedor, Noam H. Sander, Maxwell MacLaren, Lane J. Liddle, Crystal L. MacLellan, Frederick Colbourne

**Affiliations:** 1 Neuroscience and Mental Health Institute, Faculty of Medicine and Dentistry, University of Alberta, Edmonton, Canada; 2 Department of Psychology, Faculty of Science, University of Alberta, Edmonton, Canada; 3 Department of Physical Therapy, Faculty of Rehabilitation Medicine, University of Alberta, Edmonton, Canada

**Keywords:** Stroke, Intracerebral hemorrhage, Rehabilitation, Motor recovery, Meta-analysis, Translational research

## Abstract

Few certainties exist regarding the optimal type, timing, or dosage of rehabilitation after stroke. Despite differing injury mechanisms and recovery patterns following ischemic and hemorrhagic stroke, most translational stroke research is conducted after ischemia. As we enter the era of personalized medicine, exploring subtype-specific treatment efficacy is essential to optimizing recovery. Our objective was to characterize common rehabilitation interventions used after in vivo preclinical intracerebral hemorrhage (ICH) and assess the impact of post-ICH rehabilitation (vs. no-rehabilitation) on recovery of motor function. Following PRISMA guidelines, a systematic review (Academic Search Complete, CINAHL, EMBASE, Medline, PubMed Central) identified eligible articles published up to December 2022. Risk of bias (SYRCLE) and study quality (CAMARADES) were evaluated, and random-effects meta-analysis was used to assess treatment efficacy in recovery of forelimb and locomotor functions. Thirty articles met inclusion criteria, and 48 rehabilitation intervention groups were identified. Most used collagenase to model striatal ICH in young, male rodents. Aerobic exercise, enriched rehabilitation, and constraint-induced movement therapy represented ~ 70% of interventions. Study quality was low (median 4/10, range 2–8), and risk of bias was unclear. Rehabilitation provided modest benefits in skilled reaching, spontaneous impaired forelimb use, and locomotor function; however, effects varied substantially by endpoint, treatment type, and study quality. Rehabilitation statistically improves motor function after preclinical ICH, but whether these effects are functionally meaningful is unclear. Incomplete reporting and variable research quality hinder our capacity to analyze and interpret how treatment factors influence rehabilitation efficacy and recovery after ICH.

**Supplementary Information:**

The online version contains supplementary material available at 10.1007/s12975-023-01205-w.

## Introduction

Stroke is a leading cause of death and disability worldwide [1], with > 12 million cases reported annually [2]. Despite representing only 10–20% of all cases, hemorrhagic stroke is responsible for ~ 60% of the global burden of stroke [3, 4]. Intracerebral hemorrhage (ICH), caused by the rupture of cerebral vasculature and bleeding into the brain, is particularly devastating due to high mortality and disability. Analysis of burden of disease by stroke subtype highlights the disproportionate impact of ICH: while ischemia is associated with 4.6–5.9 disability-adjusted-life-years (DALYs) [5], ICH ranges from 8.1 to 12.6 DALYs [6]. Although advances in the diagnosis, treatment, and management of stroke have led to decreased mortality in recent decades, 50–60% of survivors live with persistent impairment or disability [4, 7]. Tasks that require high levels of dexterity or motor coordination can be challenging for survivors, as ~ 80% will experience some degree of transient or permanent paresis in one or more limbs [8]. As a result, many survivors live with impairments that limit participation in activities of daily living, making functions that enable independence (e.g., walking, reaching, grasping, and using the impaired limb) common targets of rehabilitation.

Neurorestorative interventions, such as physical and occupational therapy, attempt to harness principles of experience dependent plasticity to restore, recruit, and retrain circuitry in the injured brain [9], thereby improving function and lessening disability. Several motor rehabilitation interventions have been used in preclinical settings to gain insight into functional and neurological recovery after ICH. Environmental enrichment (EE; or enriched housing) is social housing that introduces novel elements (e.g., toys, tubes, running wheels, ramps, multiple levels) to create a more stimulating cage environment. Early studies of EE after ischemic stroke found that treated rodents showed greater behavioral recovery compared to those in social housing alone or solo housing with running wheel access [10]. Skilled reach training (REACH) uses massed practice of forelimb fine motor skills through repetition of tasks like the Montoya Staircase test [11], tray task [12], and single pellet reaching task [12]. Two related therapies, forced limb use (FLU) and constraint-induced movement therapy (CIMT), involve restraint of the unimpaired limb to encourage use of the impaired limb, thereby preventing learned non-use [13]. Unlike FLU, CIMT pairs restraint with task specific training (e.g., REACH) and/or an exercise (EX) battery (e.g., REACH, wheel running, ladder walking) to maximize treatment efficacy through massed practice. Aerobic exercise (AE) is a running-based intervention; under voluntary exercise conditions, animals have free access to a running apparatus (e.g., running wheel) over a designated period. In contrast, under forced exercise, animals are placed into a running apparatus (e.g., treadmill, rotarod, rotating wheel) where the device is set to a prespecified speed or distance over the intervention period. Enriched rehabilitation (ER) combines REACH with EE to synergize the effects of both therapies [14]. Finally, acrobatic training (AT; or motor skills training) is a complex rehabilitation paradigm comprised of elevated rope ladder walking, elevated grid platform walking, traversing a thick rope, traversing parallel bars, and crossing a series of irregular platforms [15].

Current guidelines recommend that all individuals begin rehabilitation once medically stable and able to actively participate in treatment [16]. However, despite numerous promising clinical trials and preclinical studies exploring rehabilitation after stroke, few certainties exist regarding optimal treatment type, timing, or dose [17]. Clinical studies must often rely on surrogate measures to explore mechanisms of recovery, making preclinical studies often better suited for exploration of mechanisms, in part owing to the complexity, cost, and ethics of conducting such research in patients. Although an essential component of post-stroke care for all patients, most insight into treatment and recovery after stroke has been gained from animal models of cerebral ischemia [18]. These studies provide evidence for a critical period after stroke where endogenous repair processes are heightened and rehabilitation interventions are most effective [19], a phenomenon later supported with clinical evidence [20]. Preclinical studies have also demonstrated that early and intense rehabilitation can exacerbate injury and worsen functional outcomes after cortical lesion, likely triggered by use-dependent responses [21, 22]. However, when given with a short delay, others have reported that a critical threshold of intensity must be met for rehabilitation to mediate functional recovery [23]. No single experimental model of injury can perfectly reproduce the heterogeneous clinical pathology and presentation of stroke; therefore, these experimental findings may not hold true across all types of brain injury. Although some have explored the effect of post-stroke rehabilitation on functional recovery, neuroprotection, or neuroplasticity [24–26], a comprehensive review of the effects of post-stroke rehabilitation on recovery of motor function has not been conducted for preclinical ICH. Owing to fundamental differences in mechanisms of injury between ischemia and ICH (e.g., greater role of mechanical injury and neurotoxicity in ICH) and the differential impact of additional mediators (e.g., post-stroke fever) [27], calls for subtype specific exploration of treatment and rehabilitative therapies are well justified [28, 29].

This systematic review and meta-analysis aimed to (1) identify and characterize common motor rehabilitation interventions used after preclinical in vivo models of ICH; (2) assess the scientific and translational quality of this literature; and (3) analyze the efficacy of post-ICH rehabilitation on recovery of motor function.

## Methods

Our search protocol was developed using PRISMA guidelines and adapted from the PICOS (Patients, Intervention, Comparison, Outcomes, Study designs) framework and registered with PROSPERO (CRD42021227134). The search strategy was developed to identify all articles that used an animal population to model ICH (P), a post-stroke motor rehabilitation intervention (I), compared to no-treatment (C), and evaluated rehabilitation efficacy in at least one motor outcome (O) following experimental induction of ICH (S).

### Search Strategy

An electronic records search of the databases Academic Search Complete, Medline, EMBASE, CINAHL, and PubMed Central was completed on March 12, 2021, and again December 14, 2022, to identify all eligible records published up to December 14, 2022. Search terms (Table 1) were compiled by subject: rehabilitation, stroke type, and population. To ensure accuracy, search term formatting was tailored to each database (see Supplemental Information). Results were entered into Covidence software (Veritas Health Innovation, Melbourne, Australia) and duplicates were removed. Two reviewers (BF, MM/FC) screened titles and abstracts against a priori criteria (Table 2); articles proceeded to full-text review in cases of disagreement. Full-text review was completed by two reviewers (BF, MM/FC); disagreements were discussed, and if agreement was not reached a tie-breaking vote was completed (FC).

**Table 1:** Search term keywords

Search	Term	Keywords
S1	Rehabilitation	rehabilitation OR rehab OR exercise OR motor-therapy OR physical-therap* OR physiotherap* OR aerobic-training OR running OR walking OR treadmill* OR constraint-induced-movement-therapy OR mobilization OR mobilisation OR forced-use-therapy OR enrichment OR environmental-enrichment OR enriched-rehabilitation OR training OR reach* OR grasp*
S2	Stroke type	cerebral-hemorrhage* OR cerebral-haemorrhage* OR intracerebral-hemorrhage* OR intracerebral- haemorrhage* OR intracranial-hemorrhage* OR intracranial-haemorrhage* OR intracerebral-bleed OR cerebral-hematoma* OR hemorrhagic-stroke* OR haemorrhagic-stroke*
S3	Population	rat OR rats OR mouse OR mice OR rodent* OR primate OR canine OR murine OR non-human OR animal-model

**Table 2:** Inclusion and exclusion criteria for abstract and full-text screening

Screening	Include	Exclude
Abstract	1. Stroke type is ICH 2. Study type is animal 3. Therapy is motor rehabilitation intervention	1. Stroke type is not ICH 2. Not an animal study 3. No behavioral intervention *If unclear from abstract, study continued to full text review
Full text	1. Stroke type is ICH 2. Study type is animal 3. Therapy is motor rehabilitation intervention 4. Motor outcome assessment present post-treatment 5. Control group present 6. Full text available in English	1. Stroke type is not ICH 2. Not an animal study 3. No post-stroke behavioral intervention 4. No motor outcome assessment post-treatment 5. No appropriate comparator group present 6. Full text unavailable in English

### Inclusion and Exclusion Criteria

Articles that used an in vivo animal model of ICH regardless of species, strain, co-morbidities, or ICH model were eligible; clinical studies, in vitro studies, or in vivo studies that did not include motor assessment or were completed in a non-ICH animal model of stroke were ineligible. If rehabilitation was delivered pre-stroke, not a motor intervention, failed to assess motor function (e.g., learning or memory task, physiological outcome), or was paired with an adjuvant treatment (e.g., drug, hypothermia), it was ineligible. Articles that did not have an appropriate comparator group (i.e., no treatment group), only compared to another rehabilitation intervention, or were unavailable in English were excluded.

### Data Extraction

Descriptive characteristics were extracted by one reviewer (BF) and validated by a second (MM/NS). Extracted characteristics included author names, publication year, animal population (species, strain, sex, age, co-morbidities), ICH model, anesthetic, survival time(s), use of blinding and randomization, rehabilitation type, behavioral outcomes measured (e.g., reaching success, ladder walking error rate, spontaneous forelimb use), and histological outcomes of severity (i.e., lesion volume, hematoma volume). Treatment parameters (i.e., type, timing, period, duration, frequency, intensity, and dose) were operationalized (Table 3) to create a standardized terminology for interpreting and extracting data [30]. Extracted parameters were used to calculate total treatment dose and reported as the total number of repetitions or running distance achieved over the intervention period and total time in treatment (i.e., hours).

**Table 3:** Standardized terminology and definitions for preclinical rehabilitation interventions

Parameter	Definition	Report as
Type	Activities/tasks that make up the intervention and how they are delivered	Descriptive characteristics
Timing	Onset of rehabilitation after ICH	Hours, days, or weeks after stroke induction (ICH surgery = day 0)
Period	Time over which the intervention occurs (between onset and end of therapy)	Hours, days, or weeks
Duration	The length of a single treatment session, defined for each activity/task in the intervention	Mins, hours, days, or weeks
Frequency	How often the treatment was administered within the treatment period, defined for each activity/task in the intervention	Sessions/day and days/week
Intensity	A measure that provides an estimate of treatment participation/exertion, defined for each activity/task in the intervention	AE: walking/running speed (m/s), walking/running distance (m) EE: time in enrichment (hours/day) FLU: time in restraint (hours/day) REACH: number of repetitions (average number of pellets retrieved per trial) AT: walking distance (m), number of repetitions ER: see EE + REACH EX: see AE, REACH; may also require walking distance (m) and/or number of repetitions CIMT: see FLU + REACH
Total Treatment Dose	The total amount of treatment received over the intervention period, reported for each activity/task in the intervention; calculated using treatment period, duration, frequency, and intensity	AE: total walking/running distance (m) EE: total time in enrichment (hours) FLU: total time in restraint (hours) REACH: total number of repetitions completed (pellets retrieved) AT: total walking distance (m), total number of repetitions ER: see EE + REACH EX: see AE, REACH; may also require total walking distance (m) and/or total number of repetitions CIMT: see FLU + REACH

Efficacy of post-ICH rehabilitation on motor function was our primary meta-analytical endpoint. A motor outcome was eligible for meta-analysis if data were available from ≥ 3 articles that assessed the same domain of recovery (e.g., skilled reaching) using the same or equivalent tasks (e.g., reaching success in the staircase task or single pellet task). Motor outcomes were grouped into forelimb, locomotor, and composite neurobehavioral assessments. Forelimb assessments included skilled reaching success (e.g., staircase test, single pellet task) and spontaneous use of the impaired forelimb (i.e., cylinder task). Locomotor assessments included walking success (e.g., success or error rate in ladder walking, beam walking score), walking speed, and distance traveled. Composite neurobehavioral assessments included global impairment rating scales such as the neurological deficit score (NDS), motor deficit score (MDS), and modified neurological severity score (mNSS). While test batteries and scoring systems differ among these assessments, all rate performance in multiple tests to create a single score representing impairment across several functional domains (e.g., paw asymmetry, grip strength, mobility, balance, response to stimuli). Mean and standard deviation (SD) were extracted for all treatment and control groups for parametric data (i.e., skilled reaching, ladder walking), with median and interquartile range (IQR) extracted for non-parametric data (i.e., beam walking, composite neurobehavioral tests). When data was not explicitly reported, values were measured and calculated from figures using WebPlotDigitizer (version 4.6, Ankit Rohatgi, 2022).

As reporting multiple experiments or intervention groups within the same article is common in preclinical rehabilitation, group sizes, treatment parameters, outcomes, and timing of outcome assessment were extracted for each intervention within an article that met inclusion criteria. When multiple intervention groups were extracted from an article, groups were identified as Author (year)a, Author (year)b, etc.

### Study Quality and Risk of Bias

Study quality was assessed using the CAMARADES checklist [31], with articles rated as yes, unclear, or no for their compliance. Two small modifications were made to the checklist to adapt it for our use: blinded ICH-induction or post ICH-randomization (checklist item 4) and inclusion of comorbidities relevant to ICH (checklist item 7) such as old age, hypertension, and diabetes. The SYRCLE Risk of Bias tool and accompanying signaling questions were used to judge each article across multiple domains of bias [32]. Articles were rated for each domain as low, unclear, or high risk. Caregiver blinding (performance bias) was not rated, as it is near impossible for preclinical researchers to be blinded to rehabilitation delivery. A rating of unclear was given when reviewers deemed there was insufficient and/or inconsistent reporting of detail to accurately judge compliance with the checklist item or signaling question. For both CAMARADES and SYRCLE assessments, two independent reviewers (BF/NS) rated each article, with rating disagreements resolved through discussion. Owing to inclusion of several articles from the authors’ laboratory, FC was excluded from the assessment process to prevent unpublished details from influencing reviewer judgements.

### Statistical Analysis

Statistical analyses were conducted in R (v.4.3.0; R Core Team, 2023) using RStudio (v.2023.3.1.446; Posit Team, 2023) and the tidyverse [33], meta [34], and dmetar packages [35, 36]. Due to variations in experimental designs and intervention protocols, effect sizes were calculated using random effects meta-analysis with the DerSimonian-Laird estimator and the inverse variance method for weighting. Subgroups were determined by intervention type (AT, AE, CIMT + FLU, ER, REACH)—if an intervention type was explored in < 3 articles, it was relegated to OTHER. Rehabilitation efficacy was assessed overall and by subtype for three domains of motor recovery: skilled reaching, spontaneous impaired forelimb use, and locomotor function. To account for small samples sizes and variations in methodology, skilled reaching and ladder walking effect sizes were calculated as Hedge’s *G* standardized mean difference (SMD) with 95% confidence intervals (CI) [37]. When necessary (i.e., when ladder data was reported as error rates), a correction factor of − 1 was applied to the data to maintain consistency in direction of effect across interventions [37]. As all articles that assessed spontaneous impaired forelimb use in the cylinder task reported results as percent impaired forelimb use, effect sizes were calculated as mean difference (MD) with 95% CI. Egger regression was used to assess asymmetry in the funnel plots and possible publication bias; trim-and-fill analysis was conducted if asymmetry was detected. A priori sensitivity analyses were conducted to evaluate the impact of study quality on both treatment efficacy and heterogeneity in our results—interventions from articles that scored < 4 on the CAMARADES checklist were removed and the updated datasets were re-analyzed as above. To explore the impact of experimental design and treatment parameters on rehabilitation efficacy, secondary analyses were completed for each endpoint using subgroups differentiated by timing of treatment onset, stroke severity, total treatment dose, and CAMARADES score.

## Results

**Fig. 1:**
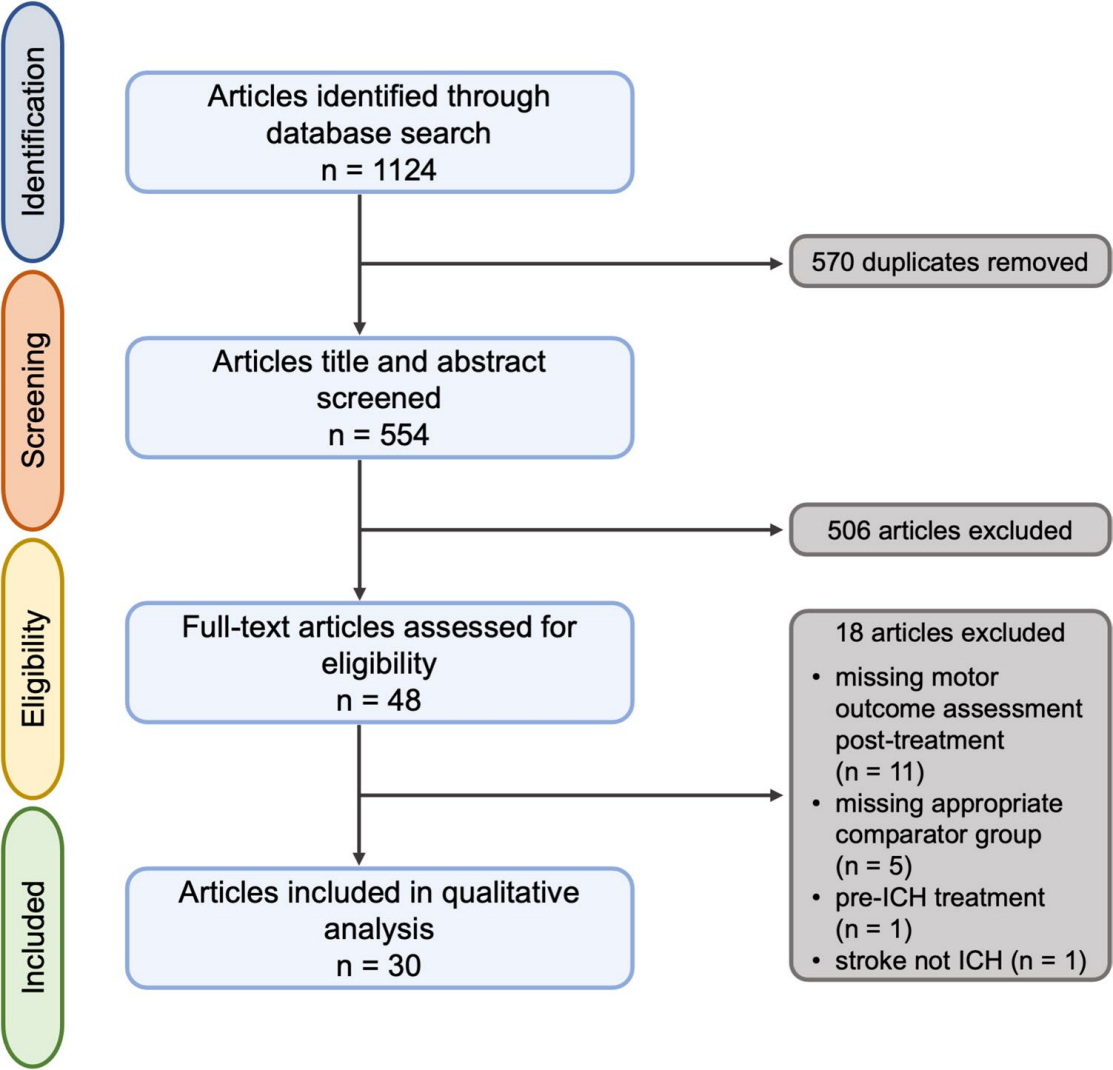
PRISMA flowchart of records identified through database searching

Our search identified 1124 articles (944 March 2021, 180 December 2022). Following screening and full-text review, 30 articles met the eligibility criteria (Fig. 1).

### Descriptive Characteristics

**Fig. 2:**
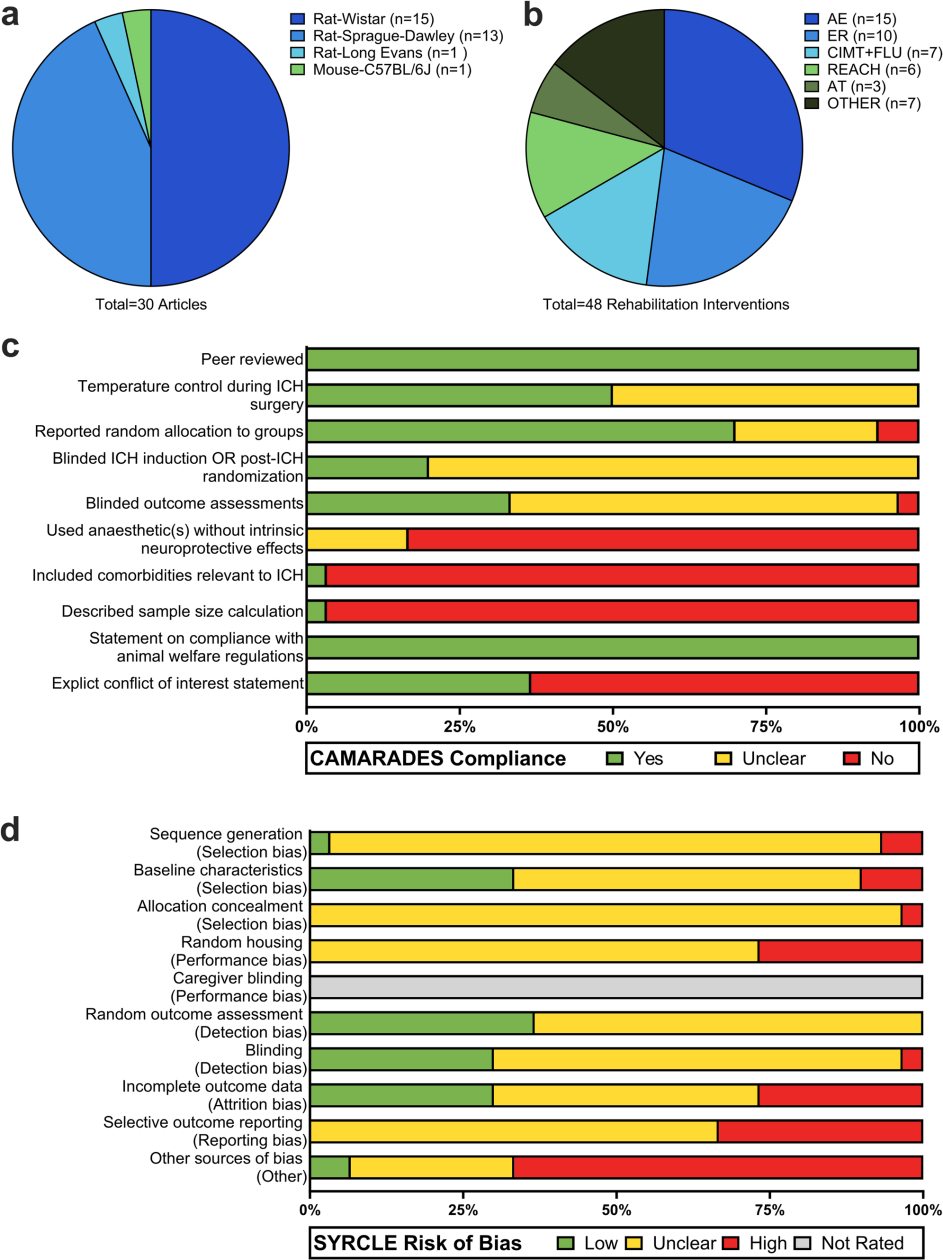
Summary of experimental characteristics, study quality, and risk of bias in eligible articles (*n* = 30). **a** Model population (species and strain); no article reported use of multiple species or strains. **b** Breakdown of the types of rehabilitation interventions (*n* = 48) used after preclinical ICH where 48 unique intervention groups were identified across 30 articles. **c** Summary of article quality assessed by compliance with 10 item CAMARADES checklist (*n* = 30). Article quality ranged considerably (2–8), with a median score of 4. **d** Summary of SYRCLE risk of bias tool (*n* = 30). Risk of bias was predominately unclear, as articles often lacked sufficient detail to determine how/if risk of bias was minimized

Experimental design characteristics were generally homogenous (Table S1). All used rodents (Fig. 2a) < 1 year old; 28/30 used males, whereas 1 used females, and 1 was unspecified. The collagenase model was heavily favored (29/30) over the autologous whole blood model, with injury predominately targeting the striatum (26/30). Thirteen articles reported ≥ 2 eligible intervention groups (i.e., not confounded by adjuvant treatments), resulting in the identification of 48 rehabilitation interventions that assessed efficacy of post-ICH rehabilitation on motor recovery (Fig. 2b). Histological assessment of stroke severity (lesion or hematoma volume) was included in the methods of 34/48 interventions; however, we could not identify the full range of stroke severity studied as results were often unreported or unclear (e.g., assessed in one brain slice). Rehabilitation interventions were grouped into six categories: AE [38–47], ER [48–53], CIMT + FLU [54–58], REACH [59–62], AT [15, 63, 64], and OTHER (complex exercise [54], EE [65], walking [59, 61], swimming [66]). Treatment onset ranged from 6 h to 17 days post-ICH. Most interventions assessed efficacy in ≥ 2 behavioral endpoints (35/48); timing of latest functional efficacy assessment ranged from 25 h to 60 days. Total time spent in treatment ranged from 2 h to 49 days. Table 4 describes the modifiable treatment parameters, total treatment dose, largest group size analyzed in functional endpoints, and comparator group for each of the 48 interventions.

**Table 4:** Descriptive characteristics of rehabilitation interventions

Author year	Type (*n*)	Intervention description	Onset (period)	Frequency	Duration (session)	Intensity	Treatment dose	Control, housing (*n*)	Lesion location (volume)
DeBow 2003a [54 ]	FLU (*n *= 9)	Unimpaired limb restrained	7 days (7 days)	FLU (1 continuous session daily)	FLU (8 h)	FLU (8 h/day)	FLU (56 h)	ICH, solo housed (*n* = 11*) *shared DeBow 2003 a,b,c	Striatum (44 mm^3^ at 60 days)
DeBow 2003b [54]	CIMT (*n *= 11)	Unimpaired limb restrained + exercise (REACH (tray task), cylinder, ladder walking, wheel running)	7 days (7 days)	FLU (1 continuous session daily) EX (1 session daily)	FLU (8 h) EX* (1 h) *REACH (30 min); cylinder (10 min); ladder walking (10 min); AE (10 min)	FLU (8 h/day) EX* (varied) *REACH (repetitions NR); cylinder (NR); ladder walking (3 × 1 m ladder crosses/session); AE (10 m in 10 min)	FLU (56 h) EX* (7 h) *REACH (repetitions NR; 3.5 h); cylinder (70 min); ladder walking (21 m, 70 min); AE (70 m, 70 min)	ICH, solo housed (*n* = 11*) *shared DeBow 2003 a,b,c	Striatum (44 mm^3^ at 60 days)
DeBow 2003c [54]	EX (*n *= 9)	Exercise (REACH (tray task), cylinder, ladder walking, wheel running)	7 days (7 days)	EX (1 session daily)	EX* (1 h) *REACH (30 min); cylinder (10 min); ladder walking (10 min); AE (10 min)	EX* (varied) *REACH (repetitions NR); cylinder (NR); ladder walking (3 × 1 m ladder crosses/session); AE (10 m in 10 min)	EX* (7 h) *REACH (repetitions NR; 3.5 h); cylinder (70 min); ladder walking (21 m, 70 min); AE (70 m, 70 min)	ICH, solo housed (*n* = 11*) *shared DeBow 2003 a,b,c	Striatum (44 mm^3^ at 60 days)
MacLellan 2005 [55]	CIMT (*n *= 15)	Unimpaired limb restrained + exercise (REACH (tray task), wheel running)	14 days (7 days)	FLU (1 continuous session daily) EX (1 session daily)	FLU (8 h) EX* (1 h) *REACH (30 min); AE (30 min)	FLU (8 h/day) EX* (varied) *REACH (repetitions NR); AE (speed NR)	FLU (56 h) EX* (7 h) *REACH (repetitions NR, 3.5 h); AE (distance NR, 3.5 h)	ICH, housing unknown (*n* = 15)	Striatum (81 mm^3^ at 60 days)
Auriat 2006 [38]	AE (*n *= 17)	Forced running (motorized wheel)	14 days (14 days)	AE (1 session daily, 5 days/week)	AE (60 min)	Week 1 (5.5 m/min for 60 min), week 2 (5.5 m/min for 5 min, 11 m/min for 55 min)	AE (4812.5 m, 10 h)	ICH, solo housed (*n* = 17)	Striatum (91 mm^3^ at 49 days)
Auriat 2008 [48]	ER (*n *= 14)	EE housing + Exercise (REACH (tray task), beam walking)	5 days (EE), 7 days (EX) (25 days (EE), 5 days (EX)	EE (1 continuous session) EX (1 session daily day 7, 9, 11)	EE (24 h) EX* (30 + mins) *REACH (30 min); beam walking (NR)	EE (24 h/day (excluding training)) EX* (varied) *REACH (repetitions NR); beam walking (5 × 1.1 m beam crosses/session)	EE (~ 600 h) EX* (~ 1.5 h) *REACH (repetitions NR, 1.5 h); beam walking (16.5 m, time NR)	ICH, group housed (*n* = 15)	Striatum (40 mm^3^ at 30 days)
Nguyen 2008 [65]	EE (*n *= 14)	EE housing	7 days (49 days)	EE (1 continuous session)	EE (24 h)	EE (24 h/day)	EE (1176 h)	ICH, group housed (*n* = 16)	Striatum (68 mm^3^ at 57 days)
Auriat 2009 [49]	ER (*n *= 16)	EE housing + REACH (modified Montoya staircase)	7 days (14 days)	EE (1 continuous session daily) REACH (4 sessions daily, 5 days/week)	EE (15 h) REACH (15 min)	EE (15 h/day) REACH (repetitions NR)	EE (150 h) REACH (repetitions NR, 10 h)	ICH, pair housed (*n* = 16)	Striatum (32 mm^3^ at 46 days)
Auriat 2010a [50]	ER (*n *= 13)	EE housing + REACH (modified Montoya staircase)	7 days (14 days)	EE (1 continuous session daily) REACH (4 sessions daily, 5 days/week)	EE (15 h) REACH (15 min)	EE (15 h/day) REACH (repetitions NR)	EE (150 h) REACH (repetitions NR, 10 h)	ICH, group housed (*n* = 13)	Striatum (not assessed)
Auriat 2010b [50]	ER (*n *= 16)	EE housing + REACH (modified Montoya staircase)	7 days (14 days)	EE (1 continuous session daily) REACH (4 sessions daily, 5 days/week)	EE (15 h) REACH (15 min)	EE (15 h/day) REACH (repetitions NR)	EE (150 h) REACH (repetitions NR, 10 h)	ICH, group housed (*n* = 16)	Striatum (28 mm^3^ at 32 days)
Takamatsu 2010 [39]*	AE (*n *= NR)	Forced running (treadmill)	4 days (11 days)	AE (1 session daily)	AE (30 min)	AE (9 m/min)	AE (2970 m, 5.5 h)	ICH, housing unknown (*n* = NR)	Striatum (~ 60% of striatal volume lost at 15 days)
Ishida 2011 [56]	FLU (*n *= 8)	Unimpaired limb restrained	24 h (7 days)	FLU (1 continuous session)	FLU (24 h)	FLU (24 h/day)	FLU (168 h)	ICH, group housed (*n* = 9)	Internal capsule (7 mm^3^ at 37 days)
MacLellan 2011 [51]	ER (*n *= 16)	EE housing + REACH (modified Montoya staircase)	7 days (14 days)	EE (1 continuous session daily) REACH (4 sessions daily with 2-h interval, 5 days/week)	EE (15 h) REACH (15 min)	EE (15 h/day) REACH (repetitions NR)	EE (150 h) REACH (repetitions NR, 10 h)	ICH, group housed (*n* = 14)	Striatum (~ 8% tissue loss in ipsilesional hemisphere at 49 days)
Mestriner 2011a [59]	REACH (*n *= 12)	REACH (modified Montoya staircase)	7 days (28 days)	REACH (1 session daily, 5 days/week)	REACH (40 min)	REACH (repetitions NR)	REACH (repetitions NR, 13 h 20 min)	ICH, group housed (*n* = 12*) *shared Mestriner 2011a,b	Striatum (56 mm^3^ at 28 days)
Mestriner 2011b [59]	WALK (*n *= 12)	Walk training (treadmill)	7 days (28 days))	WALK (1 session daily, 5 days/week)	WALK (40 min)	WALK (1.8 m/min)	WALK (1440 m, 13 h 20 min)	ICH, group housed (*n* = 12*) *shared Mestriner 2011a,b	Striatum (56 mm^3^ at 28 days)
Kim 2012a [60]*	REACH (*n *= 15)	REACH (single pellet task)	Unclear (unclear)	REACH (1 session daily, 6 days/week)	REACH (15 min)	REACH (repetitions NR)	Cannot determine	ICH, housing unknown (*n* = 15*) *shared Kim 2012a,b	Striatum (lesion ~ 12% of total brain volume, timing unclear)
Kim 2012b [60]*	REACH-ipsi (*n *= 15)	REACH (single pellet task—unimpaired paw)	Unclear (unclear)	REACH (1 session daily, 6 days/week)	REACH (15 min)	REACH (repetitions NR)	Cannot determine	ICH, housing unknown (*n* = 15*) *shared Kim 2012a,b	Striatum (lesion ~ 12% of total brain volume, timing unclear)
Santos 2013a [61]	REACH (*n *= 8)	REACH (single pellet task)	7 days (28 days)	REACH (1 session daily, 5 days/week)	REACH (40 min)	REACH (repetitions NR)	REACH (repetitions NR, 13 h 20 min)	ICH, group housed (*n* = 8*) *shared Santos 2013a,b	Striatum (unclear – analysis conducted in one tissue slice)
Santos 2013b [61]	WALK (*n *= 8)	Walk training (treadmill)	7 days (28 days)	WALK (1 session daily, 5 days/week)	WALK (40 min)	WALK (1.8 m/min)	WALK (1440 m, 13 h 20 min)	ICH, group housed (*n* = 8*) *shared Santos 2013a,b	Striatum (unclear – analysis conducted in one tissue slice)
Caliaperumal 2014 [52]	ER (*n *= 11)	EE housing + REACH (modified Montoya staircase)	7 days (14 days)	EE (1 continuous session daily) REACH (4 sessions daily with 2-h interval, 5 days/week)	EE (15 h) REACH (15 min)	EE (15 h/day) REACH (repetitions NR)	EE (150 h) REACH (repetitions NR, 10 h)	ICH, group housed (*n* = 11)	Striatum (not assessed)
Tamakoshi 2014 [15]*	AT (*n *= 6)	Traverse 5 acrobatic courses (rope ladder, platform grid, single rope, parallel bars, series of barriers)	4 days (25 days)	AT (4 sessions daily)	AT (NR)	AT (5 courses/session, 1 m/course)	AT (500 m (100 crosses/course), time NR)	ICH, housing unknown (*n* = 8)	Striatum (unclear – likely reporting error)
Yong 2014a [62]*	REACH (*n* = NR)	REACH (single pellet task)	NR (unclear)	REACH (1 session daily, 6 days/week)	REACH (15 min)	REACH (repetitions NR)	Cannot determine	ICH, housing unknown (*n* = NR)	Striatum (not assessed)
Yong 2014b [62]*	REACH (*n *= NR)	REACH (single pellet task)	NR (unclear)	REACH (1 session daily, 6 days/week)	REACH (15 min)	REACH (repetitions NR)	Cannot determine	ICH, housing unknown (*n* = NR)	Striatum (not assessed)
Ishida 2015a [57]	FLU (*n* = 8)	Unimpaired limb restrained	24 h (7 days)	FLU (1 continuous session)	FLU (24 h)	FLU (24 h/day)	FLU (168 h)	ICH, housing unknown (*n* = 9)	Globus pallidus (8 mm^3^, timing unclear)
Ishida 2015b [57]	FLU (*n *= 6)	Unimpaired limb restrained	17 days (7 days)	FLU (1 continuous session)	FLU (24 h)	FLU (24 h/day)	FLU (168 h)	ICH, housing unknown (*n* = 9)	Globus pallidus (8 mm^3^, timing unclear)
Ishida 2016 [58]	FLU (*n *= 7)	Unimpaired limb restrained	24 h (7 days)	FLU (1 continuous session)	FLU (24 h)	FLU (24 h/day)	FLU (168 h)	ICH, group housed (*n* = 6)	Internal capsule (not assessed)
Takamatsu 2016 [40]	AE (*n* = 14)	Forced running (treadmill)	4 days (11 days)	AE (1 session daily)	AE (30 min)	AE (9 m/min)	AE (2970 m, 5.5 h)	ICH, housing unknown (*n* = 14)	Striatum (not assessed)
Tamakoshi 2016 [63]	AT (*n *= 6)	Traverse 5 acrobatic courses (rope ladder, platform grid, single rope, parallel bars, series of barriers)	4 days (25 days)	AT (4 sessions daily)	AT (NR)	AT (5 courses/session, 1 m/course)	AT (500 m (100 crosses/course), time NR)	ICH, group housed (*n* = 7)	Striatum (not assessed)
Tamakoshi 2017 [64]	AT (*n *= 6)	Traverse 5 acrobatic courses (rope ladder, platform grid, single rope, parallel bars, series of barriers)	4 days (25 days)	AT (4 sessions daily)	AT (NR)	AT (5 courses/session, 1 m/course)	AT (500 m (100 crosses/course), time NR)	ICH, housing unknown (*n* = 6)	Striatum (assessed, NR)
Tamakoshi 2018a [41]*	AE (*n *= 8)	Forced running (treadmill)	2 days (14 days)	AE (1 session daily)	AE (30 min)	AE (9 m/min day 1, 11 m/min remainder)	AE (4560 m, 7 h)	ICH, housing unknown (*n* = 8*) *shared Tamakoshi 2018 a,b,c	Striatum (assessed, NR)
Tamakoshi 2018b [41]*	AE (*n *= 6)	Forced running (treadmill)	2 days (7 days)	AE (1 session daily)	AE (30 min)	AE (9 m/min day 1, 11 m/min remainder)	AE (2250 m, 3.5 h)	ICH, housing unknown (*n* = 8*) *shared Tamakoshi 2018 a,b,c	Striatum (assessed, NR)
Tamakoshi 2018c [41]*	AE (*n *= 6)	Forced running (treadmill)	8 days (7 days)	AE (1 session daily)	AE (30 min)	AE (9 m/min day 1, 11 m/min remainder)	AE (2250 m, 3.5 h)	ICH, housing unknown (*n* = 8*) *shared Tamakoshi 2018 a,b,c	Striatum (assessed, NR)
Sato 2020a [42]	AE (*n *= 8)	Forced running (treadmill)	4 days (25 days)	AE (4 sessions daily with 60 min interval)	AE (30 min)	AE (10 m/min)	AE (30,000 m, 50 h)	ICH, housing unknown (*n* = 10*) *shared Sato 2020a,b	Striatum (not assessed)
Sato 2020b [42]	AE (*n* = 8)	Voluntary running (wheel in home cage)	4 days (25 days)	AE (1 continuous session)	AE (600 h)	AE (mean distance 1224 ± 86 m/day)	AE (30 600 m, 600 h)	ICH, housing unknown (*n* = 10*) *shared Sato 2020a,b	Striatum (not assessed)
Tamakoshi 2020a [43]*	AE (*n* = 23)	Forced running (treadmill)	2 days (7 days)	AE (1 session daily)	AE (60 min)	AE (9 m/min day 1, 11 m/min remainder)	AE (4500 m, 7 h)	ICH, group housed (*n* = 24*) *shared Tamakoshi 2020a,b	Striatum (unclear – likely reporting error)
Tamakoshi 2020b [43]*	AE (*n* = 22)	Forced running (treadmill)	9 days (7 days)	AE (1 session daily)	AE (60 min)	AE (9 m/min day 1, 11 m/min remainder)	AE (4500 m, 7 h)	ICH, group housed (*n* = 24*) *shared Tamakoshi 2020a,b	Striatum (unclear – likely reporting error)
Xu 2020a [44]	AE (*n* = 11)	Forced running (treadmill)	2 days (13 days)	AE (1 session daily)	AE (30 min)	AE (16 m/min)	AE (6240 m, 6.5 h)	ICH, housing unknown (*n* = 11*) *shared Xu 2020a,b	Striatum (not assessed)
Xu 2020b [44]	AE (*n* = 11)	Forced running (treadmill – fatigue controlled)	2 days (13 days)	AE (1 session daily)	AE (30 min*) *if animal exceeded fatigue threshold, 3 min rest, then session continued (repeated until 30 min running achieved)	AE (16 m/min)	AE (6240 m, 6.5 h)	ICH, housing unknown (*n* = 11*) *shared Xu 2020a,b	Striatum (not assessed)
Tamakoshi 2021 [45]	AE (*n *= 13)	Forced running (treadmill)	6 h (1 day)	AE (2 sessions in first 24 h post-ICH (6 and 24 h))	AE (60 min)	AE (9 m/min at 6 h post-ICH, 11 m/min at 24 h post-ICH)	AE (1200 m, 2 h)	ICH, pair housed (*n* = 14)	Striatum (hematoma ~ 12% of total brain volume at 27 h)
Fedor 2022a [53]	ER (*n *= 13)	EE housing + REACH (modified Montoya staircase) *all interventions in light phase of housing cycle	5 days (10 days)	EE (1 continuous session daily) REACH (4 sessions daily with 1.5-h interval, days 5–12, 2 sessions daily with 1.5-h interval days 13–14)	EE (6 h) REACH (15 min)	EE (6 h/day) REACH (mean pellets retrieved 118/session)	EE (60 h) REACH (mean pellets retrieved 4248, 9 h)	ICH, group housed in light phase (*n* = 9)	Striatum (hematoma volume 20 μL at 14 days)
Fedor 2022b [53]	ER (*n *= 13)	EE housing + REACH (modified Montoya staircase) *all interventions in dark phase of housing cycle	5 days (10 days)	EE (1 continuous session daily) REACH (4 sessions daily with 1.5-h interval, days 5–12, 2 sessions daily with 1.5-h interval days 13–14)	EE (6 h) REACH (15 min)	EE (6 h/day) REACH (mean pellets retrieved 107/session)	EE (60 h) REACH (mean pellets retrieved 3852, 9 h)	ICH, group housed in dark phase (*n* = 11)	Striatum (hematoma volume 16 μL at 14 days)
Fedor 2022c [53]	ER (*n* = 23)	EE housing + REACH (modified Montoya staircase) *all interventions in dark phase of housing cycle	5 days (10 days)	EE (1 continuous session daily) REACH (4 sessions daily with 1.5-h interval)	EE (6 h) REACH (15 min)	EE (6 h/day) REACH (mean pellets retrieved 110/session)	EE (60 h) REACH (mean pellets retrieved 4400, 10 h)	ICH, group housed in dark phase (*n* = 23*) *shared Fedor 2022c,d	Striatum (38 mm^3^ at 60 days)
Fedor 2022d [53]	ER (*n *= 21)	EE housing + REACH (modified Montoya staircase) *all interventions in dark phase of housing cycle	5 days (24 days)	EE (1 continuous session daily) REACH (4 sessions daily with 1.5-h interval days 5–14, 19–28)	EE (6 h) REACH (15 min)	EE (6 h/day) REACH (mean pellets retrieved 121/session days 5–14, 134/session days 19–28)	EE (120 h) REACH (mean pellets retrieved 10 200, 20 h)	ICH, group housed in dark phase (*n* = 23*) *shared Fedor 2022c,d	Striatum (38 mm^3^ at 60 days)
Inoue 2022 [46]	AE (*n *= 8)	Forced running (treadmill)	7 days (21 days)	AE (1 session daily, 5 days/week)	AE (30 min)	AE (12 m/min)	AE (5400 m, 7.5 h)	ICH, housing unknown (*n* = 8)	Internal capsule (14 mm^3^ at 29 days)
Li 2022a [66]*	SWIM (*n *= 10)	Continuous swimming	2 days (7 days)	SWIM (1 session daily)	SWIM (30 min)	NR	SWIM (3.5 h)	ICH, group housed (*n* = 10)	Striatum (unclear – likely reporting error)
Li 2022b [66]*	SWIM (*n *= 10)	Continuous swimming	2 days (14 days)	SWIM (1 session daily)	SWIM (30 min)	NR	SWIM (7 h)	ICH, group housed (*n* = 10)	Striatum (unclear – likely reporting error)
Li 2022c [66]*	SWIM (*n *= 12)	Continuous swimming	2 days (7 days)	SWIM (1 session daily)	SWIM (30 min)	NR	SWIM (3.5 h)	ICH, group housed (*n* = 12)	Striatum (unclear – likely reporting error)
Tamakoshi 2022 [47]	AE (*n *= 14)	Forced running (treadmill)	6 h (6 days)	AE (2 sessions in first 24 h post-ICH (6 and 24 h), 1 session daily days 2–6)	AE (60 min)	AE (9 m/min at 6 h post-ICH, 11 m/min for remainder)	AE (4500 m, 7 h)	ICH, pair housed (*n* = 16)	Striatum (~ 8% of total brain volume at 8 days)

[39]* group sizes for behavioral endpoints not reported

[60]* imprecise timeline, treatment dose could not be calculated

[15]* lesion data reported as volume of tissue lost, but appears to be volume of tissue remaining or based on an unspecified region of interest

[62]* group sizes not reported, total *N* listed as 30 in abstract and 20 in methods; imprecise timeline, treatment dose could not be calculated

[43]* lesion data is uninterpretable

[66]* calculation of hematoma volume appears to be off by a factor of 100–1000; unclear if hematoma volume is calculated as % brain or % hemisphere

*AE*, aerobic exercise; *AT*, acrobatic training; *CIMT*, constraint-induced movement therapy; *EE*, environmental enrichment; *ER*, enriched rehabilitation; *FLU*, forced limb use; *m*, meters; *MDS*, motor deficit score; *mNSS*, modified neurological severity score; *NR*, not reported; *NDS*, neurological deficit score; *REACH*, skilled reach training; *REACH-ipsi*, skilled reach training in unimpaired forelimb; *SWIM*, swim training; *WALK*, walk training

### Study Quality

The CAMARADES checklist analysis revealed a wide range in scores (2–8), with a median score of 4/10, indicating low or unclear study quality (Fig. 2c). Interrater reliability was high (weighted kappa = 0.912), indicating almost perfect agreement between reviewers. All articles (30/30) scored a point for peer review and including a statement on compliance to animal welfare regulations. Temperature control during ICH induction was reported in 15/30 articles, with the remainder unclear. Use of randomization was reported in 21/30 articles. A conflict-of-interest statement was reported in 11/30 articles. Blinding was inconsistent and often vague or poorly described. Only 6/30 articles reported blinding to treatment allocation during stroke induction or that animals were randomized to treatment after stroke. Similarly, only 10/30 articles explicitly reported blinded assessment of subjective outcomes (e.g., neurological deficit assessments, walking errors, lesion volume); many articles were judged unclear, due to poor reporting and/or inconsistent use of blinding across endpoints. Only 1/30 articles included a population with comorbidity (ovariectomized female rats, menopause). Likewise, 1/30 articles described the use of a sample size calculation. No article used an anesthetic without potential neuroprotective properties [67]. Individual article ratings are in Fig. S1.

### Risk of Bias

Analysis using the SYRCLE tool revealed unclear risk of bias in many articles (Fig. 2d). Assessment of interrater reliability indicated substantial agreement between reviewers (weighted kappa = 0.753). While most articles addressed one or more factors related to selection, performance, detection, attrition, or reporting bias, information was often insufficient to determine how risk of bias was minimized (see Table 5 for common errors). Several articles reported manipulating housing conditions as part of treatment (e.g., EE housing). Based on SYRCLE guidelines, these articles received a high-risk rating for performance bias—however, we would argue that this is less indicative of a high risk of performance bias, but rather an intended treatment effect. Many articles failed to adequately address incomplete outcome data, resulting in our attrition bias assessment being approximately equal among each category of low, unclear, or high risk of bias. These judgements were driven by unclear reporting of total N, group sizes, exclusions, and mortality, resulting in insufficient data to judge risk of bias. Approximately one-third of articles were rated high risk of reporting bias due to selective outcome reporting. No articles reported the use of a pre-registered protocol (judged as unclear), whereas many failed to report data from some groups and/or specific endpoints or assessment times and were rated as high risk. Other potential sources of bias identified included unit of analysis errors, improper use of statistical methods, and poor methodology. Individual article ratings are in Fig. S2.

**Table 5:** Commonly observed errors in preclinical ICH rehabilitation literature

Category	Incidence	Error
Reporting errors	17%	Failure to report total number of animals used (*N*)
	40%	Failure to report housing conditions (i.e., solo, paired, group, EE)
	93%	Failure to describe the method of randomization/allocation to group/subgroup (e.g., random number generator)
	60%	Failure to explicitly report exclusions and mortality (including group identity and reason for exclusion)
	17–23%*	Failure to report group sizes (*n*) used in analysis after mortality/exclusion and division into subgroups/endpoints
	7–13%*	Methods describe endpoints not presented in the results (or vice versa)
	10%	Type of summary statistics not provided (e.g., mean ± SD)
Methodological errors	97%	Group size not determined using a power calculation
	20–47%*	Total *N* reported is not equal to the total of *n*’s reported in methods
	20–47%*	Addition of extra subjects (i.e., sum total *n* > *N*)
	57%	Baseline data not assessed and/or not reported
	23–30%*	Data not collected and/or presented for subjects/groups in one or more endpoint with no explanation
	20–23%*	Inappropriate methods of statistical analysis (i.e., using parametric tests on non-parametric data, only comparing to sham, ignoring significant baseline differences, handling of outliers)
	53–93%*	Unit of analysis errors related to housing (i.e., analyzing animals as independent samples instead of by cage)
	47%	Improper data presentation and/or reporting (i.e., figures uninterpretable, missing error terms, describing ordinal data with mean ± SD)

^*^lower value of range represents % of articles with confirmed error while upper value represents % of articles possibly containing error but with insufficient reporting to conclusively determine

### Efficacy of Rehabilitation on Skilled Reaching

**Fig. 3:**
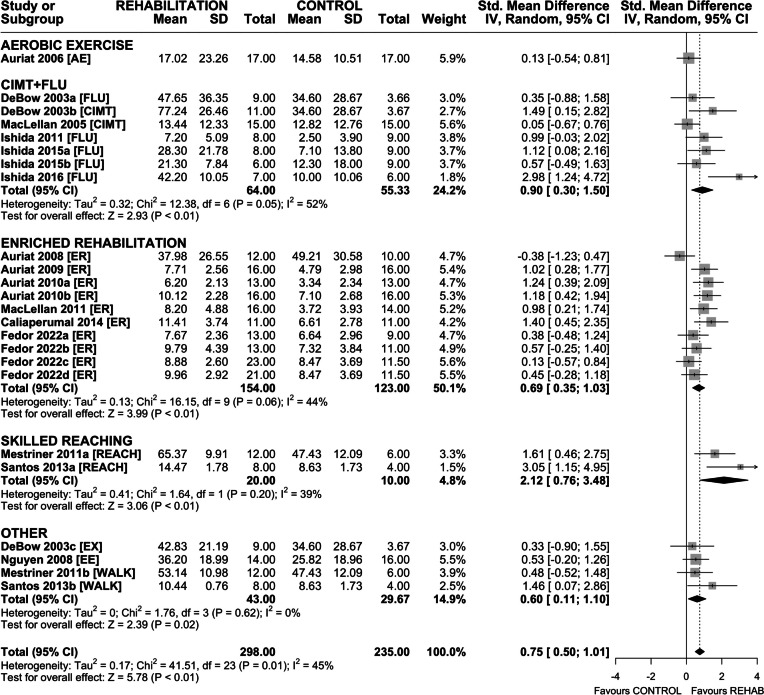
Forest plot of random-effects meta-analysis of post-ICH rehabilitation on performance in skilled reaching tasks (*n* = 24). Rehabilitation significantly improved skilled reaching (SMD 0.75 (95% CI 0.50–1.01), *p* < 0.01), with REACH and CIMT + FLU associated with the largest treatment effects. Egger regression indicated the presence of asymmetry in the dataset. Trim-and-fill analysis added 5 data points, all with SMD < 0, suggesting that null or negative data was missing in our original model. Follow-up random-effects meta-analysis of the trim-and-fill model (*n* = 29) produced a noticeably smaller treatment effect (SMD 0.59 (95% CI 0.32–0.87), *p* < 0.01). Effect sizes presented as Hedge’s *G* standardized mean difference (SMD) with 95% CI

Twenty-four interventions assessed efficacy of rehabilitation on recovery of skilled reaching (Fig. 3). Overall, rehabilitation improved skilled reaching (SMD 0.75 (95% CI 0.50–1.01), *p* < 0.01) and heterogeneity was moderate (*I*^2^ = 45%, *p* = 0.01). Subgroup analysis by rehabilitation type found a significant effect of CIMT + FLU (SMD 0.90 (95% CI 0.30–1.50), *p* < 0.01), ER (SMD 0.69 (95% CI 0.35–1.03), *p* < 0.01), and REACH (SMD 2.12 (95% CI 0.76–3.48), *p* < 0.01). Aerobic exercise failed to significantly improve outcome. Sensitivity analysis revealed a similar overall treatment effect on skilled reaching (SMD 0.67 (95% CI 0.42–0.91), *p* < 0.01) with non-significant heterogeneity (*I*^2^ = 30%, *p* = 0.11). Interestingly, only ER and REACH subgroups remained significant in the sensitivity analysis (Fig. S3).

Funnel plot and Egger regression confirmed the presence of asymmetry in the dataset (*p* < 0.01); therefore, trim-and-fill analysis was completed (Fig. S4). Hypothetical data (*n* = 5) added by trim-and-fill analysis revealed missing negative and null data, suggesting reporting or publication bias. Random-effects meta-analysis of the trim-and-fill model (*n* = 29) produced a smaller treatment effect (SMD 0.59 (95% CI 0.32–0.87), *p* < 0.01).

To conceptualize efficacy beyond statistical testing, we completed a post hoc analysis using only interventions that reported the number of pellets retrieved in their respective skilled reaching tasks (Fig. S5). As before, rehabilitation improved skilled reaching success (MD 2.85 pellets retrieved (95% CI 1.97–3.74), *p* < 0.01; SMD 0.82 (95% CI 0.51–1.13), *p* < 0.01), which was comparable to the effect size observed in the full dataset.

### Efficacy of Rehabilitation on Spontaneous Use of the Impaired Forelimb

**Fig. 4:**
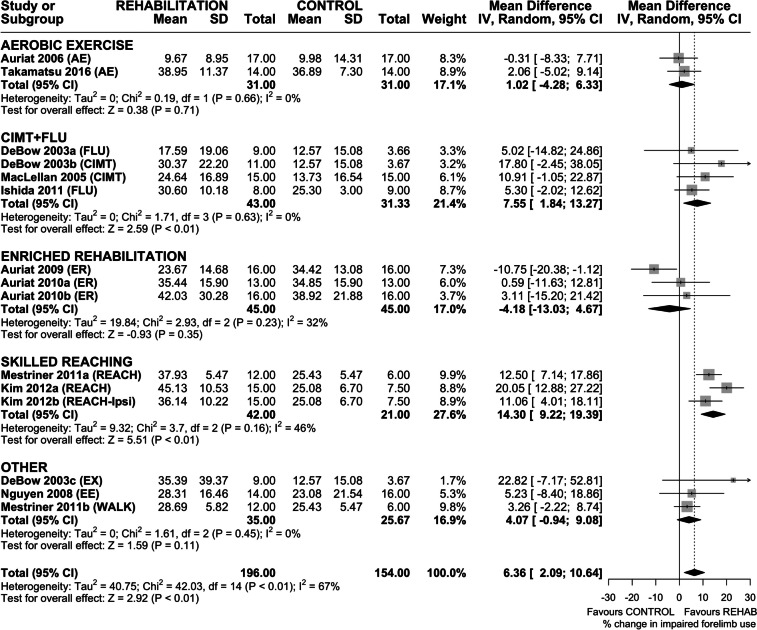
Forest plot of random-effects meta-analysis of post-ICH rehabilitation on spontaneous impaired forelimb use in the cylinder task. Overall, rehabilitation significantly increased impaired forelimb use (MD 6.36% improvement (95% CI 2.09–10.64), *p* < 0.01), but only REACH and CIMT + FLU were associated with significant treatment effects. Egger regression did not indicate the presence of asymmetry in the dataset. Effect sizes presented as mean difference (MD), percent change in impaired forelimb use, with 95% CI

Fourteen interventions assessed efficacy of rehabilitation on spontaneous impaired forelimb use in the cylinder task (Fig. 4). Overall, rehabilitation increased use of the impaired forelimb (MD 6.36% increase in impaired forelimb use (95% CI 2.09–10.64), *p* < 0.01); however heterogeneity was high (*I*^2^ = 67%, *p* < 0.01). Subgroup analysis by rehabilitation type found a significant effect of CIMT + FLU (MD 7.55% increase in impaired forelimb use (95% CI 1.84–13.27), *p* < 0.01) and REACH (MD 14.30% increase in impaired forelimb use (95% CI 9.22–19.39), *p* < 0.01). Neither AE nor ER significantly increased impaired forelimb use, and interestingly, ER-treated animals trended towards worse outcomes than non-treated controls. Sensitivity analysis revealed a similar overall treatment effect on impaired forelimb use (MD 7.49% increase in impaired forelimb use (95% CI 2.66–12.31), *p* < 0.01), again with high heterogeneity (*I*^2^ = 67%, *p* < 0.01). As before, CIMT + FLU and REACH improved spontaneous use of the impaired forelimb; ER did not, and again trended towards worse outcomes than non-treated controls (Fig. S6). Funnel plot and Egger regression did not reveal asymmetry in the dataset (*p* > 0.05); therefore, trim-and-fill analysis was not conducted (Fig. S7).

### Efficacy of Rehabilitation on Locomotor Function

**Fig. 5:**
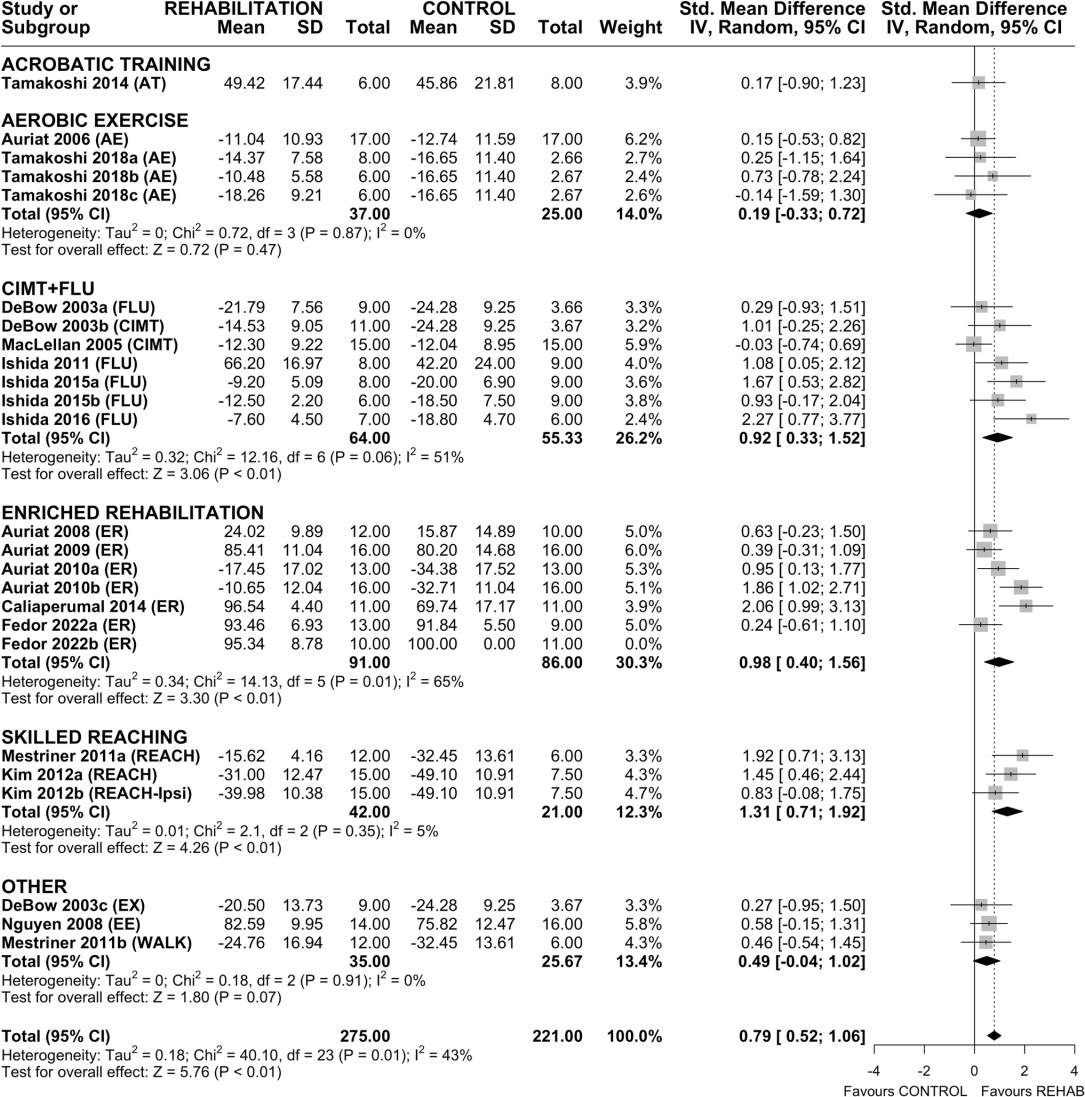
Forest plot of random-effects meta-analysis of post-ICH rehabilitation on locomotor function in the ladder walking task. Rehabilitation significantly improved locomotor function (SMD 0.79 (95% CI 0.52–1.06), *p* < 0.01). Egger regression did not indicate the presence of asymmetry in the dataset. Effect sizes presented as Hedge’s *G* standardized mean difference (SMD) with 95% CI

Thirty-one interventions assessed efficacy of rehabilitation on locomotor function in the ladder walking task (Fig. 5). One intervention was unweighted in our analysis (variance of zero [53]), while 5 others were excluded as we did not receive a response to our requests for clarification (1 did not report group sizes [62], in 4 we could not interpret the measure of central tendency or variability [43, 45, 47]). Overall, rehabilitation improved locomotor function (SMD 0.79, (95% CI 0.52–1.06), *p* < 0.01) and heterogeneity was moderate (*I*^2^ = 43%, *p* = 0.01). Subgroup analysis by rehabilitation type found a significant effect of CIMT + FLU (SMD 0.92 (95% CI 0.33–1.52), *p* < 0.01), ER (SMD 0.98 (95% CI 0.40–1.56), *p* < 0.01], and REACH (SMD 1.31 (95% CI 0.71–1.92), *p* < 0.01). Neither AE nor AT significantly improved locomotor function. Sensitivity analysis revealed a similar overall effect size for locomotor function (SMD 0.83 (95% CI 0.52–1.15), *p* < 0.01) and heterogeneity remained moderate (*I*^2^ = 45%, *p* = 0.03). As before, ER and REACH remained significant, whereas CIMT + FLU did not (Fig. S8). Funnel plot and accompanying Egger regression did not reveal asymmetry in the dataset (*p* > 0.05); therefore, trim-and-fill analysis was not conducted (Fig. S9).

### Efficacy of Rehabilitation on Beam Walking and Composite Neurobehavioral Test Scores

Eight interventions assessed efficacy of rehabilitation on locomotor function in the beam walking task, while 13 used a composite neurobehavioral test (e.g., NDS) to assess global function. Unfortunately, we were unable to analyze these endpoints for reasons related to both reporting and principles of analysis. Beam walking and composite neurobehavioral test batteries rely on assessors to make a subjective judgment on behavior or function; while this is not inherently a problem (assuming blinded assessors), the data for these results are non-parametric. As such, these tests should be analyzed using non-parametric methods with data reported as median ± IQR. Many articles made one or more of the following errors: use of parametric tests in analysis (i.e., ANOVA), reported mean ± SD or standard error (vs. median ± IQR), or failed to report a measure of variability. As such, it was inappropriate to analyze or draw conclusions from these data.

### Impact of the Timing of Rehabilitation Onset on Efficacy

**Fig. 6:**
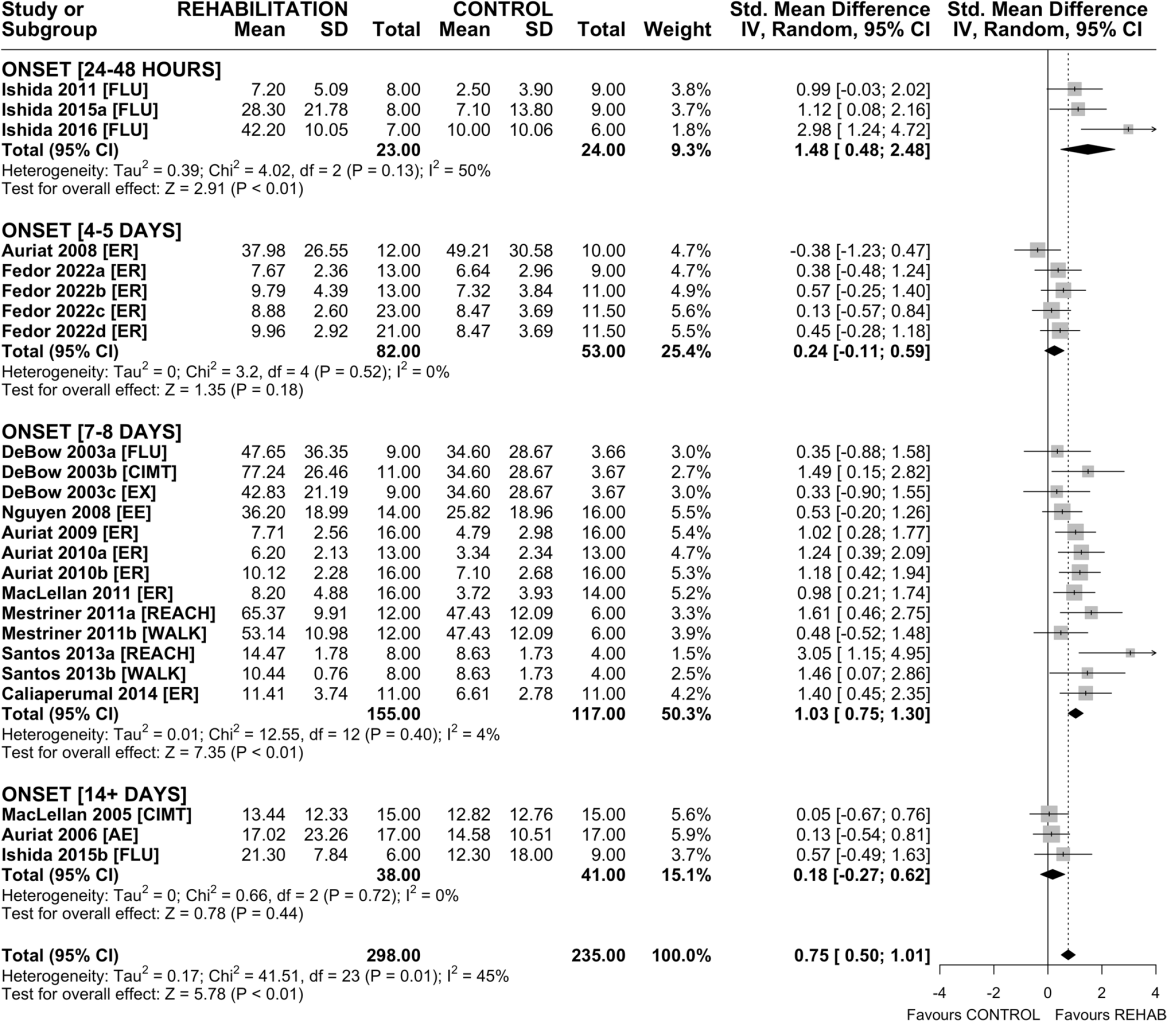
Forest plot of random-effects meta-analysis of skilled reaching performance grouped by timing of rehabilitation onset (days from ICH induction). Rehabilitation improved skilled reaching recovery with treatment onset of 24–48 h (SMD 1.48 (95% CI 0.48–2.48), *p* < 0.01) and 7–8 days (SMD 1.03 (95% CI 0.75–1.30), *p* < 0.01) whereas treatment initiated at 4–5 or ≥ 14 days failed to significantly improve skilled reaching. Effect sizes presented as Hedge’s *G* standardized mean difference (SMD) with 95% CI

**Fig. 7:**
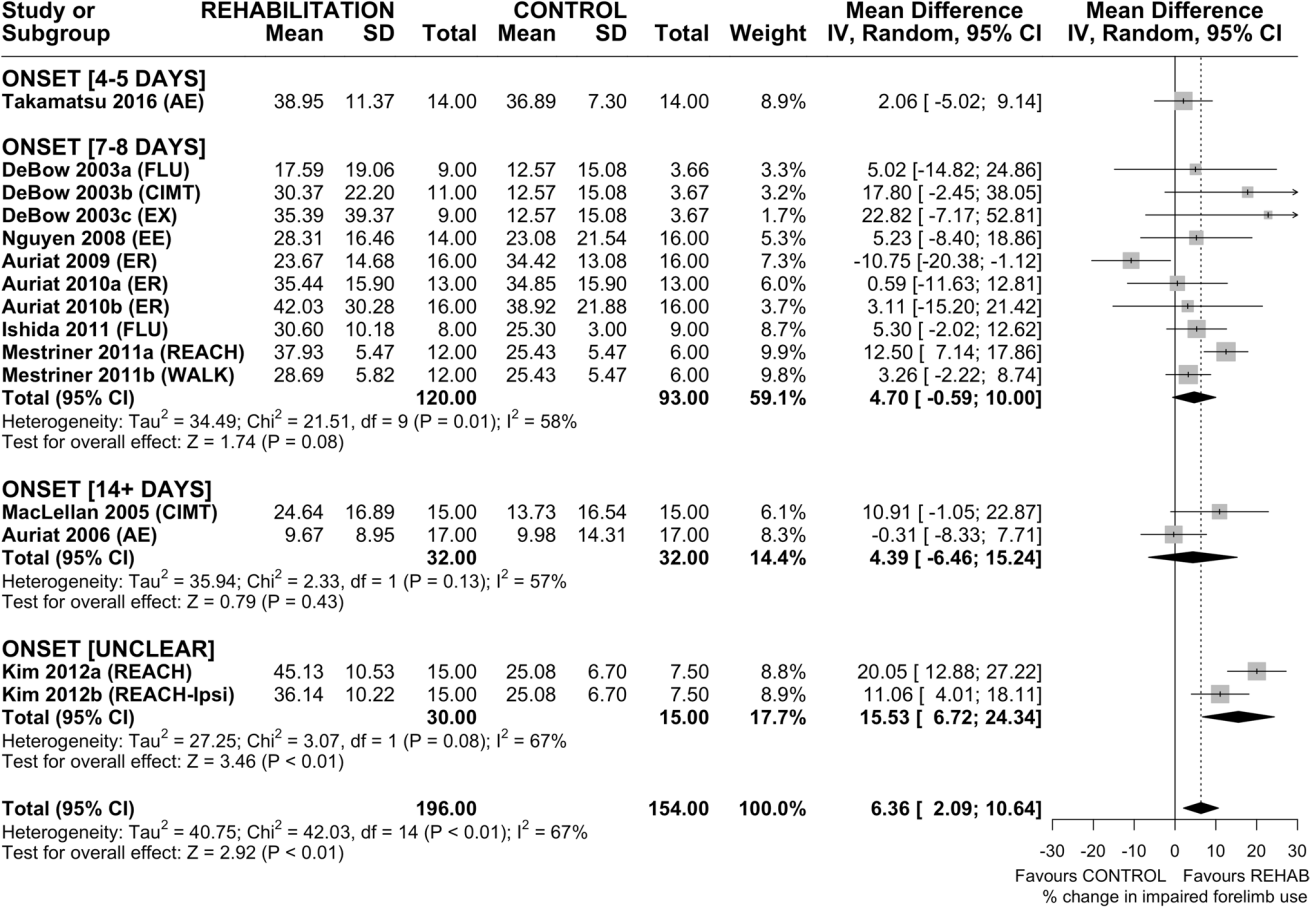
Forest plot of random effects meta-analysis of spontaneous impaired forelimb use grouped by timing of rehabilitation onset (days from ICH induction). Rehabilitation increased use of the impaired forelimb; however, this effect was predominately driven by two interventions with unclear treatment onset (Kim 2012a, b). Effect sizes presented as mean difference (MD), percent change in impaired forelimb use, with 95% CI

**Fig. 8:**
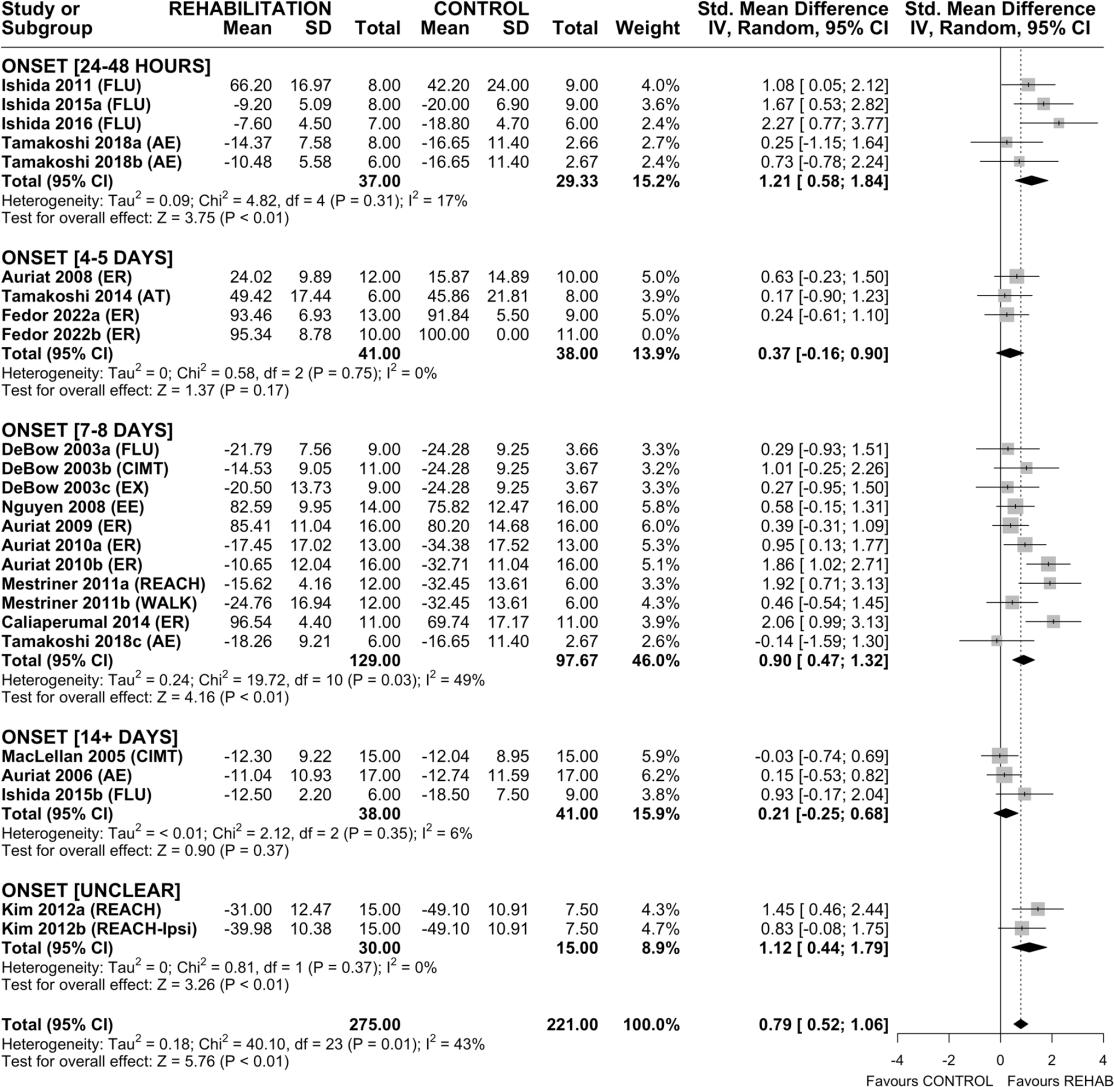
Forest plot of random-effects meta-analysis of recovery of locomotor function grouped by timing of rehabilitation onset (days from ICH induction). Rehabilitation improved locomotor function with treatment onset of 24–48 h (SMD 1.21 (95% CI 0.58–1.84), *p* < 0.01) and 7–8 days (SMD 0.90 (95% CI 0.47–1.32), *p* < 0.01); however, treatment initiated at 4–5 or ≥ 14 days failed to significantly improve locomotor function. Effect sizes presented as Hedge’s *G* standardized mean difference (SMD) with 95% CI

Based on intervention characteristics, timing of treatment onset grouped into 5 categories: 24–48 h, 4–5 days, 7–8 days, ≥ 14 days, and UNCLEAR. Rehabilitation improved skilled reaching recovery with treatment onset 24–48 h (SMD 1.48 (95% CI 0.48–2.48), *p* < 0.01) and 7–8 days (SMD 1.03 (95% CI 0.75–1.30), *p* < 0.01) after ICH; however, treatment initiated at 4–5 or ≥ 14 days failed to significantly improve skilled reaching (Fig. 6). The impact of treatment onset on improvement in spontaneous impaired forelimb use is unknown. While rehabilitation on the whole increased the use of the impaired forelimb, this effect was only significant in the UNCLEAR onset group (Fig. 7). Rehabilitation also improved locomotor function in the ladder walking task with treatment onset at 24–28 h (SMD 1.21 (95% CI 0.58–1.84), *p* < 0.01) and 7–8 days (SMD 0.90 (95% CI 0.47–1.32), *p* < 0.01) after ICH. Again, treatment initiated at 4–5 days or ≥ 14 days failed to significantly improve locomotor function (Fig. 8). Treatment onset within 24–48 h or 7–8 days after ICH appear to be most efficacious.

### Impact of Stroke Severity on Efficacy

To assess the impact of stroke severity on treatment efficacy, subgroups were identified using the mean lesion volume reported in the comparator group (untreated control). Severity was defined as mild (≤ 30 mm^3^), moderate (31–60 mm^3^), severe (≥ 61 mm^3^), and UNCLEAR [68, 69]. Rehabilitation improved skilled reaching in animals with mild (SMD 1.01 (95% CI 0.54–1.48), *p* < 0.01) and moderate (SMD 0.54 (95% CI 0.14–0.94), *p* < 0.01) but not severe ICH (Fig. S10). Rehabilitation did not improve impaired forelimb use when lesion severity was known (Fig. S11). Rehabilitation improved locomotor function in animals with mild (SMD 1.45 (95% CI 0.94–1.95), *p* < 0.01) and moderate (SMD 0.63 (95% CI 0.25–1.01), *p* < 0.01) but not severe ICH (Fig. S12). Consistent with clinical data, the most severe strokes were associated with limited treatment efficacy.

### Impact of Rehabilitation Dose by Treatment Type

Owing to substantial heterogeneity among rehabilitation interventions, impact of dose was assessed by treatment type. Analysis was only conducted if ≥ 3 intervention groups and ≥ 2 doses were present for an endpoint. Subgroups for treatment dose were identified by natural differences observed within each dataset.

CIMT + FLU was divided into three treatment doses: FLU (56 h), CIMT (FLU 56 h + EX 7 h), and FLU (168 h) (Fig. S13). While CIMT + FLU significantly improved skilled reaching, only FLU (168 h) was significantly associated with improved recovery (SMD 1.21 (95% CI 0.42–2.00), *p* < 0.01). CIMT + FLU improved spontaneous use of the impaired forelimb; however, only CIMT had a significant treatment effect (MD 12.69% (95% CI 2.39–22.99), *p* = 0.02). CIMT + FLU significantly improved locomotor function; similar to skilled reaching, only FLU (168 h) significantly improved recovery (SMD 1.37 (95% CI 0.79–1.95), *p* < 0.01).

Aerobic exercise was divided into two doses: 0–2500 m and 2501–5000 m (Fig. S14). Ladder walking was the only endpoint with ≥ 3 intervention groups and ≥ 2 treatment doses; however, AE did not improve locomotor recovery in the ladder walking task.

Enriched rehabilitation was divided into four doses based on time in EE and REACH: EE (50–100 h) + REACH (9–10 h), EE (100–150 h) + REACH (10 h), EE (100–150 h) + REACH (20 h), and EE (600 h) + EX (1.5 h) (Fig. S15). Enriched rehabilitation as a whole significantly improved skilled reaching; however, EE (100–150 h) + REACH (10 h) was the only protocol to confer significant benefit (SMD 1.14 (95% CI 0.78–1.50), *p* < 0.01). Similarly, ER significantly improved locomotor function in the ladder walking task, and only EE (100–150 h) + REACH (10 h) conferred significant benefit (SMD 1.26 (95% CI 0.48–2.04), *p* < 0.01).

## Discussion

Rehabilitation improved motor recovery in skilled reaching, spontaneous impaired forelimb use, and locomotor function. Unsurprisingly, there was substantial variation in the quality of reporting and risk of bias among reviewed articles. Both CIMT + FLU and REACH improved function across all endpoints, whereas ER only improved skilled reaching and locomotor function, and AE failed to improve recovery in any endpoint. Treatment dose did not influence recovery equally across rehabilitation types, and greater treatment dose did not consistently improve recovery. Treatments initiated 24–48 h and 7–8 days after ICH improved skilled reaching and ladder walking, whereas treatments initiated 4–5 days or ≥ 14 days after ICH did not facilitate recovery. Animals with smaller lesions (≤ 30 mm^3^, ~ 3.7% hemisphere volume) showed the greatest recovery in skilled reaching and locomotor function, whereas those with moderate lesions recovered to a lesser extent. We found limited treatment efficacy in animals that had severe ICH (≥ 61 mm^3^, ~ 7.5% hemisphere volume), and this was true across all functional domains. These findings are consistent with clinical data, and represent a translationally relevant range in injury relative to the average ICH size in patients (~ 27 cm^3^, or ~ 4.5% hemisphere volume) [69, 70]. However, this encompasses a wider range of injury than the mild and often narrow ranges reported in recent clinical trials of mobilization [71] and rehabilitation [72] after hemorrhagic stroke (1.1–1.6% and ~ 2% hemisphere volume, respectively).

Only CIMT + FLU and REACH treatments reliably improved recovery across all three functional domains. Treatment effects were greatest in the REACH group across all endpoints, followed by CIMT + FLU, suggesting that functional gains transferred to non-trained skills. Interestingly, AE failed to improve recovery after ICH. While it is unclear why, perhaps these interventions used very severe ICH; however, only one reported severity (91 mm^3^) [38]. Our findings differ from those arising from a meta-analysis of preclinical rehabilitation after ischemia, where rehabilitation improved running ability but not impaired forelimb function [26]. Additionally, forced running (AE) was effective in improving motor recovery after ischemia but CIMT was not [26], suggesting subtype-specific rehabilitative efficacy. Owing to the small number of interventions included in each subgroup, it is unclear what underlies these differences. Factors such as injury type (ischemia vs. ICH), location (cortical vs. subcortical), stroke size (mild vs. severe), treatment type and intensity, and timing of intervention may play a role.

The impact of treatment dose varied by treatment type, with greater dosages not consistently improving efficacy, suggesting a non-linear relationship between dose and recovery. In our CIMT + FLU analysis, only high dose FLU (168 h) improved skilled reaching and ladder walking, whereas only CIMT (FLU 56 h + EX 7 h) improved impaired forelimb use. In contrast, only moderate dose ER (EE 100–150 h + REACH 10 h) improved skilled reaching and ladder walking. Complicating our interpretation of dose, outside of AE interventions, few articles reported sufficient detail to assess total treatment dose; for example, only one article using REACH or ER reported the number of repetitions completed [53]. Consequently, and similar to most clinical trials, dose was assessed as time in treatment (CIMT + FLU, ER, REACH), which may not reflect the true extent of participation or whether intensity varied among interventions, thereby impacting efficacy [73]. Given the importance of dosage, and the limited and somewhat confusing findings here, it is clear that additional dose–response work is needed.

Rehabilitation initiated 24–48 h or 7–8 days after ICH was most beneficial, yet why treatment initiated 4–5 days after ICH did not provide benefit is unclear. Further assessment of interventions delivered ≤ 5 days after ICH identified FLU initiated 24 h after a small capsular hemorrhage (7–8 mm^3^) as the only intervention to provide significant functional benefit [56–58]. Treatments initiated at 48 h (AE, striatal ICH, severity unknown) [41], 4 days (AE, striatal ICH, ~ 60% striatal damage; AT, striatal ICH, severity unknown) [15, 40], and 5 days (ER, moderate striatal ICH, 38–40 mm^3^) [48, 53] did not provide benefit. Furthermore, of the interventions excluded from locomotor function analysis, 4 used an onset ≤ 48 h after ICH and were interpreted to suggest mixed effects of early AE intervention on both behavior and inflammation [43, 45, 47]. While earlier intervention in the subacute versus chronic phase of recovery is generally linked with greater benefits, clinical investigations into the safety and utility of interventions in the hyper-acute (0–24 h) and acute (1–7 days) phases of recovery [74] have yielded mixed results [71, 75–77]. Notably, the AVERT trial found that frequent, high dose, early out of bed mobilization was associated with decreased odds of favorable outcome at 3 months post-stroke [75], and that increased intensity (i.e., greater time out of bed), but not increased frequency of mobilization was associated with less favorable outcomes [76]. Similarly, studies in experimental models of brain injury have demonstrated that early and intense rehabilitation can exacerbate injury and worsen functional outcomes [21, 22]. Based on our findings, FLU (restraint, no training, standard laboratory housing) initiated 24 h after capsular hemorrhage may be beneficial; however, further exploration is required. One might hypothesize that FLU was of lower intensity and less stressful than AE and ER, with the latter negatively impacting recovery at sensitive times (e.g., nearing the peak of edema and secondary cell death after ICH) [78–80]. It becomes plausible then that intervention induced stress responses interact with endogenous injury and repair processes in a complex manner, such as exacerbating inflammation and/or supressing mechanisms thought to mediate recovery after ICH (e.g., activation of M2-type microglia and hematoma clearance), and that this may vary by lesion location or severity.

Study quality and risk of bias assessments showed pervasive reporting and methodological issues and potential publication bias among the reviewed articles. Common reporting errors (Table 5) increase the risk of bias, while incomplete and unclear reporting impairs our ability to draw conclusions about data validity and generalizability. Furthermore, small sample sizes with low statistical power often overinflate effect sizes, issues thoroughly discussed elsewhere [81–83]. We found a considerable range in group sizes used for behavioral analysis (*n* = 5 to 23) with analyzed group sizes often much smaller than initially reported, particularly in long-term survival and time course studies. Relatedly, the role of laboratory housing conditions on stroke recovery and treatment efficacy was often overlooked. A recent meta-analysis found conventional laboratory housing (vs. enriched housing) significantly compromises rodent health and likely increases severity of several diseases [84]. As conventional and solo housing are frequently used in preclinical rehabilitation for control conditions, many studies may have exaggerated effect sizes due to housing related worsening of health status in untreated (impoverished) controls relative to human control groups that still receive conventional therapies. Together, these quality issues may lead to widespread overestimation of effect sizes, a trend we observed in our skilled reaching analysis (Fig. S16).

No single experimental model or animal population perfectly replicates the complexity of the human brain [85], the pathological features of spontaneous ICH [79], or the timing of injury and recovery processes [18]. Thus, diversity in models, settings, and endpoints is recommended. Unfortunately, all articles in our review modeled sub-cortical ICH in rodents and 29/30 used the collagenase model of ICH, raising concerns about translation (e.g., to other injury locations or populations). Many of the interventions we reviewed lacked clinical relevance. For example, running > 1 km within the first day after ICH is unlikely to be used in clinical settings [45, 47]. Similarly, completing thousands of task repetitions during a 1–2 week training period is unlikely to be achieved in clinical practice [53]. Although some patients may achieve > 200 repetitions/day, the average number of task repetitions completed in upper limb clinical rehabilitation trials is 23–32 repetitions/session, and decreases as impairment increases [86]. Despite the clinical importance, only ~ 1/3 of articles systematically compared factors such as treatment onset [41, 43, 57], type [42, 44, 54, 59–61], or dose [41, 53, 62, 66]. Mixed results were often reported, underscoring the need for additional confirmatory-type studies in ICH that systematically manipulate and directly compare the impact of treatment parameters. Although useful, meta-analysis will never replace the need for high-quality, original research. At this time, we think it prudent to interpret our findings as strongly supporting the need for additional experimentation specifically varying intervention time or dose while also considering potential interactions with mechanisms of injury and repair (e.g., inflammatory responses). Should such studies confirm complex intervention-delay or dosage effects, then clinical studies will have to evaluate such hypotheses and determine a way to optimize intervention timing (and dosage), such as with biomarkers.

Preclinical interventions were typically characterized by a “one-size-fits-all” approach, which fails to replicate the individual and impairment specific, goal-oriented approach used by clinical rehabilitation professionals. Although skill transference may be expected from some interventions, many studies often selected endpoints that were unlikely to be improved by their chosen therapy without task specific training (e.g., using skilled reaching to measure AE efficacy) or only assessed gross impairment (e.g., NDS). Many of these endpoints fail to distinguish true recovery from compensation, a limitation relevant to our findings. Here, we refer to improvement in function as recovery of function; however, we cannot rule out that treatment effects could be a combination of recovery and compensation, or compensation alone. Although the statistical effects observed in this meta-analysis are considered large, they do not directly show how much rehabilitation improves functional recovery. Clinical trials frequently report treatment effects relative to the minimum clinically importance difference (MCID) [87]; however, few preclinical studies report an equivalent to the MCID or conceptualize efficacy beyond statistical testing, making interpretation of functional effect sizes challenging. In our review, only one article proposed an MCID-like threshold [53], arguing that a 3-pellet increase in reaching success (i.e., 1 level in the 7 level, 21-pellet staircase task) represents a meaningful difference in function. Thus, we analyzed the subset of interventions that reported the number of pellets retrieved in skilled reaching assessment. Rehabilitation improved reaching success by an average of 2.85 pellets but failed to exceed the 3-pellet threshold; by this measure, the treatment effect is likely of limited functional impact.

Numerous resources and guidelines have been developed to improve the quality of stroke research and translational success [29, 88–94], yet for reasons unknown, adherence to these guidelines remains far from universal [67]. Preclinical successes will continue to fail to translate if we do not disrupt this norm. Table 6 provides a roadmap for improving quality and translational potential in preclinical rehabilitation research. With the goal of increasing transparency and replicability, we identify actions to be taken at each step of the scientific process, identify relevant resources, and outline how each action contributes to the goal of improving scientific and translational rigor. In the spirit of transparency, we encourage researchers to make raw data available as supplementary files upon publication or available in a discipline specific open data repository (for an example, see the Open Data Commons for Spinal Cord Injury [95]). Similarly, we strongly encourage journals to require authors to submit raw data as part of the peer review and publication process. We believe that incorporating these actions from the outset of experimental design provides a framework to reduce bias and improve methodological rigor and transparency in reporting. However, poor reporting quality is not the root of the problem—it is a consequence of a larger systemic issue. New trainees, established researchers, and peer reviewers alike must be provided access to adequate training and resources if we want to improve research quality and reproducibility. We encourage research groups to include formal training on best practices in research and statistics when onboarding new members. Familiarization with best practice guidelines (i.e., ARRIVE [89, 92, 93], RIGOR [90]), how compliance to these guidelines is assessed (i.e., CAMARADES [31], SYRCLE [32]), and what these guidelines look like in practice are equally important skills to preclinical researchers as learning animal husbandry or basic surgical techniques.

**Table 6:** Roadmap to improving the quality of preclinical rehabilitation research

Element	Action	Goal
Scientific rigor	Use the RIGOR [90], ARRIVE [89, 93], SYRCLE [32], and CAMARADES [31] guidelines and checklists to inform decision making during experimental design. Consult the CONSIGN tool [94] to ensure selection of appropriate control groups (Phase: experimental planning)	Improve methodological rigor, reduce risk of bias through a priori study design
	Implement recommendations from RIGOR and ARRIVE during data collection—this includes (but is not limited to): use of power calculations to determine group size, collecting baseline data for ALL experimental groups, blinded induction of ICH or randomizing animals to treatment group AFTER induction, blinding researchers to treatment identity for assessment and analysis (particularly for subjective measures) (Phase: data collection)	Improve quality of data collected, reduce risk of bias introduced by researchers
	Analyze data using appropriate statistical methods (i.e., parametric tests for parametric data, non-parametric tests for non-parametric data, ensure data does not violate test assumptions), analyze and present appropriate summary statistics (e.g., mean ± SD or 95% CI for continuous data, median ± IQR for ordinal data), report exact summary data values and error terms, and use scatterplots to represent data whenever possible (Phase: data analysis)	Improve statistical reporting and interpretation of treatment effects
	Report methods and data as outlined by the RIGOR and ARRIVE guidelines—this includes (but is not limited to): explicit reporting of total *N*, inclusion and exclusion criteria, mortality and exclusions (with group identity; if none this should also be stated), *n*/group analyzed for EACH endpoint, types and timing of assessments used etc (Phase: manuscript preparation)	Improve transparency and reproducibility of methods and findings
	Review final manuscript for compliance with RIGOR, ARRIVE, SYRLCE, and CAMARADES guidelines and checklists; address any deficiencies prior to submission for peer review (Phase: peer review and publication)	Improve reporting quality
	Make raw data and relevant analysis code available via supplemental data files or data repository (Phase: peer review and publication)	Improve data sharing and research transparency
Translational rigor	Select translationally relevant endpoints and interventions (i.e., assessment type, timing, and dosage that mimic clinical elements) and include functional endpoints in ALL studies of rehabilitation. The goal of rehabilitation is to improve function and independence—endpoints used in preclinical work must assess impairments that are similar to those observed clinically (Phase: experimental planning)	Ensure preclinical treatments assess endpoints relevant to human disease/disorder
	Conduct multiple functional assessments (baseline, post-stroke and pre-treatment, and follow up) and prioritize long-term functional assessment (> 28 days) in study design. Whenever possible, ALL treated subjects should complete ALL functional endpoints to prevent loss of statistical power (Phase: experimental planning, data collection)	Ensure treatment effects are not due to baseline differences; provide insight into stability of treatment effects
	Include a histological assessment of stroke severity (i.e., hematoma or lesion volume) and report total volume of injury (i.e., not injury in a single slice or small region). Use comorbidities relevant to ICH (e.g., hypertension, old age, diabetes) (Phase: experimental planning, data collection, data analysis, manuscript preparation)	Provide context to interpret what population the treatment may benefit (or harm)
Rehabilitation dosage	Define and describe the following treatment parameters: type, timing (onset, hours/days from stroke), treatment period (first day of treatment—last day of treatment), duration of single treatment session (mins/hours/days), frequency of treatment sessions (Phase: experimental planning, data analysis, manuscript preparation)	Improve reporting quality of treatment protocols; increase replicability
	Operationally define and report treatment intensity (e.g., repetitions, m/min, time in restraint); calculate and report total treatment dosage (i.e., time in treatment, total repetitions, distance run, etc.) (Phase: experimental planning, data collection, data analysis, manuscript preparation)	Provide insight into how intensity may impact recovery
	Adopt a standardized approach to reporting timing of intervention and assessments: stroke = day 0 should be used as a reference point (Phase: experimental planning, data analysis, manuscript preparation)	Standardize how data is discussed in the context of time since stroke
	Include experimental timeline figure indicating the timing for all periods of training, treatment, and assessment (Phase: manuscript preparation)	Clarify experimental design; improve replicability

## Conclusion

Our systematic review and meta-analysis show that rehabilitation improves skilled reaching, spontaneous impaired forelimb use, and locomotor function after ICH. CIMT + FLU and REACH were the only therapies to improve motor recovery across all three domains, whereas ER improved skilled reaching and locomotor function, and AE did not provide benefit in any domain. Acknowledging the limited quality and scope of articles included in our analysis, these findings provide strong evidence for a statistically significant effect of rehabilitation after ICH, but one of unclear functional meaning. While earlier intervention was generally better than delayed intervention (i.e., onset at 7–8 days versus ≥ 14 days), efficacy of rehabilitation delivered < 7 days after ICH is unclear. In alignment with clinical findings, rehabilitation was most effective following mild-moderate ICH, but of limited benefit after severe ICH. As others have called for, our analysis of key issues in scientific rigor and translational relevance highlights the need for continuing improvements in ICH rehabilitation research, and a clear need for additional work on dosage, timing, and other parameters. Without these improvements, future studies risk using rehabilitative interventions with limited functional efficacy or clinical relevance, potentially wasting limited financial resources and time pursuing lines of inquiry propped up by poor data. As we move into the era of precision medicine, identifying characteristics that pinpoint who best benefits from a treatment and what factors impact efficacy is essential to increasing translational success, optimizing rehabilitation, and ultimately, improving patient outcomes.

## Supplementary Information

Below is the link to the electronic supplementary material.

Supplementary file1 (PDF 10592 kb)

## Data Availability

Data extracted from published records and included in the meta-analysis can be found in the manuscript figures and supplemental information (figures and tables). For inquiries related to the data extraction process, code, or analyses please contact BF.
