# Supplementary material for: Motor Rehabilitation Provides Modest Functional Benefits After Intracerebral Hemorrhage: a Systematic Review and Meta-Analysis of Translational Rehabilitation Studies
Source: Transl Stroke Res. 2023 Nov 20;16(2):484–511. doi: 10.1007/s12975-023-01205-w (PMC11976355; doi:10.1007/s12975-023-01205-w)
Supplement: Supplementary file 1 — Supplementary file1 (PDF 10592 kb) [file 12975_2023_1205_MOESM1_ESM.pdf]

Supplemental Information

Motor rehabilitation provides modest functional benefits after intracerebral hemorrhage: a systematic review and meta-analysis of translational rehabilitation studies

Britt A. Fedor, Noam H. Sander, Maxwell MacLaren, Lane J. Liddle, Crystal L. MacLellan, Frederick Colbourne

in *Translational Stroke Research (2023)*

Correspondence:
Britt Fedor
P217 Biological Sciences Building, Department of Psychology, University of Alberta Edmonton, Alberta, Canada, T6G 2E9
E-mail: bfedor@ualberta.ca

Table S1. Experimental Design Characteristics

**Article ID**	**Experimental Design**					**Behavioural Endpoints**				
**First Author Year [Ref]**	**Species, Strain [Sex]**	**ICH Model**	**Survival**	**Stroke Size Assessment**	**Rehabilitation**	**Forelimb Function [Test]**	**Locomotor Function [Test]**	**Neuro-behavioural Battery**	**Other [Test]**	**Latest Assessment**
DeBow 2003a [1]	Rat, Sprague-Dawley [M]	COL	60 days	Lesion volume	FLU	Skilled reaching [Montoya staircase], Spontaneous impaired forelimb use [cylinder]	Walking [horizontal ladder]		Elevated body swing test	60 days
DeBow 2003b [1]	Rat, Sprague-Dawley [M]	COL	60 days	Lesion volume	CIMT	Skilled reaching [Montoya staircase], Spontaneous impaired forelimb use [cylinder]	Walking [horizontal ladder]			60 days
DeBow 2003c [1]	Rat, Sprague-Dawley [M]	COL	60 days	Lesion volume	EX	Skilled reaching [Montoya staircase], Spontaneous impaired forelimb use [cylinder]	Walking [horizontal ladder]			60 days
MacLellan 2005 [2]	Rat, Sprague-Dawley [M]	COL	60 days	Lesion volume	CIMT	Skilled reaching [Montoya staircase], Spontaneous impaired forelimb use [cylinder]	Walking [horizontal ladder]			56 days
Auriat 2006 [3]	Rat, Long Evans [M]	COL	49 days	Lesion volume	AE	Skilled reaching [Montoya staircase], Spontaneous impaired forelimb use [cylinder]	Walking [horizontal ladder]			46 days
Auriat 2008 [4]	Rat, Sprague-Dawley [M]	COL	30 days	Lesion volume	ER	Skilled reaching [Montoya staircase, tray task], Spontaneous impaired forelimb use [cylinder]	Walking [horizontal ladder, elevated beam]	NDS		28 days

Article ID	Experimental Design					Behavioural Endpoints				
**First Author Year [Ref]**	**Species, Strain [Sex]**	**ICH Model**	**Survival**	**Stroke Size Assessment**	**Rehabilitation**	**Forelimb Function [Test]**	**Locomotor Function [Test]**	**Neuro-behavioural Battery**	**Other [Test]**	**Latest Assessment**
Nguyen 2008 [5]	Rat, Sprague-Dawley [F]	COL	56 days	Lesion volume	EE	Skilled reaching [tray task], Spontaneous impaired forelimb use [cylinder]	Walking [horizontal ladder, elevated beam]			56 days
Auriat 2009 [6]	Rat, Sprague-Dawley [M]	COL	46 days	Lesion volume	ER	Skilled reaching [Montoya staircase], Spontaneous impaired forelimb use [cylinder]	Walking [horizontal ladder]			46 days
Auriat 2010a [7]	Rat, Sprague-Dawley [M]	COL	39 days	Not assessed	ER	Skilled reaching [Montoya staircase], Spontaneous impaired forelimb use [cylinder]	Walking [horizontal ladder]			39 days
Auriat 2010b [7]	Rat, Sprague-Dawley [M]	COL	32 days	Lesion volume	ER	Skilled reaching [Montoya staircase, tray task], Spontaneous impaired forelimb use [cylinder]	Walking [horizontal ladder]			32 days
Takamatsu 2010 [8]	Rat, Wistar [M]	COL	15 days	Lesion volume	AE			MDS		15 days
Ishida 2011 [9]	Rat, Wistar [M]	COL	37 days	Lesion volume	FLU	Skilled reaching [single pellet task], Spontaneous impaired forelimb use [cylinder]	Walking [horizontal ladder]	MDS	Sensorimotor [forelimb contact placing response], Kinematic analysis [forelimb]	28 days

Article ID	Experimental Design					Behavioural Endpoints				
**First Author Year [Ref]**	**Species, Strain [Sex]**	**ICH Model**	**Survival**	**Stroke Size Assessment**	**Rehabilitation**	**Forelimb Function [Test]**	**Locomotor Function [Test]**	**Neuro-behavioural Battery**	**Other [Test]**	**Latest Assessment**
MacLellan 2011 [10]	Rat, Sprague-Dawley [M]	AWB	49 days	Lesion volume	ER	Skilled reaching [single pellet task]				46 days
Mestriner 2011a [11]	Rat, Wistar [M]	COL	33-34 days	Lesion volume	REACH	Skilled reaching [Montoya staircase], Spontaneous impaired forelimb use [cylinder]	Walking [horizontal ladder]			33-34 days
Mestriner 2011b [11]	Rat, Wistar [M]	COL	33-34 days	Lesion volume	WALK	Skilled reaching [Montoya staircase], Spontaneous impaired forelimb use [cylinder]	Walking [horizontal ladder]			33-34 days
Kim 2012a [12]	Rat, Sprague-Dawley [M]	COL	Unclear, imprecise timeline	Lesion volume	REACH	Spontaneous impaired forelimb use [cylinder]	Walking [horizontal ladder]			Unclear, imprecise timeline
Kim 2012b [12]	Rat, Sprague-Dawley [M]	COL	Unclear, imprecise timeline	Lesion volume	REACH-ipsi	Spontaneous impaired forelimb use [cylinder]	Walking [horizontal ladder]			Unclear, imprecise timeline
Santos 2013a [13]	Rat, Wistar [M]	COL	33-34 days	Lesion volume* (conducted in one tissue slice)	REACH	Skilled reaching [Montoya staircase], Spontaneous impaired forelimb use [cylinder]				33-34 days
Santos 2013b [13]	Rat, Wistar [M]	COL	33-34 days	Lesion volume* (conducted in one tissue slice)	WALK	Skilled reaching [Montoya staircase], Spontaneous impaired forelimb use [cylinder]				33-34 days

Article ID	Experimental Design					Behavioural Endpoints				
**First Author Year [Ref]**	**Species, Strain [Sex]**	**ICH Model**	**Survival**	**Stroke Size Assessment**	**Rehabilitation**	**Forelimb Function [Test]**	**Locomotor Function [Test]**	**Neuro-behavioural Battery**	**Other [Test]**	**Latest Assessment**
Caliaperumal 2014 [14]	Rat, Sprague-Dawley [M]	COL	32 days	Not assessed	ER	Skilled reaching [Montoya staircase]	Walking [horizontal ladder]			32 days
Tamakoshi 2014 [15]	Rat, Wistar [M]	COL	29 days	Lesion volume	AT		Walking [horizontal ladder, elevated beam]	MDS		28 days
Yong 2014a [16]	Rat, Sprague-Dawley [M]	COL	7 days	Not assessed	REACH		Walking [horizontal ladder]			Unclear, imprecise timeline
Yong 2014b [16]	Rat, Sprague-Dawley [M]	COL	28 days	Not assessed	REACH		Walking [horizontal ladder]			Unclear, imprecise timeline
Ishida 2015a [17]	Rat, Wistar [M]	COL	14 days	Lesion volume	FLU	Skilled reaching [single pellet task]	Walking [horizontal ladder]			12 days
Ishida 2015b [17]	Rat, Wistar [M]	COL	30 days	Lesion volume	FLU	Skilled reaching [single pellet task]	Walking [horizontal ladder]			28 days
Ishida 2016 [18]	Rat, Wistar [M]	COL	60 days	Not assessed	FLU	Skilled reaching [single pellet task]	Walking [horizontal ladder]	MDS		28 days

Article ID	Experimental Design					Behavioural Endpoints				
**First Author Year [Ref]**	**Species, Strain [Sex]**	**ICH Model**	**Survival**	**Stroke Size Assessment**	**Rehabilitation**	**Forelimb Function [Test]**	**Locomotor Function [Test]**	**Neuro-behavioural Battery**	**Other [Test]**	**Latest Assessment**
Takamatsu 2016 [19]	Rat, Wistar [M]	COL	15 days	Not assessed	AE	Spontaneous impaired forelimb use [cylinder]	Walking [elevated beam] *hindlimb	MDS		15 days
Tamakoshi 2016 [20]	Rat, Wistar [M]	COL	29 days	Not assessed	AT				Sensorimotor [forepaw grasping, modified forelimb placing test, postural instability]	28 days
Tamakoshi 2017 [21]	Rat, Wistar [M]	COL	29 days	Lesion volume* (assessed, not reported)	AT				Sensorimotor [modified forelimb placing test]	28 days
Tamakoshi 2018a [22]	Rat, Wistar [M]	COL	15 days	Unclear* (lesion volume mentioned, methods/data not reported)	AE		Walking [horizontal ladder]		Sensorimotor [forelimb contact placing response]	15 days
Tamakoshi 2018b [22]	Rat, Wistar [M]	COL	15 days	Unclear* (lesion volume mentioned, methods/data not reported)	AE		Walking [horizontal ladder]		Sensorimotor [forelimb contact placing response]	15 days

Article ID	Experimental Design					Behavioural Endpoints				
First Author Year [Ref]	Species, Strain [Sex]	ICH Model	Survival	Stroke Size Assessment	Rehabilitation	Forelimb Function [Test]	Locomotor Function [Test]	Neuro-behavioural Battery	Other [Test]	Latest Assessment
Tamakoshi 2018c [22]	Rat, Wistar [M]	COL	15 days	Unclear* (lesion volume mentioned, methods/data not reported)	AE		Walking [horizontal ladder]		Sensorimotor [forelimb contact placing response]	15 days
Sato 2020a [23]	Rat, Sprague-Dawley [M]	COL	28 days	Not assessed	AE		Walking [elevated beams - narrow, wide]	MDS		28 days
Sato 2020b [23]	Rat, Sprague-Dawley [M]	COL	28 days	Not assessed	AE		Walking [elevated beams - narrow, wide]	MDS		28 days
Tamaokshi 2020a [24]	Rat, Wistar [M]	COL	16 days	Lesion volume	AE		Walking [horizontal ladder]		Sensorimotor [forelimb contact placing response], Balance/ coordination [rotarod]	15 days
Tamaokshi 2020b [24]	Rat, Wistar [M]	COL	16 days	Lesion volume	AE		Walking [horizontal ladder]		Sensorimotor [forelimb contact placing response], Balance/ coordination [rotarod]	15 days
Xu 2020a [25]	Rat, Sprague-Dawley [NR]	COL	14 days	Not assessed	AE			mNSS		14 days

Article ID	Experimental Design					Behavioural Endpoints				
First Author Year [Ref]	Species, Strain [Sex]	ICH Model	Survival	Stroke Size Assessment	Rehabilitation	Forelimb Function [Test]	Locomotor Function [Test]	Neuro-behavioural Battery	Other [Test]	Latest Assessment
Xu 2020b [25]	Rat, Sprague-Dawley [NR]	COL	14 days	Not assessed	AE			mNSS		14 days
Tamakoshi 2021 [26]	Rat, Wistar [M]	COL	27 hours	Hematoma volume	AE		Walking [horizontal ladder]		Balance/ coordination [rotarod]	25 hours
Fedor 2022a [27]	Rat, Sprague-Dawley [M]	COL	14 days	Hematoma volume	ER	Skilled reaching [Montoya staircase]	Walking [horizontal ladder, elevated beam]			14 days
Fedor 2022b [27]	Rat, Sprague-Dawley [M]	COL	14 days	Hematoma volume	ER	Skilled reaching [Montoya staircase]	Walking [horizontal ladder, elevated beam]			14 days
Fedor 2022c [27]	Rat, Sprague-Dawley [M]	COL	60 days	Lesion volume	ER	Skilled reaching [Montoya staircase]				31 days
Fedor 2022d [27]	Rat, Sprague-Dawley [M]	COL	60 days	Lesion volume	ER	Skilled reaching [Montoya staircase]				31 days
Inoue 2022 [28]	Rat, Wistar [M]	COL	29 days	Lesion volume	AE				Sensorimotor [tape removal task]	28 days

Article ID	Experimental Design					Behavioural Endpoints				
**First Author Year [Ref]**	**Species, Strain [Sex]**	**ICH Model**	**Survival**	**Stroke Size Assessment**	**Rehabilitation**	**Forelimb Function [Test]**	**Locomotor Function [Test]**	**Neuro-behavioural Battery**	**Other [Test]**	**Latest Assessment**
Li 2022a [29]	Mouse, C57BL/6J [M]	COL	Unclear, imprecise timeline	Hematoma volume	SWIM			mNSS		14 days
Li 2022b [29]	Mouse, C57BL/6J [M]	COL	Unclear, imprecise timeline	Hematoma volume	SWIM			mNSS		14 days
Li 2022c [29]	Mouse, C57BL/6J [M]	COL	Unclear, imprecise timeline	Hematoma volume	SWIM			mNSS		7 days
Tamakoshi 2022 [30]	Rat, Wistar [M]	COL	8 days	Lesion volume	AE		Walking [horizontal ladder]		Balance/ coordination [rotarod]	7 days

AE, aerobic exercise; AT, acrobatic training; AWB, autologous whole blood; CIMT, constraint-induced movement therapy; COL, collagenase model; EE, environmental enrichment; ER, enriched rehabilitation; F, female; FLU, forced limb use; M, male; MDS, motor deficit score; mNSS modified neurological severity score; NR, not reported; NDS, neurological deficit score; REACH, skilled reach training; REACH-ipsi, skilled reach training in unimpaired forelimb; SWIM, swim training; WALK, walk training

Article	CAMARADES Checklist Item									
	1	2	3	4	5	6	7	8	9	10
DeBow 2003	+	+	+	–	+	×	×	×	+	×
MacLellan 2005	+	+	–	–	+	×	×	×	+	×
Auriat 2006	+	+	–	–	–	×	×	×	+	×
Auriat 2008	+	+	+	–	–	×	×	×	+	×
Nguyen 2008	+	+	+	+	+	×	+	×	+	×
Auriat 2009	+	+	+	+	+	×	×	×	+	×
Auriat 2010	+	+	+	–	–	×	×	×	+	×
Takamatsu 2010	+	–	+	–	–	×	×	×	+	×
Ishida 2011	+	+	+	–	–	×	×	×	+	×
MacLellan 2011	+	+	+	–	+	×	×	×	+	+
Mestriner 2011	+	+	×	–	+	–	×	×	+	×
Kim 2012	+	–	+	–	–	×	×	×	+	+
Santos 2013	+	+	×	–	–	–	×	×	+	×
Caliaperumal 2014	+	+	+	+	+	×	×	×	+	+
Tamakoshi 2014	+	–	–	+	–	×	×	×	+	×
Yong 2014	+	–	+	–	–	×	×	×	+	×
Ishida 2015	+	–	–	–	–	×	×	×	+	×
Ishida 2016	+	+	–	–	–	×	×	×	+	×
Takamatsu 2016	+	–	+	–	×	×	×	×	+	×
Tamakoshi 2016	+	–	+	–	–	×	×	×	+	×
Tamakoshi 2017	+	–	+	–	–	×	×	×	+	×
Tamakoshi 2018	+	–	+	–	–	×	×	×	+	×
Sato 2020	+	–	–	–	–	–	×	×	+	+
Tamakoshi 2020	+	–	+	–	–	×	×	×	+	+
Xu 2020	+	+	+	+	+	–	×	×	+	+
Tamakoshi 2021	+	–	+	–	+	×	×	×	+	+
Fedor 2022	+	+	+	+	+	×	×	+	+	+
Inoue 2022	+	–	–	–	–	–	×	×	+	+
Li 2022	+	–	+	–	–	×	×	×	+	+
Tamakoshi 2022	+	–	+	–	–	×	×	×	+	+

CAMARADES Checklist Item

1: Peer reviewed
2: Temperature control during ICH surgery
3: Reported random allocation to groups
4: Blinded ICH induction OR post-ICH randomization
5: Blinded outcome assessments
6: Used anaesthetic(s) without intrinsic neuroprotective effects
7: Included comorbidities relevant to ICH
8: Described sample size calculation
9: Statement on compliance with animal welfare regulations
10: Explicit conflict of interest statement

Rating

× No
– Unclear
+ Yes

Fig. S1 Individual article ratings for assessment of compliance with 10 item CAMARADES checklist. Articles were rated as yes, unclear, or no for their compliance on each item. A rating of unclear was given when reviewers deemed there was insufficient and/or inconsistent reporting of detail to accurately judge compliance with the checklist item. Article quality ranged considerably (scores of 2-8), with a median score of 4

	SYRCLE Risk of Bias Domain									
**Article**	**1**	**2**	**3**	**4**	**5**	**6**	**7**	**8**	**9**	**10**
DeBow 2003	Unclear	Unclear	Unclear	Unclear	Not rated	Low	Low	Low	Unclear	Low
MacLellan 2005	Unclear	Unclear	Unclear	Unclear	Not rated	Low	Low	Low	Unclear	Unclear
Auriat 2006	Unclear	Low	Unclear	Unclear	Not rated	Low	Unclear	Unclear	Unclear	Low
Auriat 2008	Unclear	Unclear	Unclear	High	Not rated	Low	Unclear	Low	Unclear	High
Nguyen 2008	Unclear	Low	Unclear	High	Not rated	Low	Low	Unclear	Unclear	High
Auriat 2009	Unclear	Low	Unclear	High	Not rated	Low	Low	Low	Unclear	High
Auriat 2010	Unclear	Low	Unclear	High	Not rated	Unclear	Unclear	High	Unclear	High
Takamatsu 2010	Unclear	Unclear	Unclear	Unclear	Not rated	Unclear	Unclear	Unclear	Unclear	Unclear
Ishida 2011	Unclear	Unclear	Unclear	Unclear	Not rated	Unclear	Unclear	Unclear	Unclear	High
MacLellan 2011	Unclear	Low	Unclear	High	Not rated	Unclear	Low	Unclear	Unclear	High
Mestriner 2011	High	Low	High	Unclear	Not rated	Low	Low	Unclear	Unclear	High
Kim 2012	Unclear	Unclear	Unclear	Unclear	Not rated	Unclear	Unclear	Unclear	High	Unclear
Santos 2013	High	Low	Unclear	Unclear	Not rated	Low	Unclear	Low	Unclear	High
Caliaperumal 2014	Unclear	Low	Unclear	High	Not rated	Low	Low	Low	Unclear	High
Tamakoshi 2014	Unclear	Unclear	Unclear	Unclear	Not rated	Unclear	Unclear	Unclear	High	High
Yong 2014	Unclear	Unclear	Unclear	Unclear	Not rated	Unclear	Unclear	Unclear	Unclear	Unclear
Ishida 2015	Unclear	Low	Unclear	Unclear	Not rated	Unclear	Unclear	High	Unclear	High
Ishida 2016	Unclear	Unclear	Unclear	Unclear	Not rated	Unclear	Unclear	Unclear	High	Unclear
Takamatsu 2016	Unclear	Unclear	Unclear	Unclear	Not rated	Unclear	High	High	Unclear	High
Tamakoshi 2016	Unclear	Unclear	Unclear	Unclear	Not rated	Unclear	Unclear	Unclear	High	High
Tamakoshi 2017	Unclear	Unclear	Unclear	Unclear	Not rated	Unclear	Unclear	High	High	High
Tamakoshi 2018	Unclear	High	Unclear	Unclear	Not rated	Unclear	Unclear	Unclear	High	Unclear
Sato 2020	Unclear	Unclear	Unclear	High	Not rated	Unclear	Unclear	High	Unclear	High
Tamakoshi 2020	Unclear	Unclear	Unclear	Unclear	Not rated	Unclear	Unclear	High	High	High
Xu 2020	Unclear	High	Unclear	Unclear	Not rated	Unclear	Unclear	Low	High	Unclear
Tamakoshi 2021	Unclear	Unclear	Unclear	Unclear	Not rated	Unclear	Low	Unclear	Unclear	High
Fedor 2022	Low	Low	Unclear	High	Not rated	Low	Low	Low	Unclear	High
Inoue 2022	Unclear	Unclear	Unclear	Unclear	Not rated	Low	Unclear	Low	Unclear	Unclear
Li 2022	Unclear	High	Unclear	Unclear	Not rated	Unclear	Unclear	High	High	High
Tamakoshi 2022	Unclear	Unclear	Unclear	Unclear	Not rated	Unclear	Unclear	High	High	High

SYRCLE Risk of Bias Domain

1: Sequence generation (Selection bias)
2: Baseline characteristics (Selection bias)
3: Allocation concealment (Selection bias)
4: Random housing (Performance bias)
5: Caregiver blinding (Performance bias)
6: Random outcome assessment (Detection bias)
7: Blinding (Detection bias)
8: Incomplete outcome assessment (Attrition bias)
9: Selective outcome reporting (Reporting bias)
10: Other sources of bias (Other)

Rating

High
Unclear
Low
Not rated

Fig. S2 Individual article ratings for SYRCLE Risk of Bias tool. Articles were rated for each domain as low-, unclear-, or high risk. Caregiver blinding (performance bias) was not rated, as it is near impossible for preclinical researchers to be blinded to rehabilitation delivery. A rating of unclear was given when reviewers deemed there was insufficient and/or inconsistent reporting of detail to accurately judge compliance with the signalling questions for a domain. Risk of bias was predominately unclear, as articles often lacked sufficient detail to determine how/if risk of bias was minimized

Study or Subgroup	REHABILITATION Mean	SD	Total	CONTROL Mean	SD	Total	Weight	Std. Mean Difference IV, Random, 95% CI
**CIMT+FLU**								
**DeBow 2003a (FLU)**	47.65	36.35	9.00	34.60	28.67	3.66	3.3%	0.35 [-0.88; 1.58]
**DeBow 2003b (CIMT)**	77.24	26.46	11.00	34.60	28.67	3.67	2.9%	1.49 [0.15; 2.82]
**MacLellan 2005 (CIMT)**	13.44	12.33	15.00	12.82	12.76	15.00	7.3%	0.05 [-0.67; 0.76]
**Ishida 2011 (FLU)**	7.20	5.09	8.00	2.50	3.90	9.00	4.4%	0.99 [-0.03; 2.02]
**Total (95% CI)**			**43.00**			**31.33**	**17.9%**	**0.59 [-0.04; 1.23]**
Heterogeneity: $Tau^2 = 0.15$; $Chi^2 = 4.57$, df = 3 (P = 0.21); $I^2 = 34\%$								
Test for overall effect: Z = 1.83 (P = 0.07)								
**ENRICHED REHABILITATION**								
**Auriat 2008 (ER)**	37.98	26.55	12.00	49.21	30.58	10.00	5.8%	-0.38 [-1.23; 0.47]
**Auriat 2009 (ER)**	7.71	2.56	16.00	4.79	2.98	16.00	7.0%	1.02 [0.28; 1.77]
**Auriat 2010a (ER)**	6.20	2.13	13.00	3.34	2.34	13.00	5.8%	1.24 [0.39; 2.09]
**Auriat 2010b (ER)**	10.12	2.28	16.00	7.10	2.68	16.00	6.8%	1.18 [0.42; 1.94]
**MacLellan 2011 (ER)**	8.20	4.88	16.00	3.72	3.93	14.00	6.7%	0.98 [0.21; 1.74]
**Caliaperumal 2014 (ER)**	11.41	3.74	11.00	6.61	2.78	11.00	4.9%	1.40 [0.45; 2.35]
**Fedor 2022a (ER)**	7.67	2.36	13.00	6.64	2.96	9.00	5.7%	0.38 [-0.48; 1.24]
**Fedor 2022b (ER)**	9.79	4.39	13.00	7.32	3.84	11.00	6.1%	0.57 [-0.25; 1.40]
**Fedor 2022c (ER)**	8.88	2.60	23.00	8.47	3.69	11.50	7.4%	0.13 [-0.57; 0.84]
**Fedor 2022d (ER)**	9.96	2.92	21.00	8.47	3.69	11.50	7.1%	0.45 [-0.28; 1.18]
**Total (95% CI)**			**154.00**			**123.00**	**63.3%**	**0.69 [0.35; 1.03]**
Heterogeneity: $Tau^2 = 0.13$; $Chi^2 = 16.15$, df = 9 (P = 0.06); $I^2 = 44\%$								
Test for overall effect: Z = 3.99 (P < 0.01)								
**SKILLED REACHING**								
**Mestriner 2011a (REACH)**	65.37	9.91	12.00	47.43	12.09	6.00	3.7%	1.61 [0.46; 2.75]
**OTHER**								
**DeBow 2003c (EX)**	42.83	21.19	9.00	34.60	28.67	3.67	3.3%	0.33 [-0.90; 1.55]
**Nguyen 2008 (EE)**	36.20	18.99	14.00	25.82	18.96	16.00	7.1%	0.53 [-0.20; 1.26]
**Mestriner 2011b (WALK)**	53.14	10.98	12.00	47.43	12.09	6.00	4.6%	0.48 [-0.52; 1.48]
**Total (95% CI)**			**35.00**			**25.67**	**15.0%**	**0.48 [-0.05; 1.01]**
Heterogeneity: $Tau^2 = 0$; $Chi^2 = 0.08$, df = 2 (P = 0.96); $I^2 = 0\%$								
Test for overall effect: Z = 1.77 (P = 0.08)								
**Total (95% CI)**			**244.00**			**186.00**	**100.0%**	**0.67 [0.42; 0.91]**
Heterogeneity: $Tau^2 = 0.08$; $Chi^2 = 24.25$, df = 17 (P = 0.11); $I^2 = 30\%$								
Test for overall effect: Z = 5.32 (P < 0.01)								

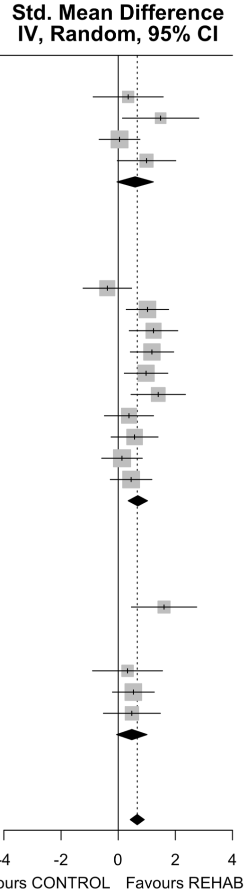

Fig. S3 Forest plot of the sensitivity analysis conducted to evaluate the impact of research quality on recovery of skilled reaching. Interventions from articles with a CAMARADES score of 0-3 were removed (n=6) and random-effects meta-analysis was conducted (n=18). We observed a similar overall treatment effect [SMD 0.67 (95% CI 0.42-0.91), p<0.01] to our original model and found removing low quality studies significantly reduced heterogeneity (I^2=30%, p=0.11). Effect sizes presented as Hedge's *G* standardized mean difference (SMD) with 95% CI

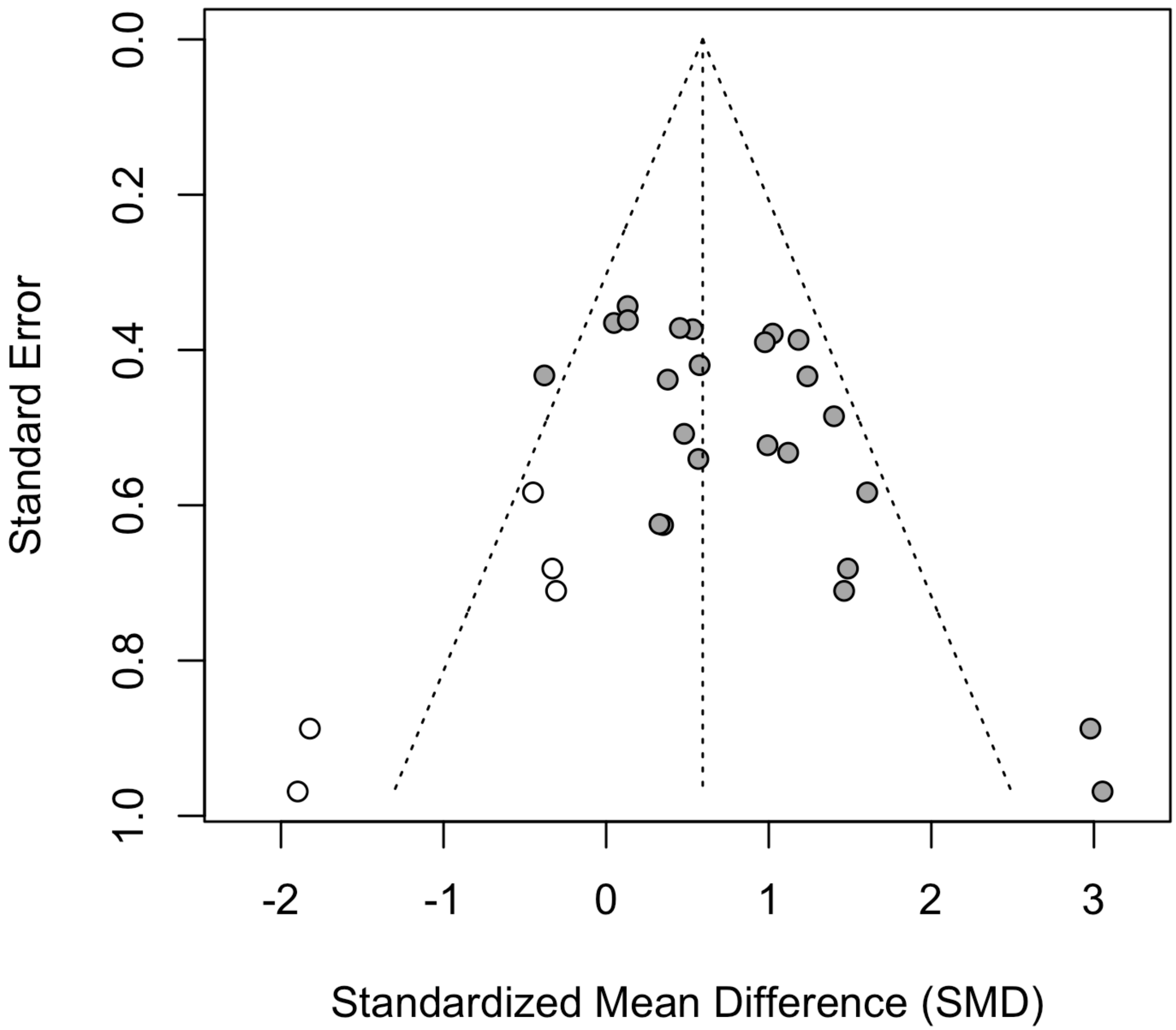

Fig. S4 Funnel plot of skilled reaching data (Fig.3). Egger regression indicated the presence of asymmetry in the dataset (p<0.01); therefore, trim-and-fill analysis was conducted. Filled circles represent real data (n=24), open circles represent hypothetical data added through trim-and-fill analysis (n=5). All additional data points were added in the bottom left quadrant, suggesting null or negative data may be absent in our original model, likely due to reporting and/or publication bias. Random-effects meta-analysis of the trim-and-fill model (n=29) produced a smaller treatment effect [SMD 0.59 (95% CI 0.32-0.87), p<0.01] than our original model [SMD 0.75 (95% CI 0.50-1.00)]. Effect sizes presented as Hedge's *G* standardized mean difference (SMD)

Study or Subgroup	REHABILITATION Mean	SD	Total	CONTROL Mean	SD	Total	Weight	Mean Difference IV, Random, 95% CI
**PELLETS RETRIEVED [MONTOYA STAIRCASE]**								
**Auriat 2006 (AE)**	17.02	23.26	17.00	14.58	10.51	17.00	0.5%	2.44 [-9.69; 14.57]
**Auriat 2009 (ER)**	7.71	2.56	16.00	4.79	2.98	16.00	10.3%	2.92 [1.00; 4.84]
**Auriat 2010a (ER)**	6.20	2.13	13.00	3.34	2.34	13.00	11.4%	2.86 [1.14; 4.58]
**Auriat 2010b (ER)**	10.12	2.28	16.00	7.10	2.68	16.00	11.4%	3.02 [1.30; 4.74]
**Santos 2013a (REACH)**	14.47	1.78	8.00	8.63	1.73	4.00	9.5%	5.84 [3.74; 7.94]
**Santos 2013b (WALK)**	10.44	0.76	8.00	8.63	1.73	4.00	11.1%	1.81 [0.03; 3.59]
**Caliaperumal 2014 (ER)**	11.41	3.74	11.00	6.61	2.78	11.00	6.8%	4.80 [2.05; 7.55]
**Fedor 2022a (ER)**	7.67	2.36	13.00	6.64	2.96	9.00	8.4%	1.03 [-1.29; 3.35]
**Fedor 2022b (ER)**	9.79	4.39	13.00	7.32	3.84	11.00	5.3%	2.47 [-0.82; 5.76]
**Fedor 2022c (ER)**	8.88	2.60	23.00	8.47	3.69	11.50	8.2%	0.41 [-1.97; 2.79]
**Fedor 2022d (ER)**	9.96	2.92	21.00	8.47	3.69	11.50	7.8%	1.49 [-0.98; 3.96]
**Total (95% CI)**			**159.00**			**124.00**	**90.9%**	**2.68 [1.73; 3.63]**
Heterogeneity: $Tau^2 = 1.11$; $Chi^2 = 18.51$, df = 10 (P = 0.05); $I^2 = 46\%$								
Test for overall effect: Z = 5.53 (P < 0.01)								
**PELLETS RETRIEVED [SINGLE PELLET TASK]**								
**Ishida 2011 (FLU)**	7.20	5.09	8.00	2.50	3.90	9.00	3.4%	4.70 [0.35; 9.05]
**MacLellan 2011 (ER)**	8.20	4.88	16.00	3.72	3.93	14.00	5.7%	4.48 [1.32; 7.64]
**Total (95% CI)**			**24.00**			**23.00**	**9.1%**	**4.56 [2.00; 7.11]**
Heterogeneity: $Tau^2 = 0$; $Chi^2 = 0.01$, df = 1 (P = 0.94); $I^2 = 0\%$								
Test for overall effect: Z = 3.50 (P < 0.01)								
**Total (95% CI)**			**183.00**			**147.00**	**100.0%**	**2.85 [1.97; 3.74]**
Heterogeneity: $Tau^2 = 1.02$; $Chi^2 = 20.42$, df = 12 (P = 0.06); $I^2 = 41\%$								
Test for overall effect: Z = 6.31 (P < 0.01)								

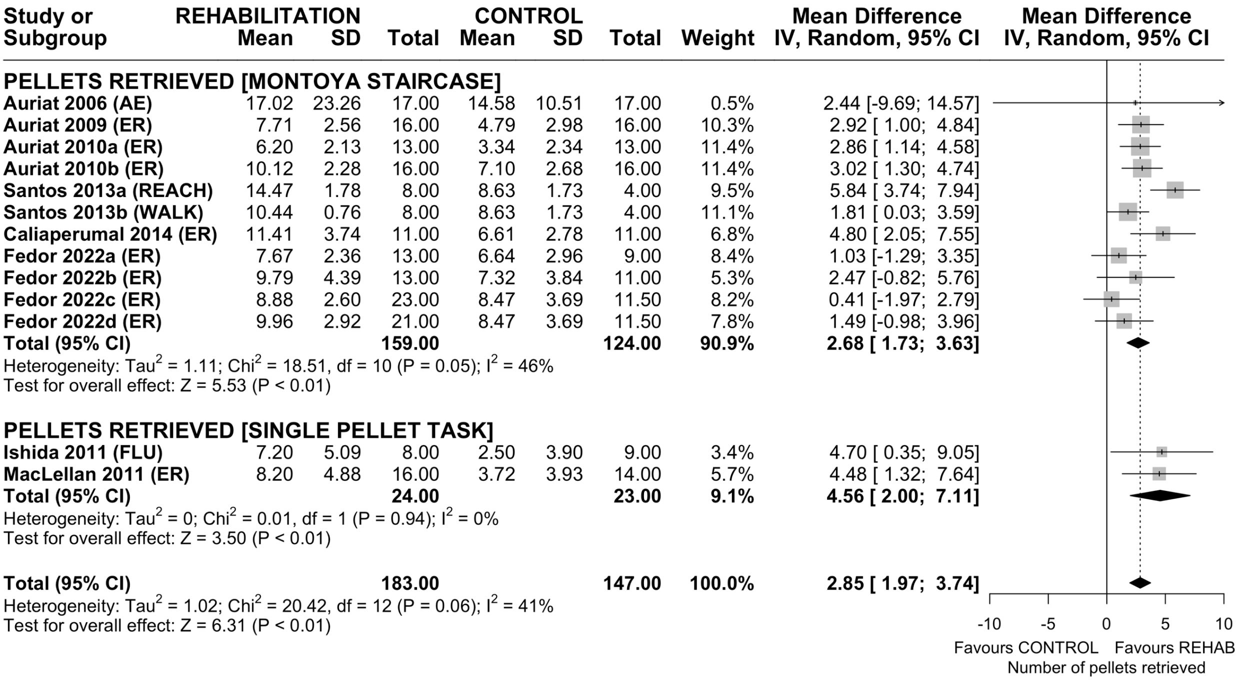

Fig. S5 Forest plot of the post-hoc random-effects meta-analysis of interventions that reported the number of pellets retrieved in their respective skilled reaching endpoints (n=13). Rehabilitation improved skilled reaching success [MD 2.85 pellets retrieved (95% CI 1.97-3.74), p<0.01; SMD 0.82 (95% CI 0.51-1.13), p<0.01] to a similar extent as observed in our full analysis [SMD 0.75 (95% CI 0.50-1.01), p<0.01]. While an overall treatment effect is evident, the mean difference in pellets retrieved between treated and untreated animals fails to reach or exceed the 3-pellet threshold we have argued to be of functional importance (i.e., one level of Montoya staircase). Effect sizes presented as mean difference (MD), number of pellets retrieved, with 95% CI

Study or Subgroup	REHABILITATION Mean	SD	Total	CONTROL Mean	SD	Total	Weight	Mean Difference IV, Random, 95% CI
**CIMT+FLU**								
**DeBow 2003a (FLU)**	17.59	19.06	9.00	12.57	15.08	3.66	4.1%	5.02 [-14.82; 24.86]
**DeBow 2003b (CIMT)**	30.37	22.20	11.00	12.57	15.08	3.67	4.0%	17.80 [-2.45; 38.05]
**MacLellan 2005 (CIMT)**	24.64	16.89	15.00	13.73	16.54	15.00	7.4%	10.91 [-1.05; 22.87]
**Ishida 2011 (FLU)**	30.60	10.18	8.00	25.30	3.00	9.00	10.4%	5.30 [-2.02; 12.62]
**Total (95% CI)**			**43.00**			**31.33**	**26.0%**	**7.55 [1.84; 13.27]**
Heterogeneity: $Tau^2 = 0$; $Chi^2 = 1.71$, df = 3 (P = 0.63); $I^2 = 0\%$								
Test for overall effect: Z = 2.59 (P < 0.01)								
**ENRICHED REHABILITATION**								
**Auriat 2009 (ER)**	23.67	14.68	16.00	34.42	13.08	16.00	8.9%	-10.75 [-20.38; -1.12]
**Auriat 2010a (ER)**	35.44	15.90	13.00	34.85	15.90	13.00	7.3%	0.59 [-11.63; 12.81]
**Auriat 2010b (ER)**	42.03	30.28	16.00	38.92	21.88	16.00	4.6%	3.11 [-15.20; 21.42]
**Total (95% CI)**			**45.00**			**45.00**	**20.8%**	**-4.18 [-13.03; 4.67]**
Heterogeneity: $Tau^2 = 19.84$; $Chi^2 = 2.93$, df = 2 (P = 0.23); $I^2 = 32\%$								
Test for overall effect: Z = -0.93 (P = 0.35)								
**SKILLED REACHING**								
**Mestriner 2011a (REACH)**	37.93	5.47	12.00	25.43	5.47	6.00	11.7%	12.50 [7.14; 17.86]
**Kim 2012a (REACH)**	45.13	10.53	15.00	25.08	6.70	7.50	10.5%	20.05 [12.88; 27.22]
**Kim 2012b (REACH-Ipsi)**	36.14	10.22	15.00	25.08	6.70	7.50	10.6%	11.06 [4.01; 18.11]
**Total (95% CI)**			**42.00**			**21.00**	**32.9%**	**14.30 [9.22; 19.39]**
Heterogeneity: $Tau^2 = 9.32$; $Chi^2 = 3.7$, df = 2 (P = 0.16); $I^2 = 46\%$								
Test for overall effect: Z = 5.51 (P < 0.01)								
**OTHER**								
**DeBow 2003c (EX)**	35.39	39.37	9.00	12.57	15.08	3.67	2.2%	22.82 [-7.17; 52.81]
**Nguyen 2008 (EE)**	28.31	16.46	14.00	23.08	21.54	16.00	6.5%	5.23 [-8.40; 18.86]
**Mestriner 2011b (WALK)**	28.69	5.82	12.00	25.43	5.47	6.00	11.7%	3.26 [-2.22; 8.74]
**Total (95% CI)**			**35.00**			**25.67**	**20.4%**	**4.07 [-0.94; 9.08]**
Heterogeneity: $Tau^2 = 0$; $Chi^2 = 1.61$, df = 2 (P = 0.45); $I^2 = 0\%$								
Test for overall effect: Z = 1.59 (P = 0.11)								
**Total (95% CI)**			**165.00**			**123.00**	**100.0%**	**7.49 [2.66; 12.31]**
Heterogeneity: $Tau^2 = 44.16$; $Chi^2 = 36.22$, df = 12 (P < 0.01); $I^2 = 67\%$								
Test for overall effect: Z = 3.04 (P < 0.01)								

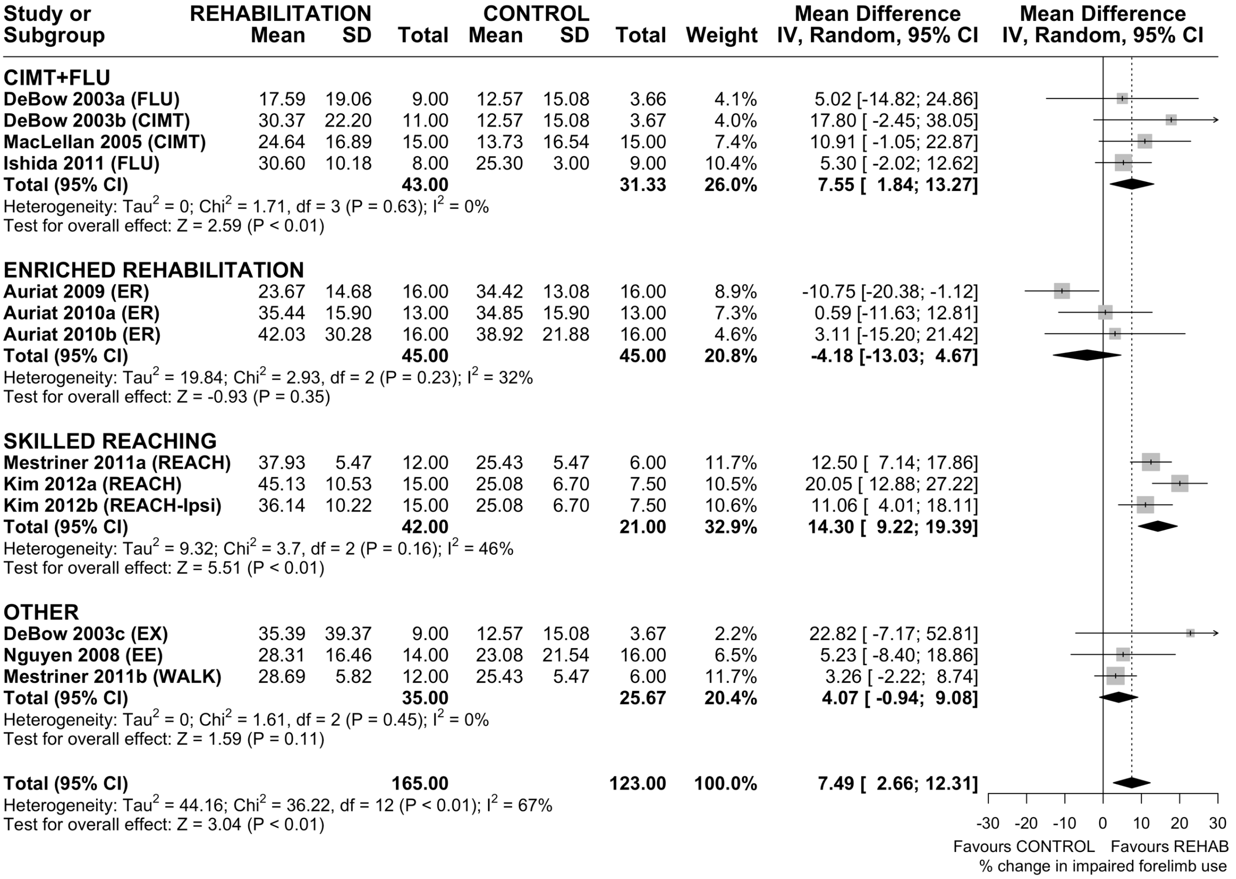

Fig. S6 Forest plot of the sensitivity analysis conducted to evaluate the impact of research quality on recovery of spontaneous impaired forelimb use. Interventions from articles with a CAMARADES score of 0-3 were removed (n=2) and random-effects meta-analysis was conducted (n=13). We observed a similar overall treatment effect [MD 7.49% increase in impaired forelimb use (95% CI 2.66-12.31), p<0.01] to our original model; removing low quality studies did not improve heterogeneity (I^2=67%, p<0.01). Effect sizes presented as mean difference (MD), percent change in impaired forelimb use, with 95% CI

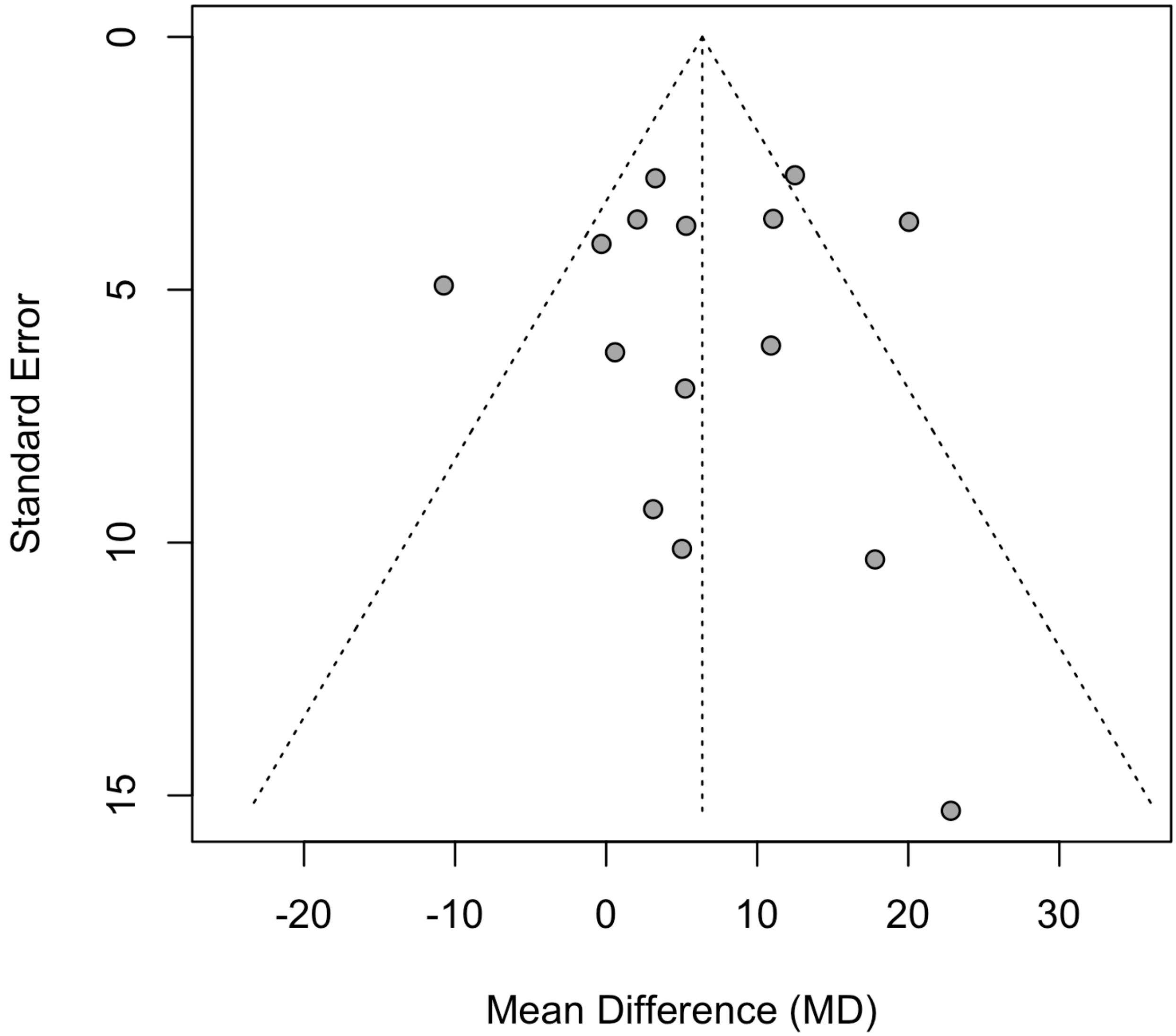

Fig. S7 Funnel plot of spontaneous impaired forelimb use data (Fig. 4). Egger regression did not indicate the presence of asymmetry in the dataset ($p>0.05$), therefore trim-and-fill analysis was not completed. Effect sizes presented as mean difference (MD), percent change in impaired forelimb use

Study or Subgroup	REHABILITATION Mean	SD	Total	CONTROL Mean	SD	Total	Weight	Std. Mean Difference IV, Random, 95% CI
**CIMT+FLU**								
**DeBow 2003a (FLU)**	-21.79	7.56	9.00	-24.28	9.25	3.66	4.5%	0.29 [-0.93; 1.51]
**DeBow 2003b (CIMT)**	-14.53	9.05	11.00	-24.28	9.25	3.67	4.4%	1.01 [-0.25; 2.26]
**MacLellan 2005 (CIMT)**	-12.30	9.22	15.00	-12.04	8.95	15.00	8.2%	-0.03 [-0.74; 0.69]
**Ishida 2011 (FLU)**	66.20	16.97	8.00	42.20	24.00	9.00	5.6%	1.08 [0.05; 2.12]
**Total (95% CI)**			**43.00**			**31.33**	**22.7%**	**0.48 [-0.10; 1.06]**
Heterogeneity: $Tau^2 = 0.09$; $Chi^2 = 3.96$, df = 3 (P = 0.27); $I^2 = 24\%$								
Test for overall effect: Z = 1.62 (P = 0.11)								
**ENRICHED REHABILITATION**								
**Auriat 2008 (ER)**	24.02	9.89	12.00	15.87	14.89	10.00	6.9%	0.63 [-0.23; 1.50]
**Auriat 2009 (ER)**	85.41	11.04	16.00	80.20	14.68	16.00	8.4%	0.39 [-0.31; 1.09]
**Auriat 2010a (ER)**	-17.45	17.02	13.00	-34.38	17.52	13.00	7.3%	0.95 [0.13; 1.77]
**Auriat 2010b (ER)**	-10.65	12.04	16.00	-32.71	11.04	16.00	7.0%	1.86 [1.02; 2.71]
**Caliaperumal 2014 (ER)**	96.54	4.40	11.00	69.74	17.17	11.00	5.4%	2.06 [0.99; 3.13]
**Fedor 2022a (ER)**	93.46	6.93	13.00	91.84	5.50	9.00	7.0%	0.24 [-0.61; 1.10]
**Fedor 2022b (ER)**	95.34	8.78	10.00	100.00	0.00	11.00	0.0%	
**Total (95% CI)**			**91.00**			**86.00**	**41.9%**	**0.98 [0.40; 1.56]**
Heterogeneity: $Tau^2 = 0.34$; $Chi^2 = 14.13$, df = 5 (P = 0.01); $I^2 = 65\%$								
Test for overall effect: Z = 3.30 (P < 0.01)								
**SKILLED REACHING**								
**Mestriner 2011a (REACH)**	-15.62	4.16	12.00	-32.45	13.61	6.00	4.6%	1.92 [0.71; 3.13]
**Kim 2012a (REACH)**	-31.00	12.47	15.00	-49.10	10.91	7.50	5.9%	1.45 [0.46; 2.44]
**Kim 2012b (REACH-Ipsi)**	-39.98	10.38	15.00	-49.10	10.91	7.50	6.5%	0.83 [-0.08; 1.75]
**Total (95% CI)**			**42.00**			**21.00**	**17.0%**	**1.31 [0.71; 1.92]**
Heterogeneity: $Tau^2 = 0.01$; $Chi^2 = 2.1$, df = 2 (P = 0.35); $I^2 = 5\%$								
Test for overall effect: Z = 4.26 (P < 0.01)								
**OTHER**								
**DeBow 2003c (EX)**	-20.50	13.73	9.00	-24.28	9.25	3.67	4.5%	0.27 [-0.95; 1.50]
**Nguyen 2008 (EE)**	82.59	9.95	14.00	75.82	12.47	16.00	8.0%	0.58 [-0.15; 1.31]
**Mestriner 2011b (WALK)**	-24.76	16.94	12.00	-32.45	13.61	6.00	5.9%	0.46 [-0.54; 1.45]
**Total (95% CI)**			**35.00**			**25.67**	**18.5%**	**0.49 [-0.04; 1.02]**
Heterogeneity: $Tau^2 = 0$; $Chi^2 = 0.18$, df = 2 (P = 0.91); $I^2 = 0\%$								
Test for overall effect: Z = 1.80 (P = 0.07)								
**Total (95% CI)**			**211.00**			**164.00**	**100.0%**	**0.83 [0.52; 1.15]**
Heterogeneity: $Tau^2 = 0.18$; $Chi^2 = 27.25$, df = 15 (P = 0.03); $I^2 = 45\%$								
Test for overall effect: Z = 5.20 (P < 0.01)								

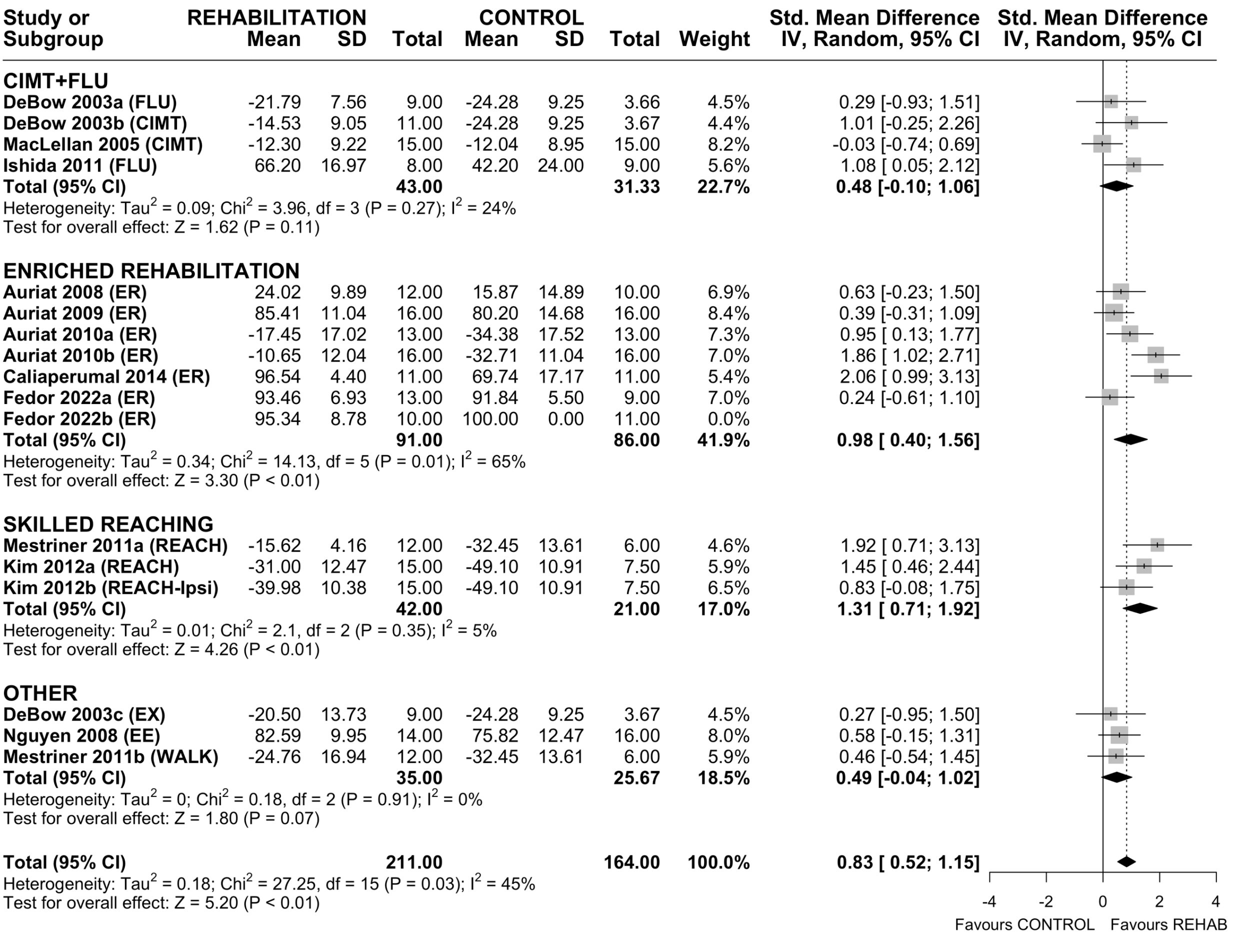

Fig. S8 Forest plot of the sensitivity analysis conducted to evaluate the impact of research quality on recovery of locomotor function. Interventions from articles with a CAMARADES score of 0-3 were removed (n=10) and random-effects meta-analysis was conducted (n=16). We observed a similar overall treatment effect [SMD 0.83 (95% CI 0.52-1.15), p<0.01] to our original model and found removing low quality studies somewhat reduced heterogeneity (I^2=45%, p=0.03). Effect sizes presented as Hedge's *G* standardized mean difference (SMD) with 95% CI

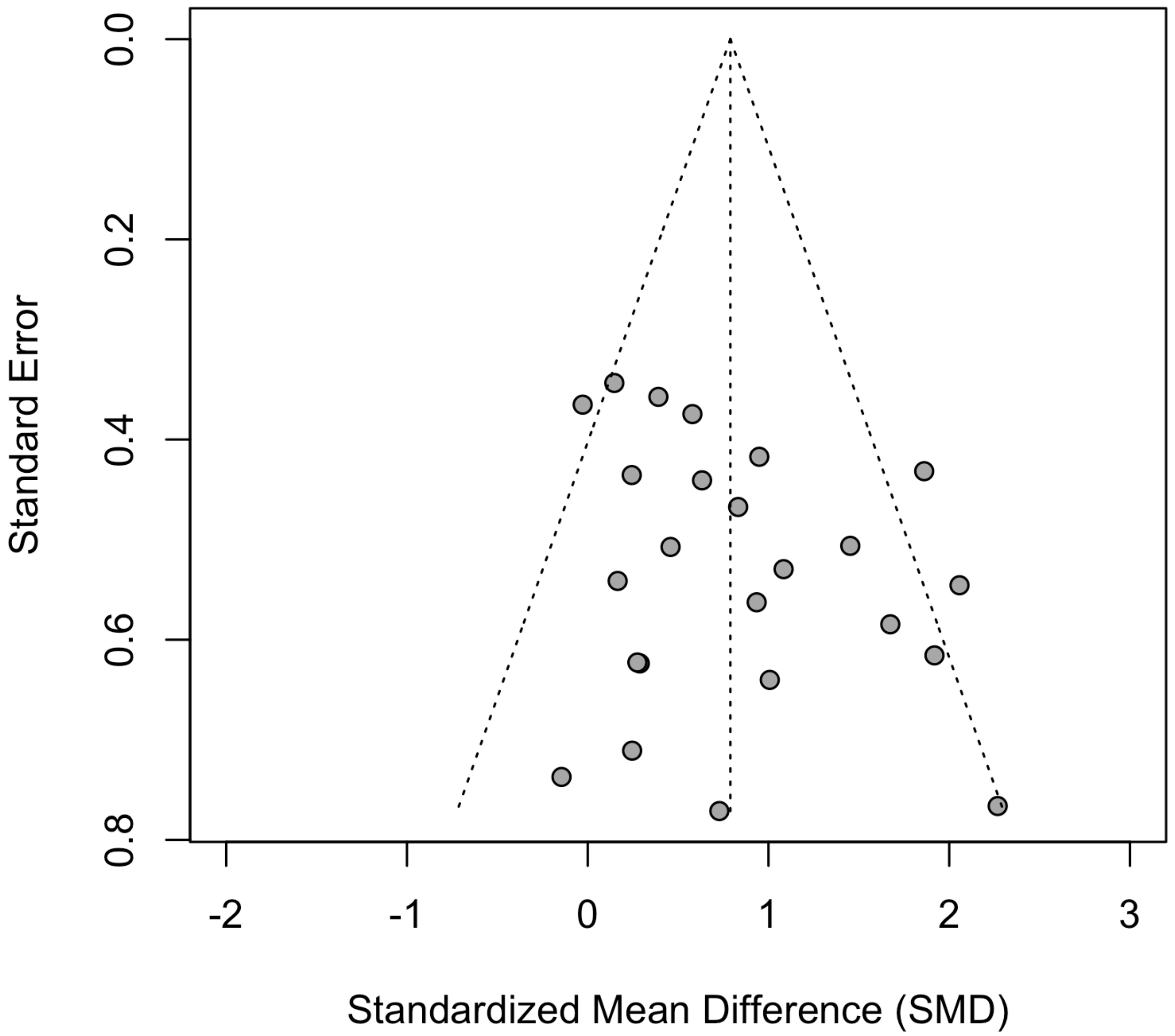

Fig. S9 Funnel plot of locomotor function data (Fig.5). Egger regression did not indicate the presence of asymmetry in the dataset ($p>0.05$), therefore trim-and-fill analysis was not completed. Effect sizes presented as Hedge's *G* standardized mean difference (SMD)

Study or Subgroup	REHABILITATION Mean	SD	Total	CONTROL Mean	SD	Total	Weight	Std. Mean Difference IV, Random, 95% CI
**MILD**								
**Auriat 2010b [ER]**	10.12	2.28	16.00	7.10	2.68	16.00	5.3%	1.18 [0.42; 1.94]
**Ishida 2011 [FLU]**	7.20	5.09	8.00	2.50	3.90	9.00	3.8%	0.99 [-0.03; 2.02]
**Ishida 2015a [FLU]**	28.30	21.78	8.00	7.10	13.80	9.00	3.7%	1.12 [0.08; 2.16]
**Ishida 2015b [FLU]**	21.30	7.84	6.00	12.30	18.00	9.00	3.7%	0.57 [-0.49; 1.63]
**Total (95% CI)**			**38.00**			**43.00**	**16.5%**	**1.01 [0.54; 1.48]**
Heterogeneity: $Tau^2 = 0$; $Chi^2 = 0.92$, df = 3 (P = 0.82); $I^2 = 0\%$								
Test for overall effect: Z = 4.19 (P < 0.01)								
**MODERATE**								
**DeBow 2003a [FLU]**	47.65	36.35	9.00	34.60	28.67	3.66	3.0%	0.35 [-0.88; 1.58]
**DeBow 2003b [CIMT]**	77.24	26.46	11.00	34.60	28.67	3.67	2.7%	1.49 [0.15; 2.82]
**DeBow 2003c [EX]**	42.83	21.19	9.00	34.60	28.67	3.67	3.0%	0.33 [-0.90; 1.55]
**Auriat 2008 [ER]**	37.98	26.55	12.00	49.21	30.58	10.00	4.7%	-0.38 [-1.23; 0.47]
**Auriat 2009 [ER]**	7.71	2.56	16.00	4.79	2.98	16.00	5.4%	1.02 [0.28; 1.77]
**Mestriner 2011a [REACH]**	65.37	9.91	12.00	47.43	12.09	6.00	3.3%	1.61 [0.46; 2.75]
**Mestriner 2011b [WALK]**	53.14	10.98	12.00	47.43	12.09	6.00	3.9%	0.48 [-0.52; 1.48]
**Fedor 2022c [ER]**	8.88	2.60	23.00	8.47	3.69	11.50	5.6%	0.13 [-0.57; 0.84]
**Fedor 2022d [ER]**	9.96	2.92	21.00	8.47	3.69	11.50	5.5%	0.45 [-0.28; 1.18]
**Total (95% CI)**			**125.00**			**72.00**	**37.2%**	**0.54 [0.14; 0.94]**
Heterogeneity: $Tau^2 = 0.14$; $Chi^2 = 12.92$, df = 8 (P = 0.11); $I^2 = 38\%$								
Test for overall effect: Z = 2.64 (P < 0.01)								
**SEVERE**								
**MacLellan 2005 [CIMT]**	13.44	12.33	15.00	12.82	12.76	15.00	5.6%	0.05 [-0.67; 0.76]
**Auriat 2006 [AE]**	17.02	23.26	17.00	14.58	10.51	17.00	5.9%	0.13 [-0.54; 0.81]
**Nguyen 2008 [EE]**	36.20	18.99	14.00	25.82	18.96	16.00	5.5%	0.53 [-0.20; 1.26]
**Total (95% CI)**			**46.00**			**48.00**	**16.9%**	**0.23 [-0.18; 0.64]**
Heterogeneity: $Tau^2 = 0$; $Chi^2 = 0.99$, df = 2 (P = 0.61); $I^2 = 0\%$								
Test for overall effect: Z = 1.10 (P = 0.27)								
**UNCLEAR**								
**Auriat 2010a [ER]**	6.20	2.13	13.00	3.34	2.34	13.00	4.7%	1.24 [0.39; 2.09]
**MacLellan 2011 [ER]**	8.20	4.88	16.00	3.72	3.93	14.00	5.2%	0.98 [0.21; 1.74]
**Santos 2013a [REACH]**	14.47	1.78	8.00	8.63	1.73	4.00	1.5%	3.05 [1.15; 4.95]
**Santos 2013b [WALK]**	10.44	0.76	8.00	8.63	1.73	4.00	2.5%	1.46 [0.07; 2.86]
**Caliaperumal 2014 [ER]**	11.41	3.74	11.00	6.61	2.78	11.00	4.2%	1.40 [0.45; 2.35]
**Ishida 2016 [FLU]**	42.20	10.05	7.00	10.00	10.06	6.00	1.8%	2.98 [1.24; 4.72]
**Fedor 2022a [ER]**	7.67	2.36	13.00	6.64	2.96	9.00	4.7%	0.38 [-0.48; 1.24]
**Fedor 2022b [ER]**	9.79	4.39	13.00	7.32	3.84	11.00	4.9%	0.57 [-0.25; 1.40]
**Total (95% CI)**			**89.00**			**72.00**	**29.5%**	**1.22 [0.70; 1.73]**
Heterogeneity: $Tau^2 = 0.25$; $Chi^2 = 13.66$, df = 7 (P = 0.06); $I^2 = 49\%$								
Test for overall effect: Z = 4.63 (P < 0.01)								
**Total (95% CI)**			**298.00**			**235.00**	**100.0%**	**0.75 [0.50; 1.01]**
Heterogeneity: $Tau^2 = 0.17$; $Chi^2 = 41.51$, df = 23 (P = 0.01); $I^2 = 45\%$								
Test for overall effect: Z = 5.78 (P < 0.01)								

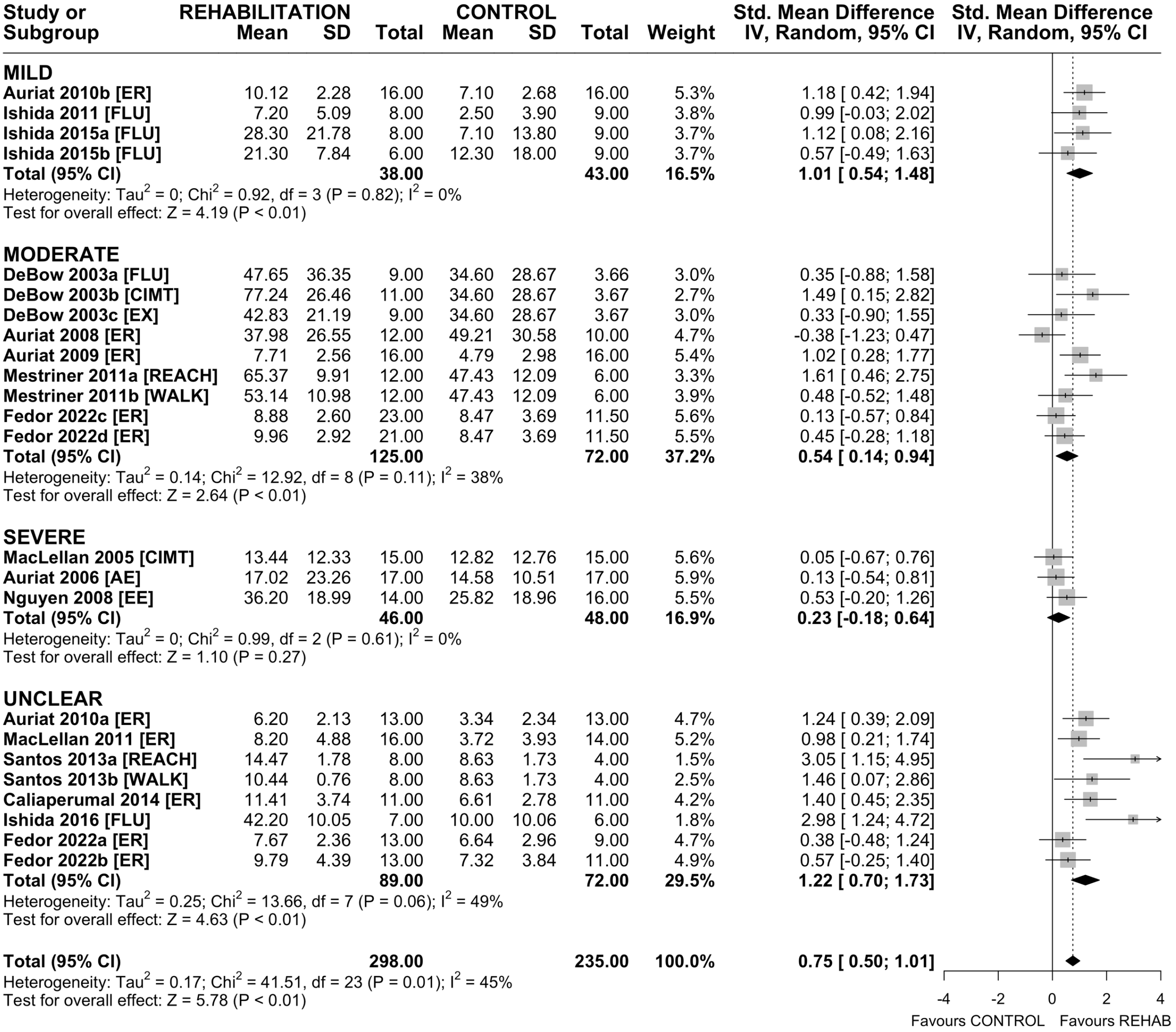

Fig. S10 Forest plot of random-effects meta-analysis of skilled reaching recovery grouped by lesion size reported in the study's untreated control group. Severity was grouped into mild [≤30 mm^3], moderate [31-60 mm^3], severe [≥61 mm^3], and UNCLEAR. Rehabilitation improved skilled reaching recovery in animals with mild and moderate, but not severe ICH. Effect sizes presented as Hedge's *G* standardized mean difference (SMD) with 95% CI

Study or Subgroup	REHABILITATION Mean	SD	Total	CONTROL Mean	SD	Total	Weight	Mean Difference IV, Random, 95% CI
**MILD**								
**Auriat 2010b (ER)**	42.03	30.28	16.00	38.92	21.88	16.00	3.7%	3.11 [-15.20; 21.42]
**Ishida 2011 (FLU)**	30.60	10.18	8.00	25.30	3.00	9.00	8.7%	5.30 [-2.02; 12.62]
**Total (95% CI)**			**24.00**			**25.00**	**12.4%**	**5.00 [-1.80; 11.80]**
Heterogeneity: $Tau^2 = 0$; $Chi^2 = 0.05$, df = 1 (P = 0.83); $I^2 = 0\%$								
Test for overall effect: Z = 1.44 (P = 0.15)								
**MODERATE**								
**DeBow 2003a (FLU)**	17.59	19.06	9.00	12.57	15.08	3.66	3.3%	5.02 [-14.82; 24.86]
**DeBow 2003b (CIMT)**	30.37	22.20	11.00	12.57	15.08	3.67	3.2%	17.80 [-2.45; 38.05]
**DeBow 2003c (EX)**	35.39	39.37	9.00	12.57	15.08	3.67	1.7%	22.82 [-7.17; 52.81]
**Auriat 2009 (ER)**	23.67	14.68	16.00	34.42	13.08	16.00	7.3%	-10.75 [-20.38; -1.12]
**Mestriner 2011a (REACH)**	37.93	5.47	12.00	25.43	5.47	6.00	9.9%	12.50 [7.14; 17.86]
**Mestriner 2011b (WALK)**	28.69	5.82	12.00	25.43	5.47	6.00	9.8%	3.26 [-2.22; 8.74]
**Total (95% CI)**			**69.00**			**39.00**	**35.3%**	**5.78 [-3.02; 14.58]**
Heterogeneity: $Tau^2 = 72.99$; $Chi^2 = 20.74$, df = 5 (P < 0.01); $I^2 = 76\%$								
Test for overall effect: Z = 1.29 (P = 0.20)								
**SEVERE**								
**MacLellan 2005 (CIMT)**	24.64	16.89	15.00	13.73	16.54	15.00	6.1%	10.91 [-1.05; 22.87]
**Auriat 2006 (AE)**	9.67	8.95	17.00	9.98	14.31	17.00	8.3%	-0.31 [-8.33; 7.71]
**Nguyen 2008 (EE)**	28.31	16.46	14.00	23.08	21.54	16.00	5.3%	5.23 [-8.40; 18.86]
**Total (95% CI)**			**46.00**			**48.00**	**19.7%**	**3.93 [-2.81; 10.68]**
Heterogeneity: $Tau^2 = 6.35$; $Chi^2 = 2.4$, df = 2 (P = 0.30); $I^2 = 17\%$								
Test for overall effect: Z = 1.14 (P = 0.25)								
**UNCLEAR**								
**Auriat 2010a (ER)**	35.44	15.90	13.00	34.85	15.90	13.00	6.0%	0.59 [-11.63; 12.81]
**Kim 2012a (REACH)**	45.13	10.53	15.00	25.08	6.70	7.50	8.8%	20.05 [12.88; 27.22]
**Kim 2012b (REACH-Ipsi)**	36.14	10.22	15.00	25.08	6.70	7.50	8.9%	11.06 [4.01; 18.11]
**Takamatsu 2016 (AE)**	38.95	11.37	14.00	36.89	7.30	14.00	8.9%	2.06 [-5.02; 9.14]
**Total (95% CI)**			**57.00**			**42.00**	**32.5%**	**8.95 [0.06; 17.85]**
Heterogeneity: $Tau^2 = 64.10$; $Chi^2 = 14.75$, df = 3 (P < 0.01); $I^2 = 80\%$								
Test for overall effect: Z = 1.97 (P = 0.05)								
**Total (95% CI)**			**196.00**			**154.00**	**100.0%**	**6.36 [2.09; 10.64]**
Heterogeneity: $Tau^2 = 40.75$; $Chi^2 = 42.03$, df = 14 (P < 0.01); $I^2 = 67\%$								
Test for overall effect: Z = 2.92 (P < 0.01)								

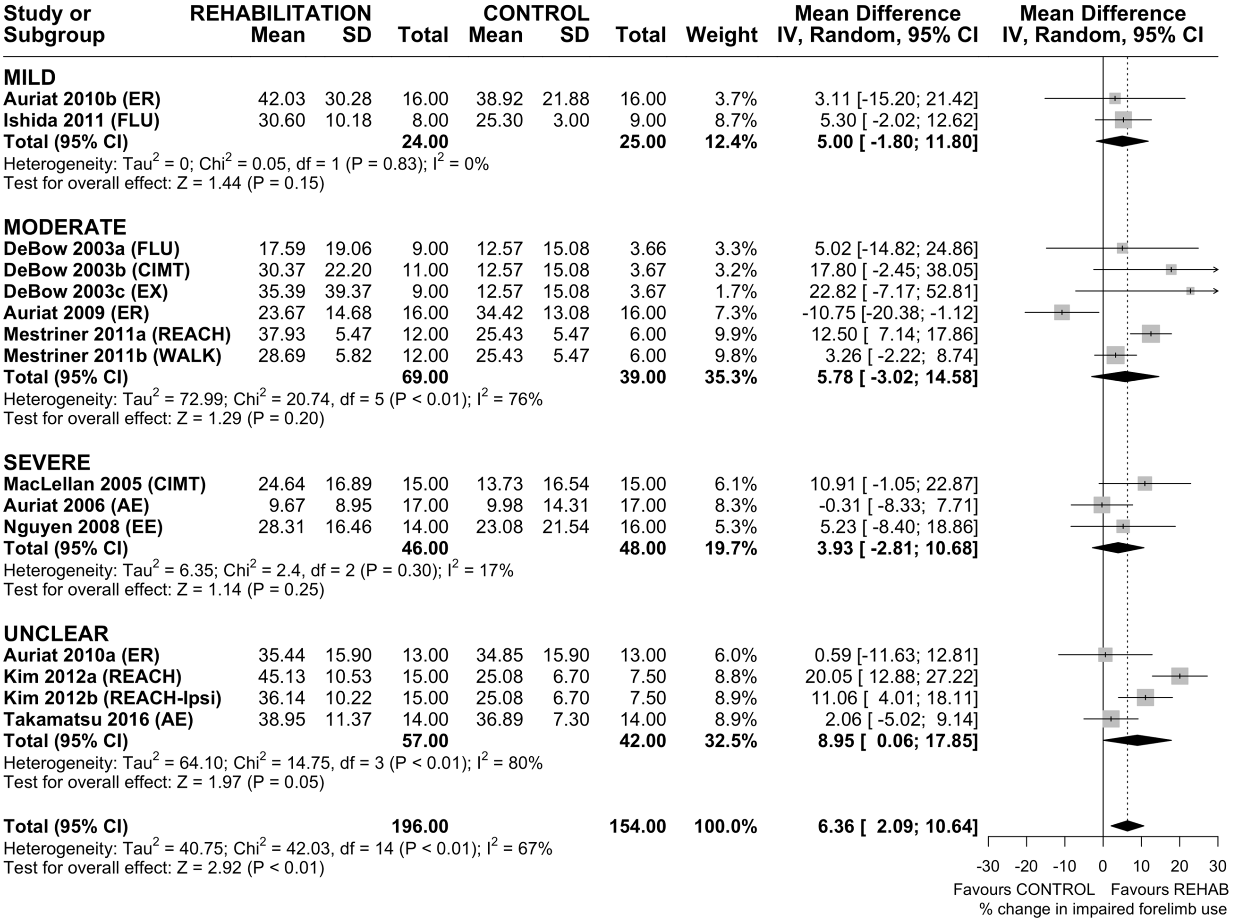

Fig. S11 Forest plot of random effects meta-analysis of recovery of spontaneous impaired forelimb use grouped by lesion size reported in the study's untreated control group. Severity was grouped into mild [≤30 mm^3], moderate [31-60 mm^3], severe [≥61 mm^3], and UNCLEAR. While rehabilitation increased spontaneous impaired forelimb use, there was no discernable effect of rehabilitation in groups with known severity. Effect sizes presented as mean difference (MD), percent change in impaired forelimb use, with 95% CI

Study or Subgroup	REHABILITATION Mean	SD	Total	CONTROL Mean	SD	Total	Weight	Std. Mean Difference IV, Random, 95% CI
**MILD**								
**Auriat 2010b (ER)**	-10.65	12.04	16.00	-32.71	11.04	16.00	5.1%	1.86 [1.02; 2.71]
**Ishida 2011 (FLU)**	66.20	16.97	8.00	42.20	24.00	9.00	4.0%	1.08 [0.05; 2.12]
**Ishida 2015a (FLU)**	-9.20	5.09	8.00	-20.00	6.90	9.00	3.6%	1.67 [0.53; 2.82]
**Ishida 2015b (FLU)**	-12.50	2.20	6.00	-18.50	7.50	9.00	3.8%	0.93 [-0.17; 2.04]
**Total (95% CI)**			**38.00**			**43.00**	**16.5%**	**1.45 [0.94; 1.95]**
Heterogeneity: Tau^2 = 0; Chi^2 = 2.37, df = 3 (P = 0.50); I^2 = 0%								
Test for overall effect: Z = 5.60 (P < 0.01)								
**MODERATE**								
**DeBow 2003a (FLU)**	-21.79	7.56	9.00	-24.28	9.25	3.66	3.3%	0.29 [-0.93; 1.51]
**DeBow 2003b (CIMT)**	-14.53	9.05	11.00	-24.28	9.25	3.67	3.2%	1.01 [-0.25; 2.26]
**DeBow 2003c (EX)**	-20.50	13.73	9.00	-24.28	9.25	3.67	3.3%	0.27 [-0.95; 1.50]
**Auriat 2008 (ER)**	24.02	9.89	12.00	15.87	14.89	10.00	5.0%	0.63 [-0.23; 1.50]
**Auriat 2009 (ER)**	85.41	11.04	16.00	80.20	14.68	16.00	6.0%	0.39 [-0.31; 1.09]
**Mestriner 2011a (REACH)**	-15.62	4.16	12.00	-32.45	13.61	6.00	3.3%	1.92 [0.71; 3.13]
**Mestriner 2011b (WALK)**	-24.76	16.94	12.00	-32.45	13.61	6.00	4.3%	0.46 [-0.54; 1.45]
**Total (95% CI)**			**81.00**			**49.00**	**28.3%**	**0.63 [0.25; 1.01]**
Heterogeneity: Tau^2 = 0; Chi^2 = 5.91, df = 6 (P = 0.43); I^2 = 0%								
Test for overall effect: Z = 3.28 (P < 0.01)								
**SEVERE**								
**MacLellan 2005 (CIMT)**	-12.30	9.22	15.00	-12.04	8.95	15.00	5.9%	-0.03 [-0.74; 0.69]
**Auriat 2006 (AE)**	-11.04	10.93	17.00	-12.74	11.59	17.00	6.2%	0.15 [-0.53; 0.82]
**Nguyen 2008 (EE)**	82.59	9.95	14.00	75.82	12.47	16.00	5.8%	0.58 [-0.15; 1.31]
**Total (95% CI)**			**46.00**			**48.00**	**18.0%**	**0.22 [-0.18; 0.63]**
Heterogeneity: Tau^2 = 0; Chi^2 = 1.43, df = 2 (P = 0.49); I^2 = 0%								
Test for overall effect: Z = 1.08 (P = 0.28)								
**UNCLEAR**								
**Auriat 2010a (ER)**	-17.45	17.02	13.00	-34.38	17.52	13.00	5.3%	0.95 [0.13; 1.77]
**Kim 2012a (REACH)**	-31.00	12.47	15.00	-49.10	10.91	7.50	4.3%	1.45 [0.46; 2.44]
**Kim 2012b (REACH-Ipsi)**	-39.98	10.38	15.00	-49.10	10.91	7.50	4.7%	0.83 [-0.08; 1.75]
**Caliaperumal 2014 (ER)**	96.54	4.40	11.00	69.74	17.17	11.00	3.9%	2.06 [0.99; 3.13]
**Tamakoshi 2014 (AT)**	49.42	17.44	6.00	45.86	21.81	8.00	3.9%	0.17 [-0.90; 1.23]
**Ishida 2016 (FLU)**	-7.60	4.50	7.00	-18.80	4.70	6.00	2.4%	2.27 [0.77; 3.77]
**Tamakoshi 2018a (AE)**	-14.37	7.58	8.00	-16.65	11.40	2.66	2.7%	0.25 [-1.15; 1.64]
**Tamakoshi 2018b (AE)**	-10.48	5.58	6.00	-16.65	11.40	2.67	2.4%	0.73 [-0.78; 2.24]
**Tamakoshi 2018c (AE)**	-18.26	9.21	6.00	-16.65	11.40	2.67	2.6%	-0.14 [-1.59; 1.30]
**Fedor 2022a (ER)**	93.46	6.93	13.00	91.84	5.50	9.00	5.0%	0.24 [-0.61; 1.10]
**Fedor 2022b (ER)**	95.34	8.78	10.00	100.00	0.00	11.00	0.0%	
**Total (95% CI)**			**110.00**			**81.00**	**37.2%**	**0.88 [0.41; 1.34]**
Heterogeneity: Tau^2 = 0.23; Chi^2 = 15.89, df = 9 (P = 0.07); I^2 = 43%								
Test for overall effect: Z = 3.71 (P < 0.01)								
**Total (95% CI)**			**275.00**			**221.00**	**100.0%**	**0.79 [0.52; 1.06]**
Heterogeneity: Tau^2 = 0.18; Chi^2 = 40.10, df = 23 (P = 0.01); I^2 = 43%								
Test for overall effect: Z = 5.76 (P < 0.01)								

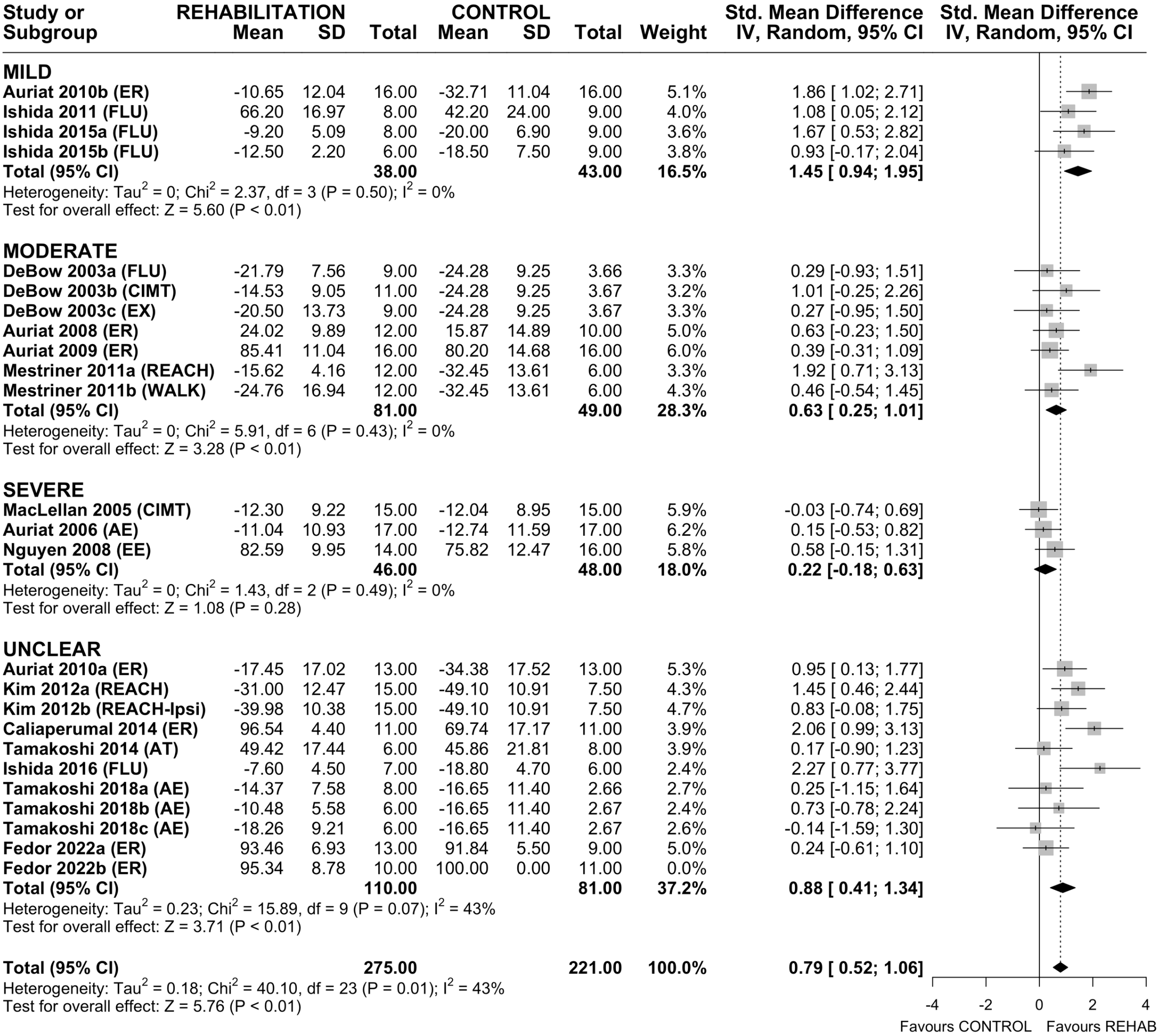

Fig. S12 Forest plot of random-effects meta-analysis of locomotor recovery grouped by lesion size reported in the study's untreated control group. Severity was grouped into mild [≤30 mm^3], moderate [31-60 mm^3], severe [≥61 mm^3], and UNCLEAR. Rehabilitation improved locomotor recovery in animals with mild and moderate, but not severe ICH. Effect sizes presented as Hedge's *G* standardized mean difference (SMD) with 95% CI

a

Study or Subgroup	REHABILITATION Mean	SD	Total	CONTROL Mean	SD	Total	Weight	Std. Mean Difference IV, Random, 95% CI
**FLU [56 HOURS]**								
**DeBow 2003a [FLU]**	47.65	36.35	9.00	34.60	28.67	3.66	13.1%	0.35 [-0.88; 1.58]
**CIMT [FLU 56 HOURS + EX 7 HOURS]**								
**DeBow 2003b [CIMT]**	77.24	26.46	11.00	34.60	28.67	3.67	11.9%	1.49 [0.15; 2.82]
**MacLellan 2005 [CIMT]**	13.44	12.33	15.00	12.82	12.76	15.00	20.4%	0.05 [-0.67; 0.76]
**Total (95% CI)**			**26.00**			**18.67**	**32.3%**	**0.65 [-0.74; 2.04]**
Heterogeneity: $Tau^2 = 0.74$; $Chi^2 = 3.46$, df = 1 (P = 0.06); $I^2 = 71\%$								
Test for overall effect: Z = 0.92 (P = 0.36)								
**FLU [168 HOURS]**								
**Ishida 2011 [FLU]**	7.20	5.09	8.00	2.50	3.90	9.00	15.7%	0.99 [-0.03; 2.02]
**Ishida 2015a [FLU]**	28.30	21.78	8.00	7.10	13.80	9.00	15.4%	1.12 [0.08; 2.16]
**Ishida 2015b [FLU]**	21.30	7.84	6.00	12.30	18.00	9.00	15.2%	0.57 [-0.49; 1.63]
**Ishida 2016 [FLU]**	42.20	10.05	7.00	10.00	10.06	6.00	8.4%	2.98 [1.24; 4.72]
**Total (95% CI)**			**29.00**			**33.00**	**54.6%**	**1.21 [0.42; 2.00]**
Heterogeneity: $Tau^2 = 0.29$; $Chi^2 = 5.49$, df = 3 (P = 0.14); $I^2 = 45\%$								
Test for overall effect: Z = 3.00 (P < 0.01)								
**Total (95% CI)**			**64.00**			**55.33**	**100.0%**	**0.90 [0.30; 1.50]**
Heterogeneity: $Tau^2 = 0.32$; $Chi^2 = 12.38$, df = 6 (P = 0.05); $I^2 = 52\%$								
Test for overall effect: Z = 2.93 (P < 0.01)								

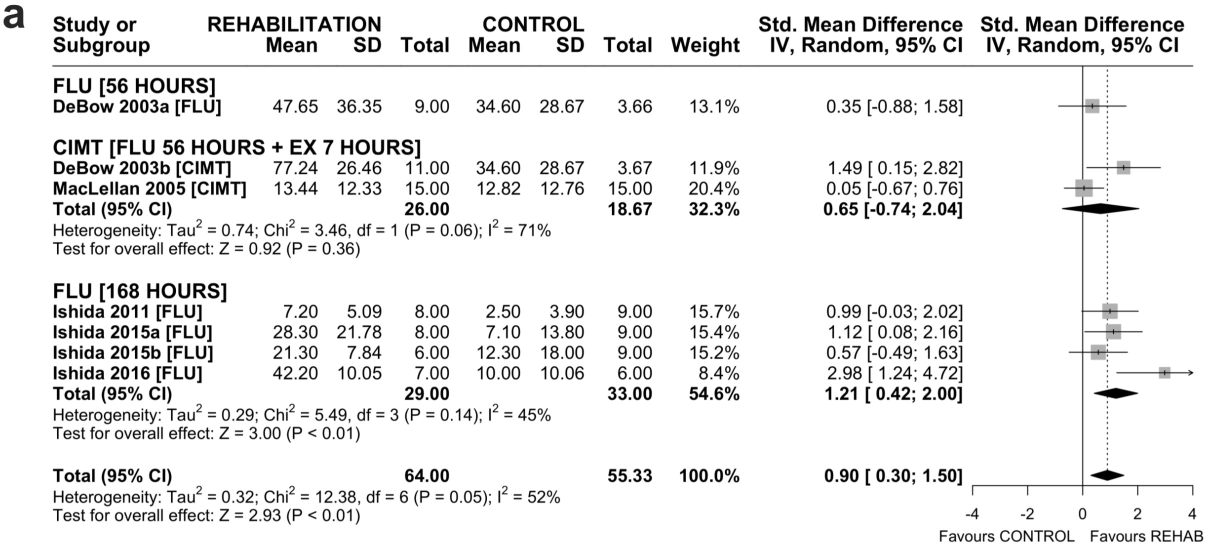

b

Study or Subgroup	REHABILITATION Mean	SD	Total	CONTROL Mean	SD	Total	Weight	Mean Difference IV, Random, 95% CI
**FLU [56 HOURS]**								
**DeBow 2003a (FLU)**	17.59	19.06	9.00	12.57	15.08	3.66	8.3%	5.02 [-14.82; 24.86]
**CIMT [FLU 56 HOURS + EX 7 HOURS]**								
**DeBow 2003b (CIMT)**	30.37	22.20	11.00	12.57	15.08	3.67	8.0%	17.80 [-2.45; 38.05]
**MacLellan 2005 (CIMT)**	24.64	16.89	15.00	13.73	16.54	15.00	22.8%	10.91 [-1.05; 22.87]
**Total (95% CI)**			**26.00**			**18.67**	**30.8%**	**12.69 [2.39; 22.99]**
Heterogeneity: $Tau^2 = 0$; $Chi^2 = 0.33$, df = 1 (P = 0.57); $I^2 = 0\%$								
Test for overall effect: Z = 2.42 (P = 0.02)								
**FLU [168 HOURS]**								
**Ishida 2011 (FLU)**	30.60	10.18	8.00	25.30	3.00	9.00	60.9%	5.30 [-2.02; 12.62]
**Total (95% CI)**			**43.00**			**31.33**	**100.0%**	**7.55 [1.84; 13.27]**
Heterogeneity: $Tau^2 = 0$; $Chi^2 = 1.71$, df = 3 (P = 0.63); $I^2 = 0\%$								
Test for overall effect: Z = 2.59 (P < 0.01)								

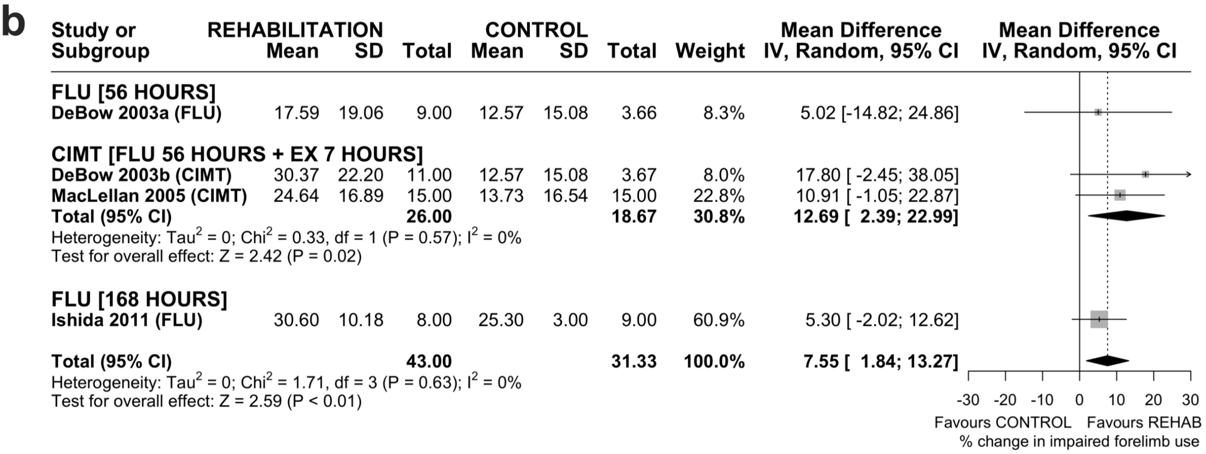

c

Study or Subgroup	REHABILITATION Mean	SD	Total	CONTROL Mean	SD	Total	Weight	Std. Mean Difference IV, Random, 95% CI
**FLU [56 HOURS]**								
**DeBow 2003a (FLU)**	-21.79	7.56	9.00	-24.28	9.25	3.66	13.0%	0.29 [-0.93; 1.51]
**CIMT [FLU 56 HOURS + EX 7 HOURS]**								
**DeBow 2003b (CIMT)**	-14.53	9.05	11.00	-24.28	9.25	3.67	12.6%	1.01 [-0.25; 2.26]
**MacLellan 2005 (CIMT)**	-12.30	9.22	15.00	-12.04	8.95	15.00	20.4%	-0.03 [-0.74; 0.69]
**Total (95% CI)**			**26.00**			**18.67**	**33.0%**	**0.36 [-0.62; 1.34]**
Heterogeneity: $Tau^2 = 0.26$; $Chi^2 = 1.97$, df = 1 (P = 0.16); $I^2 = 49\%$								
Test for overall effect: Z = 0.71 (P = 0.48)								
**FLU [168 HOURS]**								
**Ishida 2011 (FLU)**	66.20	16.97	8.00	42.20	24.00	9.00	15.4%	1.08 [0.05; 2.12]
**Ishida 2015a (FLU)**	-9.20	5.09	8.00	-20.00	6.90	9.00	13.9%	1.67 [0.53; 2.82]
**Ishida 2015b (FLU)**	-12.50	2.20	6.00	-18.50	7.50	9.00	14.5%	0.93 [-0.17; 2.04]
**Ishida 2016 (FLU)**	-7.60	4.50	7.00	-18.80	4.70	6.00	10.1%	2.27 [0.77; 3.77]
**Total (95% CI)**			**29.00**			**33.00**	**54.0%**	**1.37 [0.79; 1.95]**
Heterogeneity: $Tau^2 = 0$; $Chi^2 = 2.54$, df = 3 (P = 0.47); $I^2 = 0\%$								
Test for overall effect: Z = 4.62 (P < 0.01)								
**Total (95% CI)**			**64.00**			**55.33**	**100.0%**	**0.92 [0.33; 1.52]**
Heterogeneity: $Tau^2 = 0.32$; $Chi^2 = 12.16$, df = 6 (P = 0.06); $I^2 = 51\%$								
Test for overall effect: Z = 3.06 (P < 0.01)								

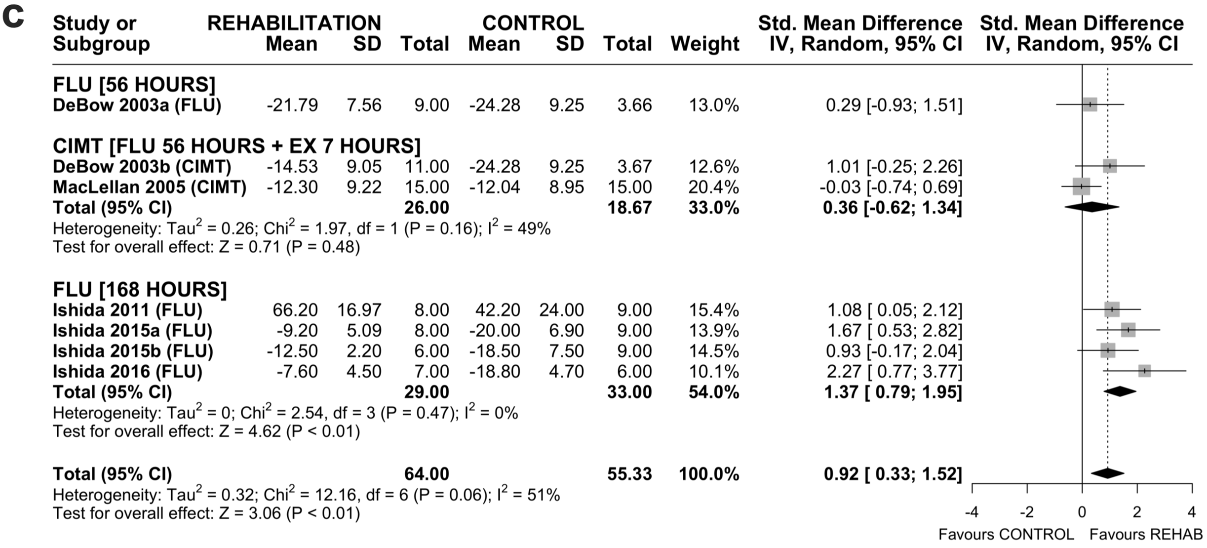

Fig. S13 Impact of treatment dose on recovery in CIMT+FLU interventions. CIMT+FLU interventions were divided into three treatment doses: FLU (56 hours), CIMT (FLU 56 hours + EX 7 hours), and FLU (168 hours). **a** Forest plot of random-effects meta-analysis of skilled reaching recovery grouped by treatment dose; greater time in restraint [FLU (168 hours)] significantly improved skilled reaching. **b** Forest plot of random-effects meta-analysis of recovery of spontaneous impaired forelimb use grouped by treatment dose; CIMT (FLU 56 hours + EX 7 hours) significantly increased spontaneous impaired forelimb use. **c** Forest plot of random-effects meta-analysis of locomotor recovery grouped by treatment dose; again, greater time in restraint [FLU (168 hours)] significantly improved locomotor function. Effect sizes in **a, c** presented as Hedge's *G* standardized mean difference (SMD) with 95% CI; effect sizes in **b** presented as mean difference (MD), percent change in impaired forelimb use, with 95% CI

Study or Subgroup	REHABILITATION Mean	SD	Total	CONTROL Mean	SD	Total	Weight	Std. Mean Difference IV, Random, 95% CI
**0-2500 METRES**								
**Tamakoshi 2018b (AE)**	-10.48	5.58	6.00	-16.65	11.40	2.67	12.0%	0.73 [-0.78; 2.24]
**Tamakoshi 2018c (AE)**	-18.26	9.21	6.00	-16.65	11.40	2.67	13.2%	-0.14 [-1.59; 1.30]
**Total (95% CI)**			**12.00**			**5.34**	**25.2%**	**0.27 [-0.77; 1.32]**
Heterogeneity: $Tau^2 = 0$; $Chi^2 = 0.67$, df = 1 (P = 0.41); $I^2 = 0\%$								
Test for overall effect: Z = 0.51 (P = 0.61)								
**2501-5000 METRES**								
**Auriat 2006 (AE)**	-11.04	10.93	17.00	-12.74	11.59	17.00	60.6%	0.15 [-0.53; 0.82]
**Tamakoshi 2018a (AE)**	-14.37	7.58	8.00	-16.65	11.40	2.66	14.2%	0.25 [-1.15; 1.64]
**Total (95% CI)**			**25.00**			**19.66**	**74.8%**	**0.17 [-0.44; 0.77]**
Heterogeneity: $Tau^2 = 0$; $Chi^2 = 0.02$, df = 1 (P = 0.90); $I^2 = 0\%$								
Test for overall effect: Z = 0.54 (P = 0.59)								
**Total (95% CI)**			**37.00**			**25.00**	**100.0%**	**0.19 [-0.33; 0.72]**
Heterogeneity: $Tau^2 = 0$; $Chi^2 = 0.72$, df = 3 (P = 0.87); $I^2 = 0\%$								
Test for overall effect: Z = 0.72 (P = 0.47)								

Std. Mean Difference IV, Random, 95% CI

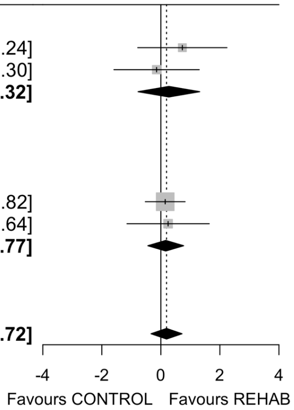

Fig. S14 Impact of treatment dose on recovery in AE interventions. AE interventions were divided into two treatment doses: 0-2500 metres and 2501-5000 metres. AE did not improve locomotor recovery. Effect sizes presented as Hedge's *G* standardized mean difference (SMD) with 95% CI

a

Study or Subgroup	REHABILITATION Mean	SD	Total	CONTROL Mean	SD	Total	Weight	Std. Mean Difference IV, Random, 95% CI
**EE [50-100 HOURS] + REACH [9-10 HOURS]**								
**Fedor 2022a [ER]**	7.67	2.36	13.00	6.64	2.96	9.00	9.2%	0.38 [-0.48; 1.24]
**Fedor 2022b [ER]**	9.79	4.39	13.00	7.32	3.84	11.00	9.7%	0.57 [-0.25; 1.40]
**Fedor 2022c [ER]**	8.88	2.60	23.00	8.47	3.69	11.50	11.4%	0.13 [-0.57; 0.84]
**Total (95% CI)**			**49.00**			**31.50**	**30.3%**	**0.34 [-0.12; 0.79]**
Heterogeneity: $Tau^2 = 0$; $Chi^2 = 0.65$, df = 2 (P = 0.72); $I^2 = 0\%$								
Test for overall effect: Z = 1.45 (P = 0.15)								
**EE [100-150 HOURS] + REACH [10 HOURS]**								
**Auriat 2009 [ER]**	7.71	2.56	16.00	4.79	2.98	16.00	10.8%	1.02 [0.28; 1.77]
**Auriat 2010a [ER]**	6.20	2.13	13.00	3.34	2.34	13.00	9.3%	1.24 [0.39; 2.09]
**Auriat 2010b [ER]**	10.12	2.28	16.00	7.10	2.68	16.00	10.6%	1.18 [0.42; 1.94]
**MacLellan 2011 [ER]**	8.20	4.88	16.00	3.72	3.93	14.00	10.5%	0.98 [0.21; 1.74]
**Caliaperumal 2014 [ER]**	11.41	3.74	11.00	6.61	2.78	11.00	8.1%	1.40 [0.45; 2.35]
**Total (95% CI)**			**72.00**			**70.00**	**49.4%**	**1.14 [0.78; 1.50]**
Heterogeneity: $Tau^2 = 0$; $Chi^2 = 0.62$, df = 4 (P = 0.96); $I^2 = 0\%$								
Test for overall effect: Z = 6.23 (P < 0.01)								
**EE [100-150 HOURS] + REACH [20 HOURS]**								
**Fedor 2022d [ER]**	9.96	2.92	21.00	8.47	3.69	11.50	11.0%	0.45 [-0.28; 1.18]
**EE [600 HOURS] + EX [1.5 HOURS]**								
**Auriat 2008 [ER]**	37.98	26.55	12.00	49.21	30.58	10.00	9.3%	-0.38 [-1.23; 0.47]
**Total (95% CI)**			**154.00**			**123.00**	**100.0%**	**0.69 [0.35; 1.03]**
Heterogeneity: $Tau^2 = 0.13$; $Chi^2 = 16.15$, df = 9 (P = 0.06); $I^2 = 44\%$								
Test for overall effect: Z = 3.99 (P < 0.01)								

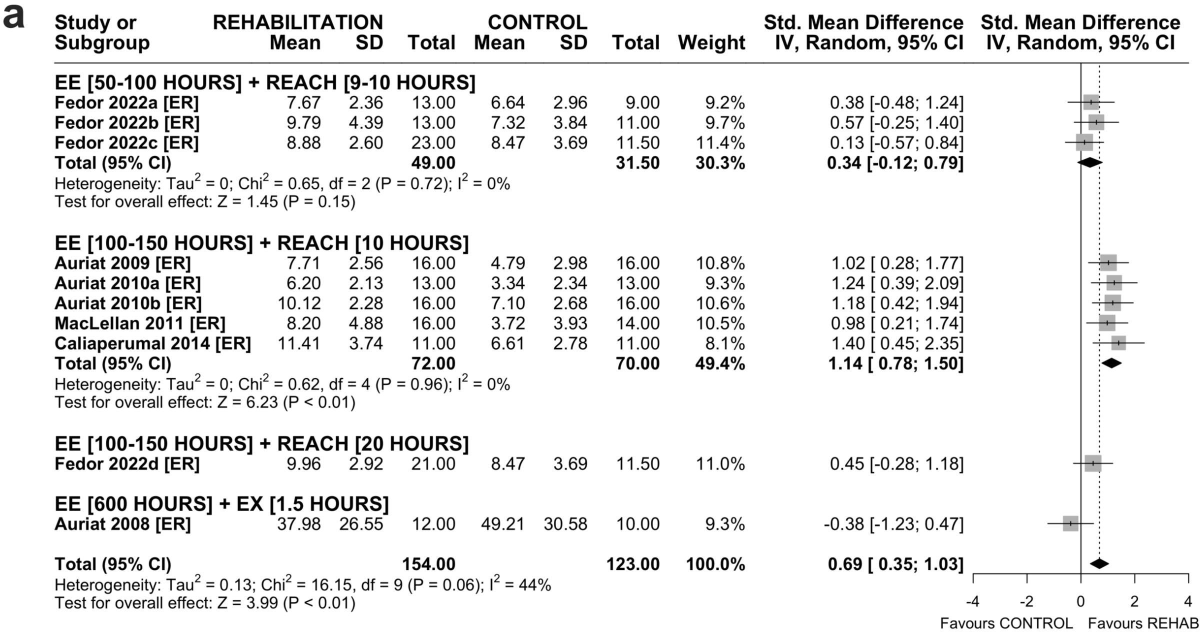

b

Study or Subgroup	REHABILITATION Mean	SD	Total	CONTROL Mean	SD	Total	Weight	Std. Mean Difference IV, Random, 95% CI
**EE [50-100 HOURS] + REACH [9-10 HOURS]**								
**Fedor 2022a (ER)**	93.46	6.93	13.00	91.84	5.50	9.00	16.7%	0.24 [-0.61; 1.10]
**Fedor 2022b (ER)**	95.34	8.78	10.00	100.00	0.00	11.00	0.0%	
**EE [100-150 HOURS] + REACH [10 HOURS]**								
**Auriat 2009 (ER)**	85.41	11.04	16.00	80.20	14.68	16.00	18.9%	0.39 [-0.31; 1.09]
**Auriat 2010a (ER)**	-17.45	17.02	13.00	-34.38	17.52	13.00	17.2%	0.95 [0.13; 1.77]
**Auriat 2010b (ER)**	-10.65	12.04	16.00	-32.71	11.04	16.00	16.8%	1.86 [1.02; 2.71]
**Caliaperumal 2014 (ER)**	96.54	4.40	11.00	69.74	17.17	11.00	13.9%	2.06 [0.99; 3.13]
**Total (95% CI)**			**56.00**			**56.00**	**66.8%**	**1.26 [0.48; 2.04]**
Heterogeneity: $Tau^2 = 0.44$; $Chi^2 = 10.22$, df = 3 (P = 0.02); $I^2 = 71\%$								
Test for overall effect: Z = 3.17 (P < 0.01)								
**EE [600 HOURS] + EX [1.5 HOURS]**								
**Auriat 2008 (ER)**	24.02	9.89	12.00	15.87	14.89	10.00	16.5%	0.63 [-0.23; 1.50]
**Total (95% CI)**			**91.00**			**86.00**	**100.0%**	**0.98 [0.40; 1.56]**
Heterogeneity: $Tau^2 = 0.34$; $Chi^2 = 14.13$, df = 5 (P = 0.01); $I^2 = 65\%$								
Test for overall effect: Z = 3.30 (P < 0.01)								

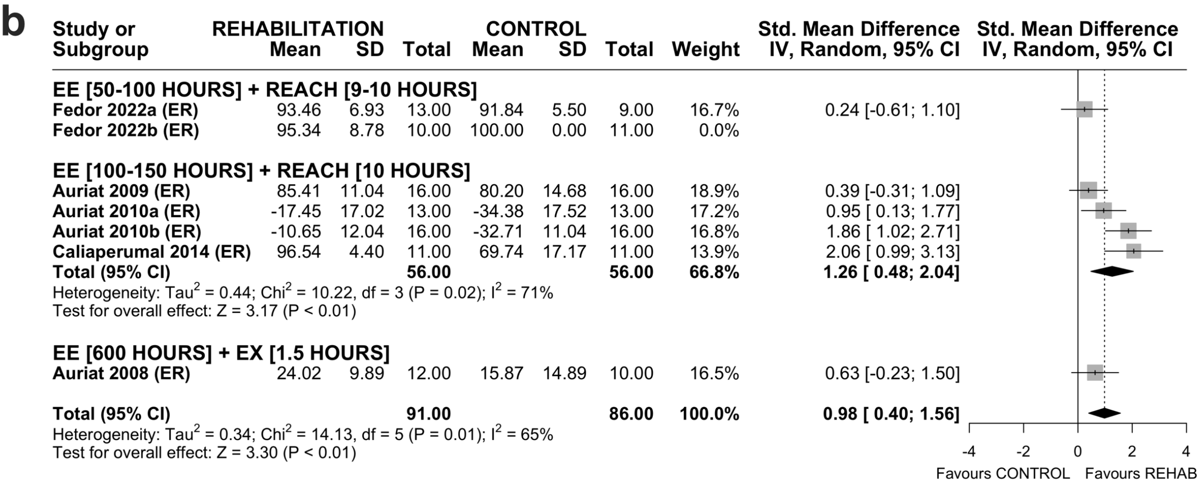

Fig. S15 Impact of treatment dose on recovery in ER interventions. ER interventions were divided into four treatment doses based on time in EE and REACH: EE (50-100 hours) + REACH (9-10 hours), EE (100-150 hours) + REACH (10 hours), EE (100-150 hours) + REACH (20 hours), and EE (600 hours) + EX (1.5 hours). **a** Forest plot of random-effects meta-analysis of skilled reaching recovery grouped by treatment dose; only the moderate dose group [EE (100-150 hours) + REACH (10 hours)] significantly improved skilled reaching. **b** Forest plot of random-effects meta-analysis of locomotor recovery grouped by treatment dose; again, only the moderate dose group [EE (100-150 hours) + REACH (10 hours)] significantly improved locomotor recovery. Effect sizes

presented as Hedge’s *G* standardized mean difference (SMD) with 95% CI

Study or Subgroup	REHABILITATION Mean	REHABILITATION SD	Total	CONTROL Mean	CONTROL SD	Total	Weight	Std. Mean Difference IV, Random, 95% CI
**CAMARADES SCORE [0-3]**								
**Auriat 2006 [AE]**	17.02	23.26	17.00	14.58	10.51	17.00	5.9%	0.13 [-0.54; 0.81]
**Santos 2013a [REACH]**	14.47	1.78	8.00	8.63	1.73	4.00	1.5%	3.05 [1.15; 4.95]
**Santos 2013b [WALK]**	10.44	0.76	8.00	8.63	1.73	4.00	2.5%	1.46 [0.07; 2.86]
**Ishida 2015a [FLU]**	28.30	21.78	8.00	7.10	13.80	9.00	3.7%	1.12 [0.08; 2.16]
**Ishida 2015b [FLU]**	21.30	7.84	6.00	12.30	18.00	9.00	3.7%	0.57 [-0.49; 1.63]
**Ishida 2016 [FLU]**	42.20	10.05	7.00	10.00	10.06	6.00	1.8%	2.98 [1.24; 4.72]
**Total (95% CI)**			**54.00**			**49.00**	**19.1%**	**1.32 [0.43; 2.20]**
Heterogeneity: $Tau^2 = 0.80$; $Chi^2 = 16.57$, df = 5 (P < 0.01); $I^2 = 70\%$								
Test for overall effect: Z = 2.92 (P < 0.01)								
**CAMARADES SCORE [4-6]**								
**DeBow 2003a [FLU]**	47.65	36.35	9.00	34.60	28.67	3.66	3.0%	0.35 [-0.88; 1.58]
**DeBow 2003b [CIMT]**	77.24	26.46	11.00	34.60	28.67	3.67	2.7%	1.49 [0.15; 2.82]
**DeBow 2003c [EX]**	42.83	21.19	9.00	34.60	28.67	3.67	3.0%	0.33 [-0.90; 1.55]
**MacLellan 2005 [CIMT]**	13.44	12.33	15.00	12.82	12.76	15.00	5.6%	0.05 [-0.67; 0.76]
**Auriat 2008 [ER]**	37.98	26.55	12.00	49.21	30.58	10.00	4.7%	-0.38 [-1.23; 0.47]
**Auriat 2009 [ER]**	7.71	2.56	16.00	4.79	2.98	16.00	5.4%	1.02 [0.28; 1.77]
**Auriat 2010a [ER]**	6.20	2.13	13.00	3.34	2.34	13.00	4.7%	1.24 [0.39; 2.09]
**Auriat 2010b [ER]**	10.12	2.28	16.00	7.10	2.68	16.00	5.3%	1.18 [0.42; 1.94]
**Ishida 2011 [FLU]**	7.20	5.09	8.00	2.50	3.90	9.00	3.8%	0.99 [-0.03; 2.02]
**MacLellan 2011 [ER]**	8.20	4.88	16.00	3.72	3.93	14.00	5.2%	0.98 [0.21; 1.74]
**Mestriner 2011a [REACH]**	65.37	9.91	12.00	47.43	12.09	6.00	3.3%	1.61 [0.46; 2.75]
**Mestriner 2011b [WALK]**	53.14	10.98	12.00	47.43	12.09	6.00	3.9%	0.48 [-0.52; 1.48]
**Caliaperumal 2014 [ER]**	11.41	3.74	11.00	6.61	2.78	11.00	4.2%	1.40 [0.45; 2.35]
**Total (95% CI)**			**160.00**			**127.00**	**54.9%**	**0.80 [0.47; 1.14]**
Heterogeneity: $Tau^2 = 0.15$; $Chi^2 = 20.3$, df = 12 (P = 0.06); $I^2 = 41\%$								
Test for overall effect: Z = 4.69 (P < 0.01)								
**CAMARADES SCORE [7-10]**								
**Nguyen 2008 [EE]**	36.20	18.99	14.00	25.82	18.96	16.00	5.5%	0.53 [-0.20; 1.26]
**Fedor 2022a [ER]**	7.67	2.36	13.00	6.64	2.96	9.00	4.7%	0.38 [-0.48; 1.24]
**Fedor 2022b [ER]**	9.79	4.39	13.00	7.32	3.84	11.00	4.9%	0.57 [-0.25; 1.40]
**Fedor 2022c [ER]**	8.88	2.60	23.00	8.47	3.69	11.50	5.6%	0.13 [-0.57; 0.84]
**Fedor 2022d [ER]**	9.96	2.92	21.00	8.47	3.69	11.50	5.5%	0.45 [-0.28; 1.18]
**Total (95% CI)**			**84.00**			**59.00**	**26.1%**	**0.41 [0.06; 0.75]**
Heterogeneity: $Tau^2 = 0$; $Chi^2 = 0.86$, df = 4 (P = 0.93); $I^2 = 0\%$								
Test for overall effect: Z = 2.33 (P = 0.02)								
**Total (95% CI)**			**298.00**			**235.00**	**100.0%**	**0.75 [0.50; 1.01]**
Heterogeneity: $Tau^2 = 0.17$; $Chi^2 = 41.51$, df = 23 (P = 0.01); $I^2 = 45\%$								
Test for overall effect: Z = 5.78 (P < 0.01)								
Test for subgroup differences: $Chi^2 = 4.93$, df = 2 (P = 0.08)								

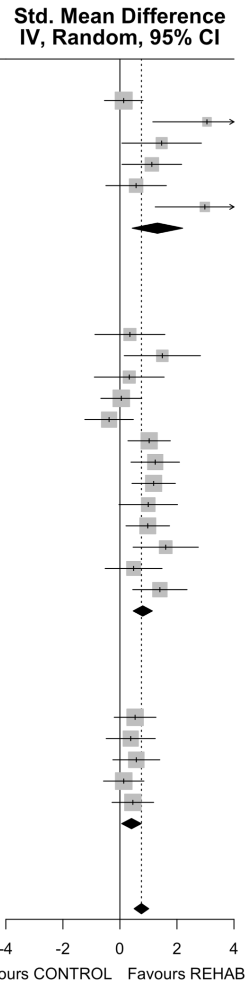

Fig. S16 Impact of study quality (CAMARADES score) on effect sizes reported in skilled reaching assessments. We observed a trend that as study quality increased, effect sizes decreased, and 95% CIs narrowed (test for subgroup differences p=0.08)

Search Strategy

Database: Academic Search Complete

TI = title search, AB = abstract search, SU = subject search, KW = keyword search

Rehab Terms (S1)

rehabilitation OR rehab OR exercise OR motor-therapy OR physical-therap* OR physiotherap* OR aerobic-training OR running OR walking OR treadmill* OR constraint-induced-movement-therapy OR mobilization OR mobilisation OR forced-use-therapy OR enrichment OR environmental-enrichment OR enriched-rehabilitation OR training OR reach* OR grasp*

S1: (TI (rehab terms)) OR (AB (rehab terms)) OR (SU (rehab terms)) OR (KW (rehab terms))

Stroke Terms (S2)

cerebral-hemorrhage* OR cerebral-haemorrhage* OR intracerebral-hemorrhage* OR intracerebral-haemorrhage* OR intracranial-hemorrhage* OR intracranial-haemorrhage* OR intracerebral-bleed OR cerebral-hematoma* OR hemorrhagic-stroke* OR haemorrhagic-stroke*

S2: (TI (stroke terms)) OR (AB (stroke terms)) OR (SU (stroke terms)) OR (KW (stroke terms))

Population Terms (S3)

rat OR rats OR mouse OR mice OR rodent* OR primate OR canine OR murine OR non-human OR animal-model

S3: ((TI (population terms)) OR (AB (population terms)) OR (SU (population terms)) OR (KW (population terms))

Search: S1 AND S2 AND S3

Search Strategy

Database: Medline

mp = title, abstract, original title, name of substance word, subject heading word, floating sub-heading word, keyword heading word, organism supplementary concept word, protocol supplementary concept word, rare disease supplementary concept word, unique identifier, synonyms

Rehab Terms (S1)

rehabilitation OR rehab OR exercise OR motor-therapy OR physical-therap* OR physiotherap* OR aerobic-training OR running OR walking OR treadmill* OR constraint-induced-movement-therapy OR mobilization OR mobilisation OR forced-use-therapy OR enrichment OR environmental-enrichment OR enriched-rehabilitation OR training OR reach* OR grasp*

S1: (rehab terms).mp.

Stroke Terms (S2)

cerebral-hemorrhage* OR cerebral-haemorrhage* OR intracerebral-hemorrhage* OR intracerebral-haemorrhage* OR intracranial-hemorrhage* OR intracranial-haemorrhage* OR intracerebral-bleed OR cerebral-hematoma* OR hemorrhagic-stroke* OR haemorrhagic-stroke*

S2: (stroke terms).mp.

Population Terms (S3)

rat OR rats OR mouse OR mice OR rodent* OR primate OR canine OR murine OR non-human OR animal-model

S3: (population terms).mp.

Search: S1 AND S2 AND S3

Search Strategy

Database: EMBASE

mp = title, abstract, heading word, drug trade name, original title, device manufacturer, drug manufacturer, device trade name, keyword, floating subheading word, candidate term word

Rehab Terms (S1)

rehabilitation OR rehab OR exercise OR motor-therapy OR physical-therap* OR physiotherap* OR aerobic-training OR running OR walking OR treadmill* OR constraint-induced-movement-therapy OR mobilization OR mobilisation OR forced-use-therapy OR enrichment OR environmental-enrichment OR enriched-rehabilitation OR training OR reach* OR grasp*

S1: (rehab terms).mp.

Stroke Terms (S2)

cerebral-hemorrhage* OR cerebral-haemorrhage* OR intracerebral-hemorrhage* OR intracerebral-haemorrhage* OR intracranial-hemorrhage* OR intracranial-haemorrhage* OR intracerebral-bleed OR cerebral-hematoma* OR hemorrhagic-stroke* OR haemorrhagic-stroke*

S2: (stroke terms).mp.

Population Terms (S3)

rat OR rats OR mouse OR mice OR rodent* OR primate OR canine OR murine OR non-human OR animal-model

S3: (population terms).mp.

Search: S1 AND S2 AND S3

Search Strategy

Database: CINAHL

TI = title search, AB = abstract search, SU = subject search

Rehab Terms (S1)

rehabilitation OR rehab OR exercise OR motor-therapy OR physical-therap* OR physiotherap* OR aerobic-training OR running OR walking OR treadmill* OR constraint-induced-movement-therapy OR mobilization OR mobilisation OR forced-use-therapy OR enrichment OR environmental-enrichment OR enriched-rehabilitation OR training OR reach* OR grasp*

S1: TI (rehab terms) OR AB (rehab terms) OR SU (rehab terms)

Stroke Terms (S2)

cerebral-hemorrhage* OR cerebral-haemorrhage* OR intracerebral-hemorrhage* OR intracerebral-haemorrhage* OR intracranial-hemorrhage* OR intracranial-haemorrhage* OR intracerebral-bleed OR cerebral-hematoma* OR hemorrhagic-stroke* OR haemorrhagic-stroke*

S2: TI (stroke terms) OR AB (stroke terms) OR SU (stroke terms)

Population Terms (S3)

rat OR rats OR mouse OR mice OR rodent* OR primate OR canine OR murine OR non-human OR animal-model

S3: TI (population terms) OR AB (population terms) OR SU (population terms)

Search: S1 AND S2 AND S3

Search Strategy

Database: PMC

TI = title search, AB = abstract search

Rehab Terms (S1)

rehabilitation OR rehab OR exercise OR motor-therapy OR physical-therap* OR physiotherap* OR aerobic-training OR running OR walking OR treadmill* OR constraint-induced-movement-therapy OR mobilization OR mobilisation OR forced-use-therapy OR enrichment OR environmental-enrichment OR enriched-rehabilitation OR training OR reach* OR grasp*

S1: TI (rehab terms) OR AB (rehab terms)

Stroke Terms (S2)

cerebral-hemorrhage* OR cerebral-haemorrhage* OR intracerebral-hemorrhage* OR intracerebral-haemorrhage* OR intracranial-hemorrhage* OR intracranial-haemorrhage* OR intracerebral-bleed OR cerebral-hematoma* OR hemorrhagic-stroke* OR haemorrhagic-stroke*

S2: TI (stroke terms) OR AB (stroke terms)

Population Terms (S3)

rat OR rats OR mouse OR mice OR rodent* OR primate OR canine OR murine OR non-human OR animal-model

S3: TI (population terms) OR AB (population terms)

Search: S1 AND S2 AND S3

References (Table S1)

1. DeBow SB, Davies MLA, Clarke HL, Colbourne F. Constraint-Induced Movement Therapy and Rehabilitation Exercises Lessen Motor Deficits and Volume of Brain Injury After Striatal Hemorrhagic Stroke in Rats. Stroke. 2003;34:1021–6.
2. MacLellan CL, Grams J, Adams K, Colbourne F. Combined use of a cytoprotectant and rehabilitation therapy after severe intracerebral hemorrhage in rats. Brain Res. 2005;1063:40–7.
3. Auriat AM, Grams JD, Yan RH, Colbourne F. Forced exercise does not improve recovery after hemorrhagic stroke in rats. Brain Res. 2006;1109:183–91.
4. Auriat A, Colbourne F. Influence of amphetamine on recovery after intracerebral hemorrhage in rats. Behav Brain Res. 2008;186:222–9.
5. Nguyen AP, Arvanitidis AP, Colbourne F. Failure of estradiol to improve spontaneous or rehabilitation-facilitated recovery after hemorrhagic stroke in rats. Brain Res. 2008;1193:109–19.
6. Auriat AM, Colbourne F. Delayed rehabilitation lessens brain injury and improves recovery after intracerebral hemorrhage in rats. Brain Res. 2009;1251:262–8.
7. Auriat AM, Wowk S, Colbourne F. Rehabilitation after intracerebral hemorrhage in rats improves recovery with enhanced dendritic complexity but no effect on cell proliferation. Behav Brain Res. 2010;214:42–7.
8. Takamatsu Y, Ishida A, Hamakawa M, Tamakoshi K, Jung C-G, Ishida K. Treadmill running improves motor function and alters dendritic morphology in the striatum after collagenase-induced intracerebral hemorrhage in rats. Brain Res. 2010;1355:165–73.
9. Ishida A, Tamakoshi K, Hamakawa M, Shimada H, Nakashima H, Masuda T, et al. Early onset of forced impaired forelimb use causes recovery of forelimb skilled motor function but no effect on gross sensory-motor function after capsular hemorrhage in rats. Behav Brain Res. 2011;225:126–34.
10. MacLellan CL, Plummer N, Silasi G, Auriat AM, Colbourne F. Rehabilitation Promotes Recovery After Whole Blood–Induced Intracerebral Hemorrhage in Rats. Neurorehabil Neural Repair. 2011;25:477–83.
11. Mestriner RG, Pagnussat AS, Boisserand LSB, Valentim L, Netto CA. Skilled reaching training promotes astroglial changes and facilitated sensorimotor recovery

after collagenase-induced intracerebral hemorrhage. Exp Neurol. 2011;227:53–61.
12. Kim MH, Lee SM, Koo HM. Ipsilateral and contralateral skilled reach training contributes to the motor function and brain recovery after left haemorrhagic stroke of rats. Brain Inj. 2012;26:1127–35.
13. Santos M V., Pagnussat AS, Mestriner RG, Netto CA. Motor Skill Training Promotes Sensorimotor Recovery and Increases Microtubule-Associated Protein-2 (MAP-2) Immunoreactivity in the Motor Cortex after Intracerebral Hemorrhage in the Rat. ISRN Neurol. 2013;2013:1–9.
14. Caliaperumal J, Colbourne F. Rehabilitation Improves Behavioral Recovery and Lessens Cell Death Without Affecting Iron, Ferritin, Transferrin, or Inflammation After Intracerebral Hemorrhage in Rats. Neurorehabil Neural Repair. 2014;28:395–404.
15. Tamakoshi K, Ishida A, Takamatsu Y, Hamakawa M, Nakashima H, Shimada H, et al. Motor skills training promotes motor functional recovery and induces synaptogenesis in the motor cortex and striatum after intracerebral hemorrhage in rats. Behav Brain Res. 2014;260:34–43.
16. Yong M-S, Hwangbo K. Skilled Reach Training Influences Brain Recovery Following Intracerebral Hemorrhage in Rats. J Phys Ther Sci. 2014;26:405–7.
17. Ishida A, Misumi S, Ueda Y, Shimizu Y, Cha-Gyun J, Tamakoshi K, et al. Early constraint-induced movement therapy promotes functional recovery and neuronal plasticity in a subcortical hemorrhage model rat. Behav Brain Res. 2015;284:158–66.
18. Ishida A, Isa K, Umeda T, Kobayashi K, Kobayashi K, Hida H, et al. Causal Link between the Cortico-Rubral Pathway and Functional Recovery through Forced Impaired Limb Use in Rats with Stroke. J Neurosci. 2016;36:455–67.
19. Takamatsu Y, Tamakoshi K, Waseda Y, Ishida K. Running exercise enhances motor functional recovery with inhibition of dendritic regression in the motor cortex after collagenase-induced intracerebral hemorrhage in rats. Behav Brain Res. 2016;300:56–64.
20. Tamakoshi K, Kawanaka K, Onishi H, Takamatsu Y, Ishida K. Motor Skills Training Improves Sensorimotor Dysfunction and Increases Microtubule-Associated Protein 2 mRNA Expression in Rats with Intracerebral Hemorrhage. J Stroke Cerebrovasc Dis. 2016;25:2071–7.

21. Tamakoshi K, Ishida K, Kawanaka K, Takamatsu Y, Tamaki H. Motor Skills Training Enhances α-Amino-3-hydroxy-5-methyl-4-isoxazolepropionic Acid Receptor Subunit mRNA Expression in the Ipsilateral Sensorimotor Cortex and Striatum of Rats Following Intracerebral Hemorrhage. J Stroke Cerebrovasc Dis. 2017;26:2232–9.
22. Tamakoshi K, Ishida K, Hayao K, Takahashi H, Tamaki H. Behavioral Effect of Short- and Long-Term Exercise on Motor Functional Recovery after Intracerebral Hemorrhage in Rats. J Stroke Cerebrovasc Dis. 2018;27:3630–5.
23. Sato C, Tanji K, Shimoyama S, Chiba M, Mikami M, Koeda S, et al. Effects of voluntary and forced exercises on motor function recovery in intracerebral hemorrhage rats. Neuroreport. 2020;31:189–96.
24. Tamakoshi K, Hayao K, Takahashi H. Early Exercise after Intracerebral Hemorrhage Inhibits Inflammation and Promotes Neuroprotection in the Sensorimotor Cortex in Rats. Neuroscience. 2020;438:86–99.
25. Xu Y, Yao Y, Lyu H, Ng S, Xu Y, Poon WS, et al. Rehabilitation Effects of Fatigue-Controlled Treadmill Training After Stroke: A Rat Model Study. Front Bioeng Biotechnol. 2020;8:1–17.
26. Tamakoshi K, Maeda M, Nakamura S, Murohashi N. Very Early Exercise Rehabilitation After Intracerebral Hemorrhage Promotes Inflammation in the Brain. Neurorehabil Neural Repair. 2021;35:501–12.
27. Fedor BA, Kalisvaart ACJ, Ralhan S, Kung TFC, MacLaren M, Colbourne F. Early, Intense Rehabilitation Fails to Improve Outcome After Intra-Striatal Hemorrhage in Rats. Neurorehabil Neural Repair. 2022;36:788–99.
28. Inoue T, Takamatsu Y, Nishio T, Soma K, Okamura M, Tohyama H, et al. Combined treatment with exercise and α5GABAAR inhibitor promotes motor function recovery after intracerebral hemorrhage. Neurosci Lett. 2022;766:136344.
29. Li Y, Lu T, Wei W, Lin Z, Ding L, Li Z, et al. Swimming Training Mitigates Neurological Impairment of Intracerebral Haemorrhage in Mice via the Serine-Threonine Kinase/Glycogen Synthase Kinase 3β Signalling Pathway. Neuroscience. 2022;501:72–84.
30. Tamakoshi K, Maeda M, Murohashi N, Saito A. Effect of exercise from a very early stage after intracerebral hemorrhage on microglial and macrophage reactivity states in

rats. Neuroreport. 2022;33:304–11.
